# Introducing the Coupled-Cluster Theory to the Amorphous World of Liquids and Their Thermodynamic Simulations

**DOI:** 10.1021/acs.jctc.5c01214

**Published:** 2025-09-18

**Authors:** Ctirad Červinka

**Affiliations:** Department of Physical Chemistry, University of Chemistry and Technology Prague, Technická 5, CZ-166 28 Prague 6, Czech Republic

## Abstract

Amorphous molecular materials are ubiquitous, spanning drugs, semiconductors, or solvents. Large predictive capabilities of quantum-chemical simulations of structural and thermodynamic properties and phase transitions for such amorphous materials have remained out of reach for a long time due to the related immense computational costs. This work introduces a novel fragment-based ab initio Monte Carlo (FrAMonC) simulation technique to the amorphous realm of molecular liquids and glasses. It aims at enabling thermodynamic simulations for amorphous molecular materials based on direct ab initio sampling and at minimizing the amount of a priori required empirical inputs for such simulations. Focus on individual cohesive interactions within the bulk, and their sampling from multiple first-principles potentials with a many-body expansion scheme enables the use of very accurate electron-structure methods for the most important cohesive features within the material. Even the coupled-cluster theory, the direct use of which is unprecedented for molecular simulations of thermodynamic properties for liquids, then becomes applicable to the description of bulk amorphous materials. Its incorporation in the proposed Monte Carlo simulations promises very high computational accuracy. Bulk-phase equilibrium properties at finite temperatures and pressures, such as density and vaporization enthalpy, as well as response properties such as thermal expansivity and heat capacity that are particularly challenging to predict accurately, are the observables targeted in this work. Superior computational accuracy of the introduced FrAMonC simulations is demonstrated for most target properties (liquid-phase densities, thermal expansivities, and gas–liquid differences in the heat capacities) when compared with established classical or quantum-chemical models that are commonly used to model such properties of bulk liquids.

## Graphical abstract

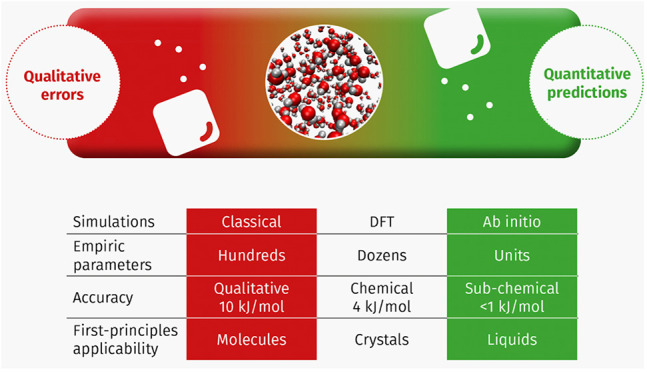

## Introduction

Amorphous forms of molecular materials include liquids and the glassy solid state, both of which lack any long-range regular structure. The significance of molecular liquids is obvious, as all Earth-bound life depends on water. Furthermore, countless chemical processes take place in solution, typically relying on a molecular solvent. For organic molecular solids, their amorphous forms have been overlooked for a long although they possess beneficial characteristics such as higher solubility inherent to all amorphous solids, being suitable for novel, more efficient drug formulations.^1^ On the other hand, growth of amorphous solid forms can be faster or preferential, such as in the case of organic-semiconductor thin films, despite that the amorphous forms typically exhibit worse optoelectronic properties.^2^ In both scenarios, gaining more insight from computational models into the stabilization or growth of amorphous organic solids would be very useful.

Computational chemistry has developed relatively straightforward approaches to model structural and thermodynamic properties or polymorphism of well-defined molecular crystals with quantum-chemical methods.^3^ Density-functional theory (DFT), being fortified with appropriate dispersion corrections,^4^ has become extremely popular for modeling cohesion of molecular crystals in various real-life applications, thanks to its very beneficial accuracy-to-cost ratio. Periodic DFT now enables approaching the chemical accuracy (enthalpic errors ≈ 4 kJ·mol^–1^) of predicted heats of sublimation,^5^ with errors in predicted crystal-phase densities reaching low percentage units,^6–8^ allowing to capture phenomena such as thermal expansion or material compressibility at least qualitatively.^9,10^ That computational accuracy proves, however, often to be insufficient to adequately capture subtle phenomena, such as polymorphism^3,11^ or predictions of vapor pressures^12,13^ where subchemical accuracy (errors <1 kJ·mol^–1^) is required.

Nevertheless, DFT has its flaws in modeling noncovalent interactions, especially when relying on the more feasible generalized-gradient approximation (GGA),^14–16^ which may obviously propagate into a misleading description of bulk crystals or liquids.^9,17,18^ Venturing beyond DFT, ab initio wave function theories are too computationally demanding to be applied directly with periodic boundary conditions to bulk systems.^19^ Therefore, fragment-based many-body expansion models,^20^ capable of fully exploiting the most accurate ab initio wave function methods,^21,22^ have emerged to refine predictions of cohesion in molecular crystals down to the subchemical accuracy of cohesion of molecular crystals, in terms of either their lattice energies, sublimation enthalpies,^5^ or even Gibbs free energies.^3,23^ Adopting these truly ab initio models seems indispensable to improve the computational accuracy for bulk crystals beyond the DFT framework.^24^

Concerning amorphous systems, on the contrary, there is no single well-defined equilibrium structure that could be exploited for their simulations. Large ensembles of at least hundreds or thousands of simulated molecules and similar numbers of bulk configurations are required to reasonably mimic liquids or glasses in silico. Such large system sizes then impart prohibitive costs, even to periodic DFT simulations. Extending the simulation scope from only ordered crystals to disordered amorphous liquids and molecular glasses, in fact, represents a massive challenge and complexity increase.

As a consequence, classical molecular simulations, giving up any explicit treatment of the presence of electrons and their chemistries, have been predominantly used for decades when thermodynamic or structural properties of molecular liquids and glasses were the aim. First-principles modeling of molecular materials in their amorphous state has remained prohibitively costly, being feasible only for the interpretation of very small systems and very fast (photo)­chemical processes therein.^25^

For liquids, any attempts for quantum-chemical molecular-dynamics (MD) or Monte Carlo (MC) simulations have been, for a long time, limited to using lower-rung dispersion-corrected DFT and very small simulated ensembles. Burdened with numerical issues such as the delocalization error,^14^ and frequently relying on a fortuitous error cancellation,^26^ it is no surprise that such DFT MD simulations, yet being extremely costly for liquids, can lead to qualitative failures when comparing structural subtleties^18^ or properties of multiple, relatively similar, forms of a molecular material.^27,28^ For example, it is very challenging to predict from the directly applied first-principles molecular simulations that water ice should float on liquid water, not sink.^29,30^ This difficulty relates to the common computational phenomenon of performing precise comparisons of inaccurately computed properties of multiple phases. That in turn imparts the need for reaching the highest possible computational accuracy of molecular simulations when such subtle phenomena are the aim.

Over the last three decades, a literature saga has evolved pursuing the goal of converging the predicted bulk liquid water density at ambient conditions to its real value,^28,29,32–36^ as illustrated in Figure 1. Starting with using classical force fields, more or less parametrized to reproduce experimental data,^37,38^ an agreement in terms of liquid water density within 1–2% can be reached.^32,33^ Adopting DFT without any dispersion corrections leads to massively underestimated water densities, whereas relying on dispersion-corrected DFT yields overestimated water densities with errors as large as 9% and 6% in the case of GGA and hybrid DFT functionals, respectively (both significantly lagging behind the force-field accuracy).^28^ Even venturing beyond DFT does not necessarily guarantee that the costly ab initio models would be competitive with the force fields, in terms of the accuracy. Among such expensive and sparse ab initio attempts, water density was predicted at 2% and 0.3% from experiment using the perturbative MP2 and RPA approaches, respectively.^29,35^ Exploiting the data-driven potentials trained with respect to ab initio cluster energies, such as MB-pol, enables minimization of errors in water density below 0.7% (or even less when nuclear quantum effects are accounted for).^27,39^

**Figure 1:**
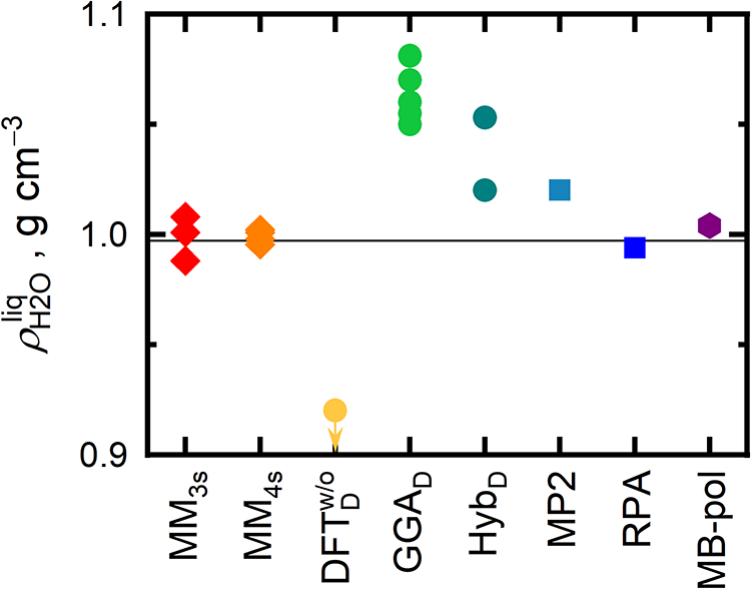
Computational difficulties related to the convergence of in silico predicted bulk liquid water density at ambient conditions to its experimental value (black horizontal line).^31^ Illustrative overview of the literature density predictions at 298 K and 100 kPa. This plot contains data reported earlier in various literature sources relying either on classical molecular simulations with 3-site^32^ or 4-site^33^ force-field models, dispersion-uncorrected DFT, various tiers of dispersion-corrected DFT,^28^ perturbative methods,^34,35^ and finally the most recent ab initio machine-learned potential MB-pol.^27^

The latest development of computational strategies for overcoming the large costs of any ab initio molecular dynamics for bulk materials is to develop machine-learned ab initio potentials that describe molecular interactions in the bulk, and subsequently, to run classical molecular dynamics relying on these first-principles data-driven potentials.^40^ Ab initio calculations of individual interactions of small molecular clusters or entire periodic simulation boxes can be used for data-driven training of these potentials.^41^

Finally, predictions of phase equilibrium-related phenomena involving the liquid phase, such as melting temperatures or saturated vapor pressures, correspond to the most challenging types of thermodynamic simulations due to the high sensitivity of the mentioned quantities to any computational noise in the underlying Gibbs energies.^42^ Modern machine-learning-based approaches have been demonstrated to provide reliable thermodynamic models of bulk water, including its complex behavior in the solid phase,^27,43^ catalyzed reactions,^44^ or biomolecular systems in aqueous solutions.^45^ Although such data-driven approaches bring tremendous computational savings with respect to performing the fully ab initio molecular-dynamics simulations, their main weakness is the absolute lack of any transferability of the potential among chemically distinct systems (especially those not included in the previous training), as can be illustrated by some pitfalls of machine-learned potentials for polymorph ranking of organic molecular crystals.^46^

Severe methodology, knowledge, and applicability gaps related to ab initio simulations of amorphous molecular materials thus still prevail. Aim of this work is to develop a novel simulation methodology that would concurrently fulfill the following aspects: (i) allow direct ab initio sampling of bulk molecular materials; (ii) enable use of state-of-the-art ab initio wave function methods; (iii) be computationally tractable; (iv) minimize the amount of a priori required empirical inputs including data-based potentials; (v) be consistently applicable for various systems including crystals, liquids, and glasses, allowing modeling also their phase behavior; (vi) model various ensemble types resulting primarily in predictions of simulations of structural and thermodynamic properties; (vii) be applicable to chemically diverse molecular materials; and (viii) enable the use of data-driven first-principles potentials in later stages of the simulations.

In particular, opening paths for adopting the coupled-cluster theory, which offers very high accuracy in terms of description of individual molecular interactions and large transferability across the chemical space,^47^ in ab initio simulations of molecular liquids, is the aim of this work. Numerous high-level ab initio wave function methods, including the coupled-cluster theory, lack the analytic formulation of their energy gradients, rendering them unsuitable for direct use in MD simulations. Novel ab initio Monte Carlo sampling schemes, where only energy (not gradients) evaluations take place for the bulk configurations, seem as an optimum choice for tackling the goals stated above. Although there have been studies on how to perform MC simulations of structure and thermodynamic properties of individual simple liquids (in particular water),^48^ adsorption in heterogeneous systems,^49^ or even phase equilibria^50^ from the first principles, those relied solely on DFT, random-phase approximation,^29^ or perturbative methods.^35^ In particular, nested MC simulations of liquefied noble-gas elements relying on machine-learned DFT-based potentials demonstrated a very promising strategy for increasing the accuracy of first-principles MC simulations of bulk liquids at modest costs.^51^

Nevertheless, incorporation of a truly ab initio wave function-based sampling in Monte Carlo simulations of bulk liquids and glasses is still due in this field. In this work, we propose a sophisticated triple-potential nested Monte Carlo scheme fulfilling the above-stated aspects and greatly contributing to the development of ab initio models for amorphous molecular materials. Finally, we demonstrate that such simulations of liquids are viable at the ab initio level, minimizing the a priori necessitated amount of empirical information. In particular, stress is laid on the systematic improvability of the computational scheme, and convergence of its results with respect to the quality of the ab initio potential toward experimental structural and cohesive data is analyzed.

## Computational Methodology

In terms of the computational methodology, our aim ventures far beyond the state-of-the-art in all-atom molecular simulations of liquids. Development of such a widely applicable computational method that relies on the coupled-cluster theory, representing the gold-standard model for noncovalent interactions in general,^47^ that would be applicable for all-atom simulations of bulk liquids, and that would be computationally tractable with the state-of-the-art hardware, represents a challenge long considered to be unfeasible to meet.

Our novel simulation approach relies on nested multipotential Monte Carlo simulations^52^ that combine sampling from up to three potentials with distinct accuracy and costs. At the bottom, a force field (FF) is used to generate nested (or inner MC loop) trial configurations of the simulated ensemble. With a certain period *P*, each such *P*th configuration is subject to refinements of its potential energy with more accurate and expensive (ab initio) methods, as illustrated in Figure 2. In that way, the configurations treated ab initio are sufficiently decorrelated, leading to a thorough sampling of the entire configurational space, and concurrently, are accepted at a sufficient rate ensuring an efficient computational workflow.^52^

**Figure 2:**
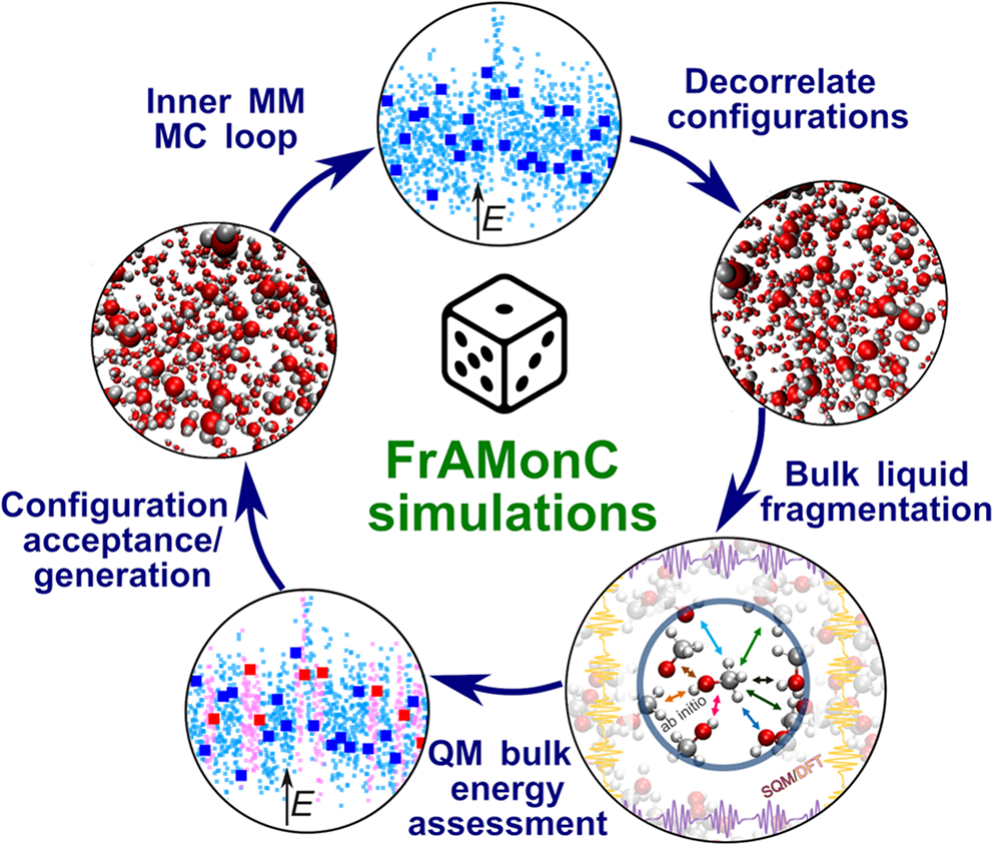
Schematic representation of the fragment-based ab initio Monte Carlo, i.e., FrAMonC, simulation workflow for bulk amorphous molecular materials.

Incorporation of the fragment-based ab initio many-body expansion of the cohesive energy^3,21^ of a bulk liquid into the nested Monte Carlo workflow represents the crucial computational advance achieved in this work. The overall cohesion of the bulk is expressed in terms of individual pair (and optionally also higher-order) interactions within a certain cutoff distance, and it is combined with long-range and many-body corrections evaluated with a medium-level method.^20,27^ Inspired by the state-of-the-art ab initio treatment of molecular crystals, this approach decomposes the complex problem of the bulk liquid containing too many electrons to be tractable ab initio into a set of many simpler tasks that can be treated with complex electron-structure theories.^3,21^

As long as one cannot rely on crystal-phase symmetry in simulations of liquids, the many-body expansions need to include significantly higher numbers of individual interactions. To maximize the computational efficiency and to maintain computational demands within a reasonable range, various features were implemented in our methodology, such as the nested MC sampling,^52^ temperature- and pressure scaling within the nested MC sequences,^52^ ab initio presampling of monomer geometries, conformational biasing,^53^ importance sampling focused ab initio on the first solvation shell,^3^ and others. Descriptions of these aspects and on-the-fly optimization of the related simulation setup are given in the Supporting Information, Section S1.

Performance of the novel simulation protocol is validated through modeling properties of three archetypal molecular liquids, namely water, methanol, and dimethyl ether. These materials were selected for this study as representatives of small-molecular materials where important interplay of electrostatic and dispersion interactions and optional hydrogen bonding occur. First, predictions of bulk densities and vaporization enthalpies are benchmarked against critically assessed reference data^31,54,55^ to assess the computational uncertainties in structural and enthalpic properties. Furthermore, predictions of response properties of the bulk, such as its thermal expansivity and heat-capacity difference between the vapor and liquid, are validated to provide a more stringent thermodynamic assessment of the performance of our novel methodology.

We employed on purpose multiple quantum-chemical methods of varying complexity within our nested Monte Carlo model to demonstrate its systematic improvability, which we consider as one of its main benefits, and also to present its current limitations serving as motivation for future work. Concerning the improvability and versatility of the methodology, an important aspect to be verified is whether the predicted results converge toward the experimental data with respect to the employed level of theory.

Taking the advantage of established simulation tools, implementation of the FrAMonC simulations consisted of the creation of an automated interface^56^ interconnecting the inner MC loops stochastically generating decorrelated configurations, the QM energy assessment enabling both periodic QM models and fragment-based ab initio many-body expansion models, and the outer QM-based configuration assessment, currently supporting *NVT* and *NpT* simulation ensembles. This newly implemented Bash shell interface periodically launches the underlying MC and QM tasks, collects their outputs, and evaluates their consequences.

Nested inner MC loops were performed in the Cassandra software, version 1.3.1,^57^ enabling simulation of molecular materials in various thermodynamic ensemble types with all-atom nonpolarizable FF models, namely, the TIP3P water model,^37^ and the OPLS model for methanol and dimethyl ether.^38^ As a representative of the extremely computationally efficient semiempirical QM models based on dispersion-corrected density-functional tight-binding, the third-order DFTB3 theory^58^ was selected, and such calculations were performed in the DFTB+ code, version 23.1.^59^ The 3ob parametrization,^60^ the post-hoc D4 dispersion correction,^4^ and the Hubbard U-parameter augmentation of the self-consistent charge Hamiltonian were used in all DFTB calculations. This model is further referred to as DFTB, and it was largely used to validate the functionality of the developed QM MC interface.

As a representative of lower-tier GGA DFT methods, the PBE functional^61^ was selected. The underlying periodic PBE jobs were run in VASP, version 6.4.2,^62^ along with the PAW formalism,^63^ hard PAW potentials,^64^ a 600 eV plane-wave kinetic-energy cutoff, D3­(BJ) dispersion model,^65^ and sampling the Γ-point only. This model is further referred to as PBE.

Following a QM:QM philosophy,^3,7,9^ the FrAMonC simulations were run using either the explicitly correlated^66^ perturbative RI-MP2-F12 theory with a modest cc-pVDZ basis set and the resolution of identity approximation,^67^ or the domain-based localized pair-natural orbital (DLPNO) formulation^68^ of the coupled-cluster CCSD­(T) theory, extrapolated to the complete basis set,^69^ as the high-level methods. All such MP2 or DLPNO–CCSD­(T) calculations were run in the ORCA code, version 6.0.^70^

FraMonC simulations also used either DFTB3-D4 or PBE-D3­(BJ) as the medium-level methods within the fragment-based ab initio many-body expansion of the bulk energy. The latter medium-level calculations exploited Gaussian-type orbitals (GTO) supplied with the pob-TZVP-rev2 basis set,^71^ as implemented in the code Crystal 23,^72^ allowing a more efficient computational workflow through the many-body expansion of the bulk energy. The FrAMonC model generally assumed a cutoff distance of 4 Å for an explicit high-level dimer treatment. Names of these particular FrAMonC setups are abbreviated in the following text as follows: MP2:DFTB, MP2:PBE, CC:DFTB, and CC:PBE, always reflecting both the high-level and medium-level theory employed in the many-body energy expansion.

Note that none of the current models explicitly account for nuclear quantum effects due to the large costs required for their treatment, although their impact on structural or chemical properties of liquids with high counts of protic hydrogen atoms at low temperatures can be non-negligible.^34,39^

The size of the simulation boxes was designed keeping in mind the affordability of the related computational costs. For simulation boxes mimicking the liquid, inclusion of that many molecules was targeted so that the total number of atoms in the box ranges from 300 to 360. Simulation boxes mimicking the ideal-gaseous vapor phase contained a single molecule surrounded by sufficient void space. More information on the computational setup and its validation can be found in the Supporting Information, Section S1.

## Results and Discussion

### Structural Properties

Comparison of liquid-phase densities calculated in this work with low-uncertainty reference densities at selected temperatures^31,54,55,73^ is shown in Figure 3a–c. Results of classical FF-based MC simulations are also included there to demonstrate that such all-atom models, often parametrized in the past to reproduce experimental data on bulk densities or heats of vaporization,^37,38^ usually reach a very good performance in terms of bulk densities (errors below 0.015 g cm^–3^) of materials covered by the parametrization. Such an accuracy is generally difficult to outperform by quantum-chemical models that were not trained to reproduce such empirical information. Running the QM MC scheme with DFTB or PBE theories yields larger errors in calculated bulk densities (up to 0.030 g cm^–3^) than the classical simulations relying solely on the FF. This demonstrates that the larger predictive power and transferability of the lower-cost QM methods often come at a price of lower accuracy when compared to the results of system-specific or data-based FF models. Still, the observed performance of the DFTB or PBE density predictions can be assessed as reasonable in this case. Also note that the underestimated bulk density from the PBE model (except water) is consistent with the typical behavior of this level of theory when dealing with quasi-harmonic predictions of the density of organic molecular crystals.^6,7,9^

**Figure 3:**
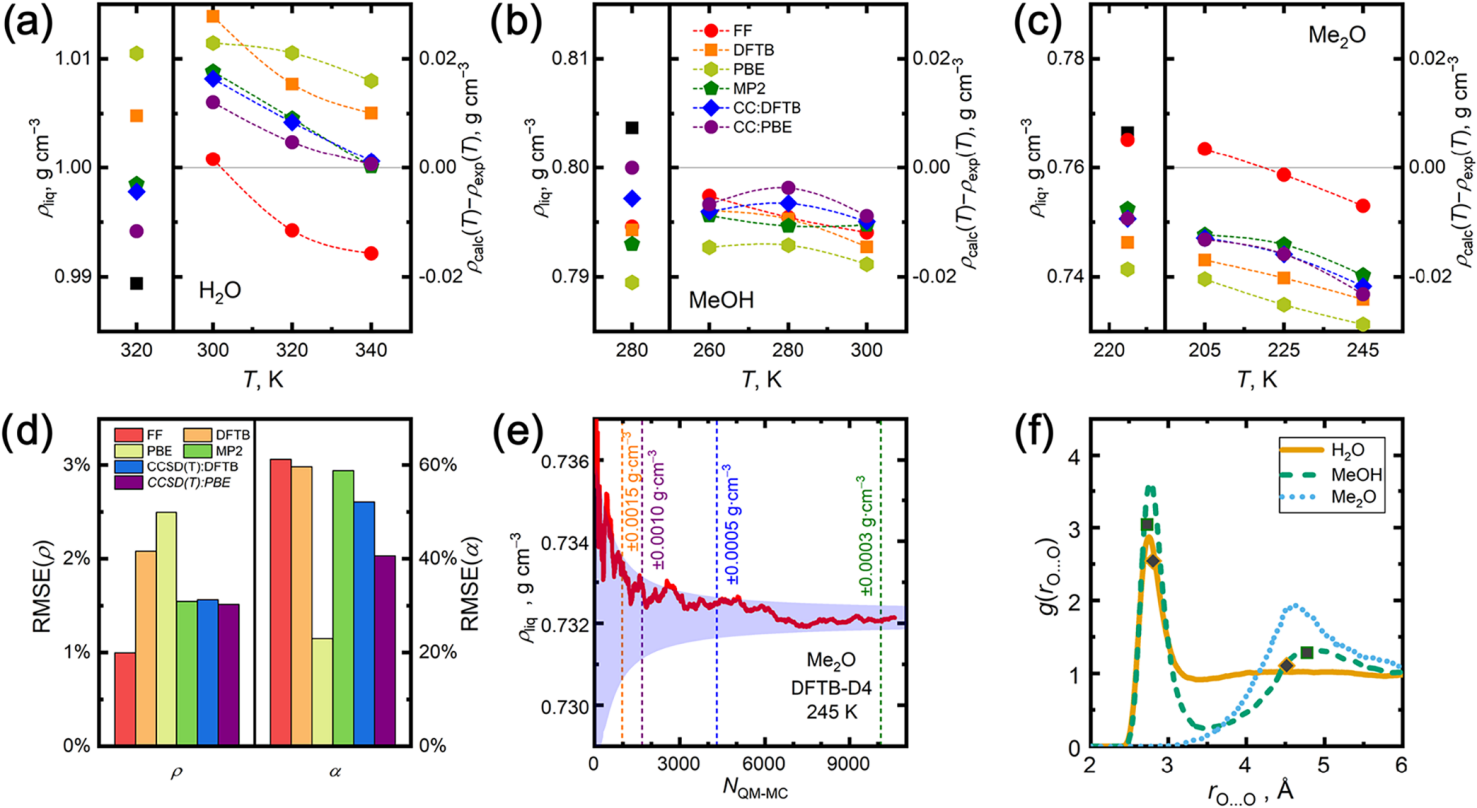
Overview of simulated structural properties of bulk liquids: (a–c) calculated bulk liquid densities (*ρ*) at ambient pressures and selected temperatures and their deviations from literature reference values taken from refs 31,54,55,73 for water, methanol, and dimethyl ether, respectively; (d) statistics on root-mean-squared relative errors (RMSE) of calculated densities and thermal expansivities (*α*); (e) example of a cumulative running average of ρ within a QM MC simulation of dimethyl ether at 245 K along with numbers of QM-accepted configurations required to converge the sampling uncertainties of *ρ* below the given thresholds; (f) radial distribution functions *g*(*r*) for O···O noncovalent contacts within first two solvation spheres extracted from FrAMonC simulations at the DLPNO–CCSD­(T):DFTB composite level of theory and experimental coordinates culled from ref 74 of the first two *g*(*r*) maxima (gray diamonds and squares for water and methanol, respectively).

When the FrAMonC model was fully deployed and individual pairwise interactions of proximate molecular pairs, forming the first solvation shell in the bulk phase, were treated ab initio, significant improvements over the DFTB or PBE results were reached. The current MP2:DFTB model brought liquid-phase densities with an accuracy approaching that of FF-based simulations (errors below 0.020 g cm^–3^). Replacing the MP2 treatment of proximate dimers with a more accurate DLPNO–CCSD­(T) model then leads to further improvements of the predicted densities (relative errors around 1.5%), which are already competitive with the FF-based results, although no model training to empirical data was required in this case. This systematic convergence of the FrAMonC simulation results to the real material behavior is further visualized in Figure 3d, depicting the root-mean-squared relative errors (RMSE) of the calculated densities.

Considering the thermal expansion of a bulk molecular liquid, its predictions represent a very stringent test for any computational model, as one needs to properly balance the variation of noncovalent interactions with the molecular separation with the amount of thermal energy that is available at individual temperatures. In particular, classical force fields are shown in Figure 3d to yield the largest RMSE of computed thermal expansivity coefficients, indicating limitations in their earlier parametrization procedures. Adopting models closer to the ab initio pole within our theoretical hierarchy, the accuracy of the predicted thermal expansion improves appreciably. Investing additional computational resources to use a more rigorous theory within the FrAMonC scheme, both in its high-level (CC vs MP2) and medium-level (PBE vs DFTB) regimes, proves to be worth the effort. Estimated CC:PBE results, representing the most sophisticated electron-structure theory considered in this work, are then the most accurate structural data set among all of the assembled FrAMonC bulk-liquid densities and thermal expansivities, illustrating a systematic improvement of the computational model and its convergence toward the reality. Note that capturing the thermal expansivity of a molecular material within 40% is an extremely challenging achievement to reach, even for state-of-the-art first-principles models of molecular crystals.^7,9^

Figure 3e further displays an uncertainty analysis important for designing the lengths and costs of FrAMonC simulations, indicating the number of configurations to be sampled at the QM level to converge the sampling uncertainty in bulk density below the desired level. While around a thousand configurations suffice to reach 1.5 kg·m^–3^ statistical-sampling uncertainty, being comparable with routine density measurements,^75^ averaging over significantly more than ten thousand configurations would be needed to minimize this MC sampling uncertainty to match the sub-0.1 kg·m^–3^ experimental state-of-the-art.^31^

Finally, the presented FrAMonC simulations also enable the analysis of the microscopic structure of bulk liquids. Examples of oxygen–oxygen radial distribution functions *g*(*r*) computed at the CC:DFTB level for the target liquids are given in Figure 3f. It illustrates that such FrAMonC simulations reasonably capture both the actual positions and amplitudes of the first two peaks of such *g*(*r*) functions, indicating a close agreement of the FrAMonC structural description of the two solvation shells in the liquids with experiment. A complete list of calculated structural results and a more detailed discussion of related aspects, revealing the difficulties of DFT-based simulations to correctly reproduce the *g*(*r*) features of bulk water^28,34^ and methanol,^76^ are given in the Supporting Material, Section S2.

### Cohesive Properties

Apart from the encouraging results of the simulated structural properties, it is also important to capture the cohesive properties of bulk materials to model their thermodynamic behavior properly. Vaporization enthalpies Δ_vap_ *H*, which are accessible experimentally with low uncertainties within the sub-kJ·mol^–1^ range,^77^ are directly linked to the extent of bulk cohesion of a bulk liquid, thus being an optimum observable for this enthalpic benchmarking. Figure 4a–c depicts the calculated Δ_vap_ *H* trends and reference experimental data for water,^31^ methanol,^78^ and dimethyl ether.^55^ One can see that FF-based simulations are able to capture the experimental Δ_vap_ *H* within 2 kJ·mol^–1^, which is again a result of their parametrization, at least in part, considering experimental vaporization enthalpies.^37,38^

**Figure 4:**
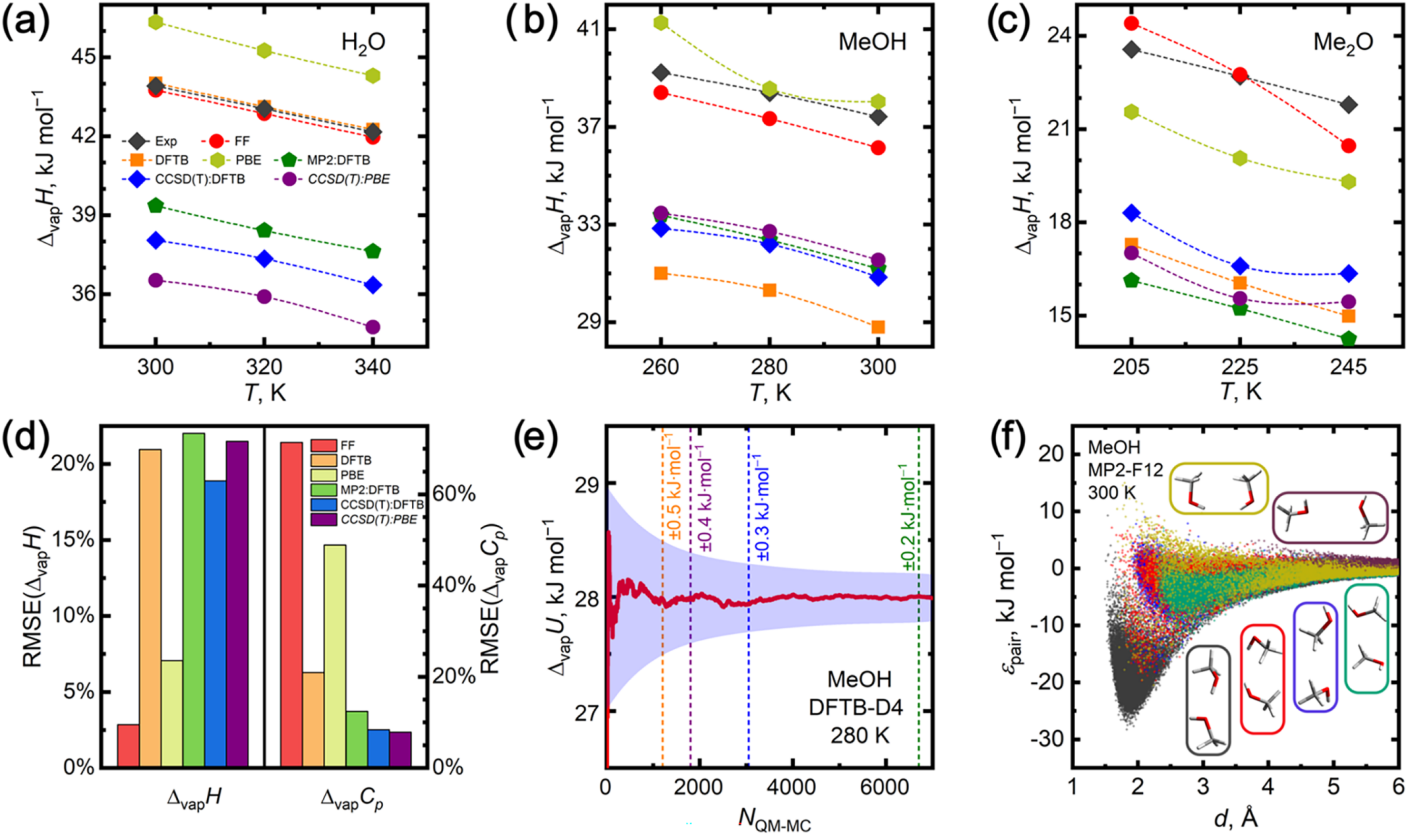
Overview of simulated cohesive properties of bulk liquids: (a–c) comparison of vaporization enthalpies (Δ_vap_ *H*) at selected temperatures with literature reference values for water, methanol, and dimethyl ether, taken from refs 31,54,55 respectively; (d) statistics on root-mean-squared relative errors (RMSE) of calculated Δ_vap_ *H* and differences between the isobaric heat capacities of vapor and liquid (Δ_vap_ *C*_*p*_); (e) example of a cumulative running average of Δ_vap_ *H* within a QM MC simulation of methanol at 280 K along with numbers of QM-accepted configurations required to converge the MC sampling uncertainties of Δ_vap_ *H* below the given thresholds; (f) pair-interaction energies extracted from FrAMonC simulation of bulk liquid methanol at 300 K, color-coded with respect to the atom types forming the closest contact within each dimer.

Figure 4a–c shows that it is not always straightforward to converge the ab initio models of the bulk cohesion within the desired chemical accuracy. Individual QM MC simulations, including the FrAMonC setups, yield Δ_vap_ *H* offset by up to 8 kJ·mol^–1^ (or 20%), unfortunately violating the desired chemical-accuracy goal. It is also noteworthy that there is no systematic convergence of the QM MC results on Δ_vap_ *H* to the experimental reality that would be similar to that described above for density results (with the partial exception of methanol). This indicates that some of the cheaper methods can benefit from fortuitous error cancellation in the description of individual components of bulk cohesion.

Among the expected factors corresponding to the remaining enthalpic offsets of theory from experiment that are summarized in Figure 4d, one can mention that ab initio treatment is employed for the first coordination shell only and that many-body interactions and long-range electrostatic terms are described with too low levels of theory to reach the (sub)­chemical accuracy. Long-range electrostatic contributions to the bulk cohesion extracted from periodic semiempirical QM methods or low-tier DFT models can impart substantial errors in the calculated cohesive energies.

On the other hand, analysis of the FraMonC simulation results and pair interactions extracted from the bulk at the MP2:DFTB level of theory indicates that extending the cutoff distance for an explicit high-level QM dimer treatment beyond 4 Å leads most likely only to sub-kJ/mol variations in the bulk energy. Also, one can largely rule out computational distortion of the bulk structure thanks to the above-presented close agreement of the predicted structure with the experimental data.

Concerning the very many-body expansion within the FrAMonC model, an explicit treatment of the three-body terms with a high-level QM theory is expected to increase the enthalpic accuracy appreciably, similarly to what has been demonstrated recently for the cohesion of molecular crystals and the importance of three-body interactions therein.^16^ In any case, error cancellation can play a substantial role in various many-body expansion models,^9,12,26^ which inherently aim at accurate summations of too many inaccurately calculated terms. That behavior can spoil any attempts at systematic convergence of such computational models,^79^ exactly as observed in this work for the vaporization enthalpy calculations.

Interestingly, plain PBE-D3­(BJ)/PAW treatment exhibits fair performance of Δ_vap_ *H* predictions for all three target materials, fulfilling the predictive chemical accuracy. Nevertheless, it stands out with its misprediction of the Δ_vap_ *H* slope with respect to temperature (i.e., given by the difference of the heat capacities of vapor and liquid Δ_vap_ *C*_*p*_), as shown in Figure 4d. On the other hand, FrAMonC simulations based on the CC:PBE model yield very realistic Δ_vap_ *C*_*p*_ values, being only 8% from the experiment. Such a result indicates that the FrAMonC simulations pass the otherwise stringent test of capturing the delicate variation of the material cohesion with respect to the temperature very well, but the predicted extent of cohesion is systematically underestimated. Also note that such a computational uncertainty of Δ_vap_ *C*_*p*_ is fully comparable with those of state-of-the-art experimental data, where uncertainties ranging from 0.1 to 0.2 kJ·mol^–1^ in terms of Δ_vap_ *H* typically correspond to about 10% uncertainty in the related Δ_vap_ *C*_*p*_ terms.^77^

An additional uncertainty analysis in Figure 4e reveals that generation of about a thousand FrAMonC configurations for both the vapor and the liquid suffices to converge the sampling uncertainty in Δ_vap_ *H* below 0.5 kJ·mol^–1^, whereas minimizing this value below 0.1 kJ·mol^–1^, to match the experimental state-of-the-art,^31,77^ is estimated to require averaging over more than ten thousand FrAMonC configurations.

Performing the presented FrAMonC simulations was necessarily accompanied by generating large libraries of pair-interaction energies of proximate molecules extracted from the instantaneous structures of bulk liquids. Figure 4f illustrates an example of the distribution of these pair-interaction energies in liquid methanol. It encompasses distinct magnitudes of the individual interaction types: hydrogen-bonded O_H_···H_O_, dispersion-bound O_H_···H_C_, and electrostatically repulsive O_H_···O_H_ contacts and too close H···H contacts dominated by exchange repulsion. Keeping this interaction plot (the width and the height of the area filled with sampled interactions in Figure 4f) in mind is important to realize how complex the interplay of the individual cohesive interactions holding the liquid together at the short-range actually is. Brownian motion within the liquid and temperature-induced structural defects then translate to the observed scatter of individual interactions in terms of both their contact distance and energy.

Such interaction libraries are an integral part of this work, as they represent a valuable material for future data-driven development of ab initio potentials. A complete list of the cohesive results and related detailed discussion can be found in the Supporting Information, Section S4. Computed pair-interaction libraries (containing over 2 million water dimers, nearly a million methanol dimers, and over 400 thousand dimethyl ether dimers, all at the DLPNO–CCSD­(T) and DFTB3-D4 levels of theory) can also be found in our data repository.^56^

### Computational Costs

A fundamental aspect of the FrAMonC simulations that needs to be disclosed is their significant computational cost. First, it is crucial to maintain a sufficiently high acceptance rate within the outer MC loop, where configurations are subject to their QM energy assessment, so that computational resources are not wasted for too frequent rejections of configurations within costly QM assessments. Since decorrelation of configurations to be assessed in the outer MC loop is granted via the nested inner MC loops, one does not need to maintain relatively low acceptance rates below one-third in nested multipotential MC simulations for an appropriate sampling of the entire phase space. Temperature and pressure scaling in the inner MC loops^52^ is finally adopted to maximize the agreements between energy distributions sampled using the low-tier classical potential in the inner MC loops and the high-tier QM potential in the outer MC loop. Figure 5a shows the acceptance rates observed on average within the QM assessment for individual materials and the levels of theory. Clear trends can be glimpsed from this plot, indicating that molecular complexity and an increased number of conformational degrees of freedom lead to a slight decrease in the QM acceptance rate going from water to dimethyl ether. Still, typical FrAMonC acceptance rates ranged between 75 and 90%, representing a very good computational efficiency corresponding to a productive load of the computing hardware. Interestingly, the periodic PBE model exhibited the lowest QM acceptance rate, whereas the DFTB led to the highest among the considered methods.

**Figure 5:**
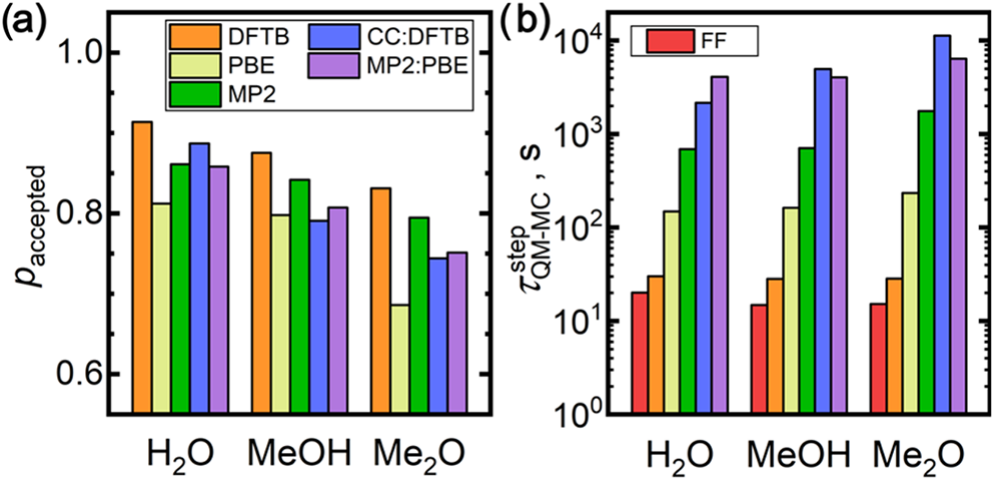
Overview of computational costs of QM MC and FrAMonC simulations for the target liquids: (a) average acceptance rates observed upon the QM configuration assessment (the outer MC loop); (b) average wall times required to perform a single QM configuration assessment (or to generate a single inner nested MC sequence at the FF level), values normalized to using 128 cores of the AMD 7H12 2.6 GHz CPUs.

Furthermore, the computer wall-time required to achieve a single QM configuration assessment is the crucial metric governing the overall computational demands of the FrAMonC simulations. Figure 5b compares the average wall times for a single QM configuration assessment at the considered levels of theory. While DFTB is only slightly more costly than the fully classical FF treatment, there is always an order-of-magnitude increase upon adoption of a more sophisticate QM MC model in the row DFTB < PBE < MP2:DFTB < MP2:PBE. For water with the smallest molecules in our test set, the costs of the long-range treatment at the PBE/pob-TZVP-rev2 are higher than the DLPNO–CCSD­(T) treatment of proximate dimers. With an increasing molecular size, the DLPNO–CCSD­(T) dimer costs prevail, as documented by the costs observed for dimethyl ether.

For instance, at the CC:DFTB level of theory, we observed that generation of a thousand accepted configurations within a periodic box of 100 water molecules, 50 methanol molecules, and 40 dimethyl ether molecules required about 680, 1750, and 4230 h on 128 CPU cores, respectively. Although the presented computational costs are large, an important message related to FrAMonC development should not be discarded. The underlying fragment-based many-body expansion of the bulk energy exhibits a perfect parallel efficiency thanks to the possibility of independently launching individual QM monomer, dimer, and other jobs at multiple computing nodes and their processor cores. Still, the computational costs of QM MC samplings that employ any QM method more costly than the semiempirical DFTB are significantly higher than the fully classical treatment.

Fortunately, once the libraries of proximate interactions within the bulk are created, those can be further exploited for data-driven development of ab initio potentials, greatly reducing the computational costs of any future simulations based on this FrAMonC sampling. The gold-standard method for modeling noncovalent interactions, the CCSD­(T) theory, known for a septic scaling of its cost with respect to system size, is absolutely inapplicable to periodic systems in its canonical form. However, through the novel efficient FrAMonC sampling protocol and using the DLPNO–CCSD­(T) formulation, the costs of such ab initio simulations of bulk liquids were optimized to only a quartic scaling of their cost with respect to the number of valence electrons in a molecule (when compared for similarly sized simulation boxes). More information about the computational costs is given in the Supporting Information, Section S3.

## Conclusions

A novel ab initio Monte Carlo simulation method, labeled FrAMonC, was implemented in this work, enabling simulations of structural and thermodynamic properties of molecular liquids and glasses, minimizing the need for a priori empirical input information. Relying on a multipotential nested Monte Carlo scheme and the fragment-based many-body expansion of the bulk potential energy, this achievement newly enables using as sophisticated treatment of electronic correlation and noncovalent interactions as the coupled-cluster theory also for simulations of amorphous molecular materials. Performance of the presented computational methodology was demonstrated in a benchmark of structural, cohesive, and response properties and their variation with respect to temperature, which usually represents a stringent test of any computational model. FrAMonC simulations exhibit a systematic improvement of their accuracy, with respect to the underlying electron-structure theory methods for most of the benchmarked properties. Plugging in the DLPNO–CCSD­(T):PBE level of theory, FrAMonC simulations exhibited an excellent performance in finite-temperature predictions of bulk liquid densities (errors at 1.5%), thermal expansivities (errors at 40%), and heat-capacity difference between the liquid and vapor phases (errors at 8%). There is still room for future development of the computational model due to the remaining offsets of the predicted vaporization enthalpies that still do not fulfill the initial chemical accuracy criterion. Further work on the FrAMonC scheme to suppress those remaining enthalpic errors could be directed to including an explicit high-level treatment of proximate trimers, using even more sophisticated levels of theory as the medium-level methods, such as hybrid DFT. Extending the cutoff radius for the explicit high-level treatment of proximate dimers in the many-body expansion model is less likely to succeed, at least for nonpolar materials. All such features will require a dramatic increase in the computational costs. However, current implementation and validation of the FrAMonC scheme carves a path to future developments relying on the incorporation of machine-learning ab initio potentials, arising from the libraries of pair interactions, as the high-level models within the FrAMonC scheme, greatly reducing the overall computational costs. Adoption of such machine-learned ab initio potentials seems indispensable to converge the description of material cohesion to the subchemical accuracy or to extend the FrAMonC applicability to real-life organic materials consisting of large molecules due to the large costs of the mentioned improvements. As well, future development of the FrAMonC model is expected to enable more challenging ab initio Monte Carlo simulations of phenomena such as solid–liquid equilibrium and glass transitions, possessing a real impact on material design and technology development. Finally, although Monte Carlo simulations represent in general a well-established simulation strategy applicable across a wide range of conditions, it remains to be addressed whether the FrAMonC sampling and energy evaluation remain numerically stable even at extreme temperatures and pressures, frequently relevant to real applications of amorphous molecular materials and solutions.

## Supplementary Material



## Data Availability

All data related to this work are available either in the Supporting Information or in a public repository: https://github.com/CervinkaGroup/FrAMonC

## Notes

Detailed description of the computational methodology and tuning of its setup; description of the development of reference data; detailed overview and discussion of data calculated in this work; libraries of molecular interactions extracted from bulk liquid structure that were calculated in this work; and overview of computational costs (PDF)
