# Supplementary material for: Introducing the Coupled-Cluster Theory to the Amorphous World of Liquids and Their Thermodynamic Simulations
Source: J Chem Theory Comput. 2025 Sep 18;21(19):9868–78. doi: 10.1021/acs.jctc.5c01214 (PMC12529918; doi:10.1021/acs.jctc.5c01214)
Supplement: Supplementary file 1 [file ct5c01214_si_001.pdf]

Introducing the coupled-clusters theory to the amorphous world of liquids and their thermodynamic simulations

Supporting Information

Ctirad Červinka[1]*

[1] Department of Physical Chemistry, University of Chemistry and Technology Prague; Technická 5, CZ-166 28 Prague 6, Czech Republic.

*Corresponding author e-mail: ctirad.cervinka@vscht.cz

S1 Computational methodology

S1.1 General settings of classical MC simulations

This computational setup applies to the nested inner loop MC simulations. All elementary MC moves and their processing was performed in the software Cassandra, version 1.3.0.[1] The low-tier nested MC sequences and additional plain MC used for benchmarking purposes were performed at the level of all-atom non-polarizable force field (FF). For water, the TIP3P model[2] was selected for its simplicity and compatibility with implemented Cassandra features, whereas the other two target systems, methanol and dimethyl ether, were modeled using the OPLS model.[3] All intramolecular non-covalent interactions of atoms linked by 3 covalent bonds (1–4 interactions) were scaled by a factor 0.5. Since equilibrium density as a function of temperature was one of the computational targets, all MC simulation were performed for *NpT* ensembles. With respect to the developed ab initio MC schemes, size of the simulation box was limited to a minimalistic setting, containing 100, 50 or 40 molecules of water, methanol or dimethyl ether, respectively. These numbers of molecules were selected so that the equilibrated cubic box dimension of bulk liquid safely exceeds 12 Å at ambient pressure and over a relevant temperature range for all three target materials. In that case, cut-off distance for all pair interaction was set to 6 Å. Ewald summation was used to capture all long-range electrostatic interaction of atomic point charges beyond this cut-off using its default implementation in Cassandra.[1] Each MC simulation started by a prescreening of relevant conformations of monomers and their energies at the FF level, which enabled subsequent adoption of the configurational bias methodology[4] for an efficient sampling of molecular bond angles and dihedral angles in the bulk phase within the MC framework. In general, allowed MC moves included molecular translations, rotations, conformation regrowth[4] and variation of the simulation-box volume. A proposed trial configuration j generated from a previous configuration i was accepted with the well-known Metropolis acceptance criterion:[5]

$$\alpha_{i\to j} = \min\left[1, \frac{\pi_j^{\mathrm{LT}}\ q_{j\to i}^{\mathrm{LT}}}{\pi_i^{\mathrm{LT}}\ q_{i\to j}^{\mathrm{LT}}}\right], \qquad \text{(S1)}$$

where π stands for the weight of a state in a phase space sampled with a low-tier, LT, potential (classical FF), and q stands for a marginal probability of proposing a particular trial move within that LT sampling.

S1.2 Dual-potential nested MC simulations

Multi-potential sampling is a crucial idea behind any efficient implementations of QM MC workflows.[6] With a certain frequency, bulk configurations generated within classical MC (i. e. inner nested sequences) were subject to recalculation of their potential energy using a quantum-chemical method, in general referred to as the high-tier model, HT, throughout this work. Such a HT sampling is supposed to provide more realistic potential energies and the sampled configurations, and thus, more accurate

simulations of the bulk liquid in general. A modified acceptance criterion is then adopted for such nested MC simulations in a form:

$$\alpha_{i\to j} = \min\left[1, \frac{\pi_j^{\mathrm{HT}}\ \pi_i^{\mathrm{LT}}}{\pi_i^{\mathrm{HT}}\ \pi_j^{\mathrm{LT}}}\right], \quad \text{(S2)}$$

where one distinguishes probabilities of the configurations sampled by both LT and HT methods.[7] With respect to equation (S2), whole nested MC sequences treated classicaly since the last HT-assessed configuration are either accepted or discarded, and the MC workflow is repeated. Note that only the HT-accepted configurations form the outer MC sequence are included in construction of ensemble avarages of the target properties.

Within a nested simulation of a canonical ensemble (*NVT*), the acceptance criterion from equation S2 can be rewritten in terms of potential energy changes ΔU between individual configurations as:[7]

$$\alpha_{i\to j} = \min\left[1, \mathrm{e}^{-\beta \Delta U_{ij}^{\mathrm{HT}} + \beta_0 \Delta U_{ij}^{\mathrm{LT}}}\right]. \quad \text{(S3)}$$

The acceptance criterion in equation S3 in fact depends on the similarity between the LT and HT distributions, but it results in generating a true cannonic phase-space sampling at the HT level.[7] Since both models necesarilly yield distinct potential energies, temperature within the classical nested MC sequences can be scaled with a scalar constant to improve the agreement between the LT and HT energy distributions, leading as well to increasing the acceptance rate of the HT-treated configurations. As a result, a scale factor $\theta=\beta_0/\beta=T/T_0$ is introduced and its value needs to be determined for such nested MC workflow. Following the procedure established in ref. [7], an on-the-fly procedure is adopted during the equilibratoin stage of MC simulations, updating θ value every 10 steps of the outer (i. e. HT assessed) MC loop untill self-consistency of θ is reached.

Within a nested simulation of an isobaric-isothermal ensemble (*NpT*), the acceptance criterion has the following form:

$$\alpha_{i\to j} = \min\left[1, \mathrm{e}^{-\beta \Delta U_{ij}^{\mathrm{HT}} + \beta_0 \Delta U_{ij}^{\mathrm{LT}}} \cdot \mathrm{e}^{-\beta p \Delta V_{ij} + \beta_0 p_0 \Delta V_{ij}}\right], \quad \text{(S4)}$$

obviously depending also on volume changes ΔV between the sampled configurations. Similarly to the temperature scaling adopted in the nested MC sequences of the cannonical ensemble, both temperature and pressure are scaled within nested *NpT* MC sequences to maximize the agreement of LT and HT distributions. Another parameter $\eta=p_0/p$ is introduced for this purpose and it value is found self-consistently on the fly according the recipe developed in ref. [7].

Potential energy of the simulated ensembles was calculated using the below disclosed quantum-chemical approaches and established software tools. An in-house code FrAMonC was developed to

process these acceptance acriteria for the outer QM-treated configurations and to interconnect particular QM codes with Cassandra inputs and outputs.[8] Particular aspects of the nested MC setup, such as the length of the nested MC sequences or impact of the temperature and pressure scaling therein is discussed below.

S1.3 Density functional tight binding calculations

Density functional tight binding, DFTB, as a representative of semi-empiric QM methods, is a natural choice for a computationally simple and efficient approach to be used at first at the HT level within the current dual-potential MC simulations. Namely, the third-order DFTB3 model[9] was adopted in this work for periodic quantum-chemical calculations of the total electronic energy of simulated liquid samples. The Hubbard U-parameter augmentation of the self-consistent charge (SCC) Hamiltonian was used along with a damping (exponent of 4.05) for short-range interactions of hydrogen atoms.[9] The 3ob parametrization,[10, 11] aiming at reducing the overbinding of bulk materials and non-covalent interactions therein, and the D4 dispersion-correction model[12] were applied thoughout all the DFTB calculations as implemented in the DFTB+ code, version 23.1.[13] This computational model is referred to as DFTB hereafter. Note that we tested performance of this computational setup earlier for molecular crystals,[14] confirming its applicability for preliminary fast modeling of novel systems.

S1.4 Density functional theory calculations

Density functional theory, DFT, corresponds to another level of computational complexity for periodic QM calculations for bulk liquids. Due to the steeply rising computational complexity of periodic DFT calculations with respect to the system size, only the general-gradient approximation (GGA) level was assumed in this work among all the existing DFT functionals. Namely, the PBE functional[15] with D3(BJ) dispersion corrections,[16] as implemented in VASP, version 6.4.2,[17] was employed in this work. Basis sets were created within the projected-augmented wave (PAW) formalism[18] along with a 600 eV plane-wave kinetic energy cut-off and hard PAW potentials.[19] Due to the larger size of the simulated ensembles, including hundreds of atoms and spanning linear dimentsions over 12 Å, only the Γ-point from the Brillouin zone was explicitly sampled in this case. Such a computational setup has been extensively validated in our group to yield a reasonable accuracy-to-cost ratio for diverse molecular crystals.[20, 21, 22, 23] It will be referred to as PBE hereafter.

Since periodic DFT at the GGA level suffers from various computational flaws, dominated by the delocalization error,[24] adopting even a more sophisticate electron-structure model for the bulk liquid is due when high accuracy is targeted. Moving beyond the GGA level within DFT may impart minor improvements of the computational accuracy as evidenced in various benchmarks focusing on modelling the cohesion of molecular crystals.[21, 25] However, periodic hybrid DFT models are very impractical to model large ensembles mimicking bulk liquids due to their infavorable quartic or steeper

scaling, not to mention the massive computational inefficiency of using hybrid DFT functionals along with plane wave basis sets.

S1.5 Fragment-based ab initio calculations

Molecular materials represent archetypes of weakly interacting systems that can be efficiently modelled using fragment-based methods[26] and potential energy of their bulk phases can be expressed in terms of a many-body expansion (MBE) of monomer energies and interactions of individual molecules extracted from their structure:[27]

$$E_{\mathrm{bulk}} = \sum_{i} E_i^{\mathrm{mono}} + \sum_{i<j} \varepsilon_{ij}^{\mathrm{di}} + \sum_{i<j<k} \varepsilon_{ijk}^{\mathrm{tri}} + \dots \ . \qquad \text{(S5)}$$

For practical reasons, equation (S5) can be truncated after the pairwise term and the number of explicitly included pair interaction is governed by a certain radial cut-off distance. To avoid large bulk phase energy errors related to this truncation, all the remaining higher-order interactions and long-range pairwise interactions are accounted for via an embedding scheme.[27-29]

A simulation box with hundreds of molecules extracted from a bulk liquid sample would contain too many electrons to be treated with periodic ab initio wavefunction theories. However, the fragment-based methods enable to decompose such a complex problem into a set of simpler tasks – one can focus on the electron structure of individual monomers and on interactions of proximate molecular pairs, three-body or larger clusters that are extracted from the bulk structure.[29, 30] These smaller systmes can be modelled with advanced ab initio theories, from perturbative Møller-Plesset (MP2) methods up to the coupled-clusters theory. Such a fragment-based many-body exansion approach thus enables to employ highly accurate electron-structure methods even for condensed systems, and to systematically improve and converge description of material cohesion.

For crystals of polar or charged molecules, an important feature to be included in the model is an electrostatic embedding that accounts for the long-range (and optionally also many-body) interations.[28, 29, 31] Diverse models for this treatment of long-range regime have been described in the literature, ranging from mere point-charge embedding[29] or polarizable Amoeba force field,[32] over periodic Hartree-Fock[28] for strongly polar systems and periodic DFT calculations,[21, 22, 31, 33] to periodic local perturbative LMP2 method for very small systems.[34]

In general, this ab initio fragment-based approach has been developped and extensively exploited for modeling cohesion, sublimation or polymorphism of molecular crystals[26, 28-32, 35] where one can benefit from the three-dimensional symmetry of the crystal lattice, massively reducing the numbers of individual distinct molecular clusters the interactions of which to be explicitely calaculated.[36] Over the last decade, this many-body expansion of bulk phase energies have been exploited to develop machine-learned ab initio potentials enabling extensive simulations for large systems, liquids or phase equilibria.[37]

For liquids, the absence of a single well-defined structure, however, leads to the need to fragment the bulk structure at every step of the outer MC loop, and to identify individual interactions within each of those configurations. Considering the computational feasibility of such a fragment-based MBE model for amorphous materials is thus crucial. Exploiting earlier results of ab initio MBE models for molecular crystals,[14, 28, 29, 33] and various benchmarks of ab initio models of non-covalent interactions,[38] one can expect severe deviations of the pair interaction energies computed by cheaper QM methods (DFTB or DFT) or classical FF from the results of more advanced ab initio wave function methods only within the first and second solvation shells of the bulk structure. For common molecular crystals, the first solvation shell spans only 3 to 4 Å in terms of the closest atomic contacts of the molecular pair.[21, 22, 29, 31] Cheaper methods can be thus used relatively safely to capture interactions of more distant molecules in bulk liquid structure without any dramatic loss of accuracy. This key assumption enables to keep the numbers of explicitly treated dimers and their interactions within reasonable bounds, which in turn rationalizes the overall computational cost.

Currently introduced ab initio MC simulations use DFTB or PBE-D3(BJ), the letter using Gaussian atomic orbital basis set pob-TZVP-rev2,[39] as the medium-level method for the periodic model of the bulk liquid, accounting also for the long-range and many-body interaction terms. Conformation-sensitive monomer energies (E_i) and proximate pair interaction energies (ε_{ij}), extracted from instantaneous bulk structure, are modeled with a high-level method. In this work, two options for the HL method are compared. A cheaper and faster option relies on explicitly-correlated[40] second-order RIJK-MP2-F12 method,[41] including the resolution of identity approximation RIJK[42] expanding the two-electron MP2 integrals in an auxiliary basis set cc-pVDZ/C,[43] and using a modest cc-pVDZ-F12 basis set optimized for explicit correlation models.[44] This model is referred to as MP2 hereafter. The other option is more rigorous and aims at mimicking the gold standard for non-covalent interactions,[45] the coupled-clusters with iterative treatment of single and double excitations and a perturbative correction to tripple excitations, CCSD(T), extrapolated to the complete basis set (CBS).[46] Namely, its modern formulation consisting in the domain-based localized pair-natural orbital flavor, DLPNO-CCSD(T),[47] extrapolation towards the CBS[46] from the Dunning-type correlation consistent cc-pVDZ and cc-pVTZ atomic basis sets,[48] and the resolution of identity approximation with auxiliary cc-pVTZ/C basis sets[43] are used in this work. To maximize the reliability of the DLPNO approximation, VeryTightSCF and TightPNO convergence options in ORCA were always invoked.[49, 50] This model is referred to as CC hereafter. In both cases of MP2 and CC models, QM code ORCA, version 6.0,[50, 51] is used for ab initio treatment of the respective monomers and dimers.

Following the MBE protocol,[28] total potential energy of a simulated ensemble can be rewritten also as:

$$E_{\mathrm{liq}}^{\mathrm{HT}} = E_{\mathrm{liq}}^{\mathrm{MT}} + \sum_{i}\left(E_i^{\mathrm{HT}} - E_i^{\mathrm{MT}}\right) + \sum_{i>j}\left(\varepsilon_{ij}^{\mathrm{HT}} - \varepsilon_{ij}^{\mathrm{MT}}\right). \quad \text{(S6)}$$

The first term on the left side corresponds to the energy of the simulated ensemble from periodic medium-tier calculation which is subsequently corrected with high-tier ab initio energies for all unique monomers (second term on the left), and with high-tier ab initio interaction energies of all unique proximate dimers. Indeces i and j thus vary now over all molecules within the simulated ensemble, which is a principal difference from simulations of crystals where i suffices to run over all symmetry irreducible molecules only. A cut-off distance at 4 Å is imposed for the pair interactions to be explicitly corrected by the last term on the left side of equation S6 unless noted otherwise.

The total energy defined in equation S6 is then used to assess the nested MC acceptance criteria defined in equations S3 and S4. We introduce this novel computational model as the Fragment-based Ab initio Monte Carlo (FrAMonC) simulation approach. Remind that within the FrAMonC model, three levels of theory are combined for an efficient sampling from an ab initio distribution of the potential energies in the phase space. A low-level method, i. e. classical FF is used in the inner nested MC loops, then a medium-level method, i. e. DFTB or PBE-D3(BJ), is used for periodic QM treatment in the outer MC loop, and it is further fortified with a high-level fragment-based energy refinement using ab initio methods up to the DLPNO-CCSD(T) theory.

Note that FrAMonC results corresponding to the highest level of theory presented in this work, namely the DLPNO-CCSD(T):PBE-D3(BJ) composite approach, do not come from full-fledged FrAMonC simulations at that level. To save computational resources, post-MP2 correction terms to both densities and vaporization energies were extracted from full-fledged FrAMonC simulations performed at DLPNO-CCSD(T):DFTB3-D4 and MP2-F12:DFTB3-D4 levels of theory, which were further added to the results of full-fledged MP2-F12:PBE-D3(BJ) simulations. The rationale behind this approximative scheme is analogous to that in estimating large-basis-set CCSD(T) energies from large-basis-set MP2 calculations and post-MP2 corrections evaluated with small basis sets.[45] In this case, we suppose that the respective post-MP2 corrections to the bulk liquid density and vaporization energy are rather insensitive to the low-level method used in the FrAMonC scheme, similarly to the insensitivity of the mentioned post-MP2 electronic-energy correction.

S1.6 Implemented statistical ensembles

So far, isothermal-isobaric *NpT* and isothermal-isochoric *NVT* ensembles have been implemeted in the FrAMonC tool. The *NpT* ensemble has been use to simulate equilibrium densities and energies of bulk liquids at various temperatures, constraining their pressure at 100 kPa. The *NVT* ensemble has been used to simulate monomers in large void boxes, mimicking the ideal-gaseous vapor phase to be able to sample its energy, and subsequently the vaporization energy defined as:

$$\Delta_{\mathrm{vap}}U = U_{\mathrm{m}}^{\mathrm{gas}} - U_{\mathrm{m}}^{\mathrm{liq}}, \tag{S7}$$

where U_m^{gas} and U_m^{liq} stand for molar average total energies of the gas and liquid phases, respectively. Finally, the vaporization enthalpy can be derived from $\Delta_{vap}U$ assuming the ideal behavior of the gas phases:[52]

$$\Delta_{vap}H = \Delta_{vap}U + RT. \tag{S8}$$

S1.7 Selecting the length of nested low-tier MC sequences

Principal purpose of adopting the nested MC approach is to decorrelate individual system configurations that are subject to costly high-tier, i. e. QM calculations of their energy.[7] Generating nested MC sequences of configurations with a low-tier method, i. e. computationally cheap as a force field help to maximize the differences between the neighboring configurations that are to be treated with the high-tier method. Optimum length of these nested MC sequences should be then searched for considering statistical and cost aspects. Naturally, using a costly high-tier method represents a dominant contribution to the total amount of required computational time per high-tier MC step. Still, generating too long nested low-tier MC sequences gives also birth to additional non-negligible computational costs. On the other hand, too frequent high-tier MC sampling inevitably leads to repeated costly QM treatment of very similar configurations. A measure of this sampling efficiency has been introduced by Allen and Tildesley as the statistical inefficiency parameter s.[53] A particular value of s can be interpreted as if only every s^{th} sampled observable value brings a completely new information to the averaged ensemble. The block-averaging protocol described in ref. 53 was adopted in this work to estimate this statistical inefficiency following the equation:

$$s = \lim_{\tau \to \infty} \left(\frac{\tau \, \sigma^2\left(\overline{X_\tau}\right)}{\sigma^2\left(X\right)} \right), \tag{S9}$$

where $\sigma^2\left(X\right)$ and $\sigma^2\left(\overline{X_\tau}\right)$ represent the variance of a target observable calculated over the entire sampled ensemble, and a variance among averages $\overline{X_\tau}$ computed over shorter blocks of length τ extracted from the entire data set, respectively. In practical calculations, $\sigma^2\left(\overline{X_\tau}\right)$ is repeatedly evaluated for blocks of varying lengths and the final s value is then accessed via extrapolation of the s=s(1/τ) trend towards $1/\tau = 0$. See the original reference for more details on this procedure.[53]

A comparison of the relationship of this statistical inefficiency and computational time per high-tier MC step with the length of the nested MC sequences within DFTB MC simulations of the target liquids at selected temperatures is given in Figure S1. Using this relatively cheap semi-empiric DFTB3 method as the high-tier level of theory enabled extensive methodology testing at a reasonable computational cost.

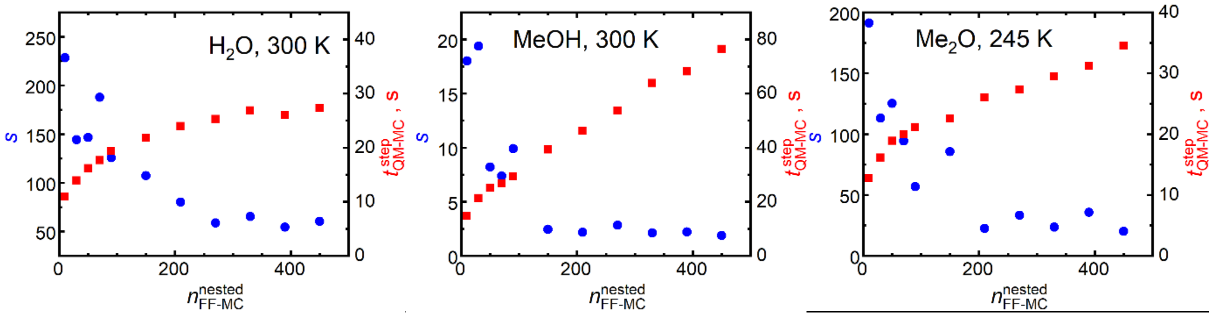

Figure S1. Impact of the length of low-tier nested inner MC sequences ($n^{nested}_{FF\text{-}MC}$, given in MC sweeps) on the statistical insignificance *s* of the accepted high-tier MC configurations and related mean computational time required to generate one accepted high-tier MC configuration.

Figure S1 shows that generating a nested inner MC sequence of 100 – 200 MC sweeps requires comparable computational time as one DFTB3 energy evaluation for the entire simulation box within periodic boundary conditions. Note that in our context, a MC sweep is defined as the number of elementary MC trial moves required to attempt every move type for every molecule.[1] Significant prolongations of this nested inner MC sequences then makes these low-tier calculations consume more computational resources than the the single-pooint high-tier energy evaluation, voting against excessive prolongations of these nested low-tier sequences. Concurrently, the statistical inefficiency of the high-tier treated MC configurations drops appreciably until the low-tier sequence length reaches some 175 – 225 MC sweeps. Any further decrease of this statistical inefficiency would require immense prolongation of the nested low-tier MC sequences, imparting unreasonably larger computational prerequisites. As a compromise between the cost and statistical efficiency considerations, low-tier MC sequences were set to span 270 MC sweeps for all target systems, which should grant reaching a fair level of decorreltaion of individual QM treated configurations, still at a reasonable computational cost.

S1.8 Temperature and pressure scaling in nested low-tier MC simulations

To maximize the acceptance rate of the expensive MC steps the energy of which is evaluated with the high-level method, i. e. QM, a procedure developped by Leiding and Coe to optimize nested MC simulations was adopted.[7] It requires analysis of the distributions of the high-level and low-level energies at individual MC steps and it aims at reaching as large similarity of both energy distributions as possible by scaling the actual temperature and pressure in the nested low-level nested MC sequences. Initial sections of the nested MC simulations thus serve to converge these scaling parameters θ and η (scaling the temperature and pressure, respectively, only in the low-level nested MC sequences). Exact definitions of both θ and η for an *NPT* simulation ensemble are given in ref. [7]. Figure S2 illustrates how θ and η evolve at the beginning of nested MC simulations of the three target liquids at selected temperature and at ambient pressure that combine DFTB3-D4/3ob and non-polarizable all-atom force field as the high-tier and low-tier methods, respectively. After a short dynamic section, both θ and η tend to converge to a constant value after several hundreds of high-tier MC steps. An optimum simulation

strategy thus consists in perfoming such an initial run that serves to find the optimum θ and η values to be used in the subsequent production MC runs. Figure S2 shows also that θ and η values remain rather close to unity, not leaving the (0.7,1.3) interval, indicating a reasonable physical similarity and compatibility of both the underlying low-tier and high-tier methods.

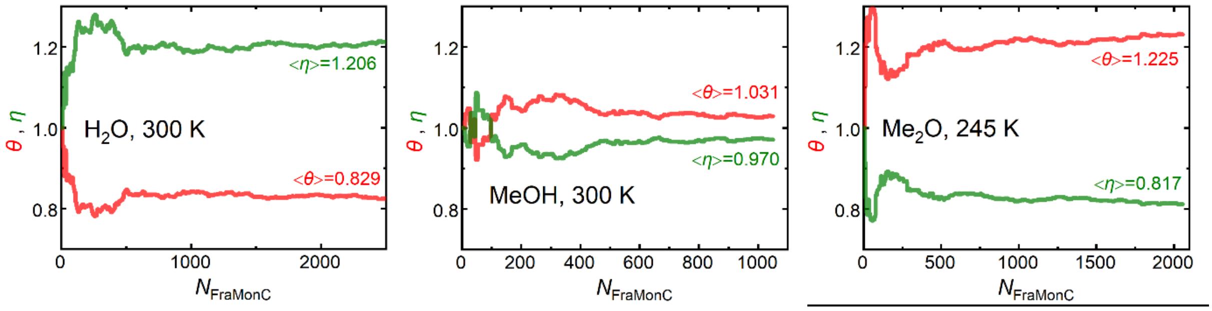

Figure S2. Time evolution of the scaling parameters θ and η that are used to scale the real temperature and pressure, respectively, in the nested force-field based MC sequences of DFTB&FF MC simulations.

Note that it is required to perform such initial optimization MC run at each individual temperature, pressure, combination of high-tier and low-tier methods and target chemical system. Table S1 lists θ and η values optimized for DFTB nested MC simulations of the three target liquids at various temperatures. Although the changes of the observed optimum θ and η values due to temperature variation are not massive, transferability of the once optimized θ and η to different conditions should be considered as very limited.

Table S1

Converged values of the scaling parameters θ and η that are used to scale the real temperature and pressure, respectively, in the nested force-field based MC sequences of DFTB&FF MC simulations at selected temperatures.

	H_2O, DFTB3-D4/3ob & FF	
T, K	θ	η
273.16	0.767	1.305
300.00	0.829	1.206
320.00	0.757	1.320
340.00	0.741	1.349
	MeOH, DFTB3-D4/3ob & FF	
T, K	θ	η
175.59	1.186	0.843
260.00	1.045	0.957
280.00	1.024	0.977
300.00	1.031	0.970
	Me_2O, DFTB3-D4/3ob & FF	
T, K	θ	η
131.66	1.228	0.814
205.00	1.199	0.834
225.00	1.199	0.834
245.00	1.225	0.817

Furthermore, the scatter of on-the-fly optimized *θ* and *η* values related to using the PBE functional in QM MC or the FrAMonC sampling, reaching up to 25% of the parameter value itself, is given in Table S2. Since there is no discernible trend among the *θ* and *η* values found for individual theories, these parameters cannot be thought of as transferable among distinct levels of theory.

Table S2
Converged values of the scaling parameters *θ* and *η* that are used to scale the real temperature and pressure, respectively, in the nested force-field based MC sequences of DFT-D/PAW&FF MC simulations at selected temperatures and using selected DFT functionals.

H_2O, 300 K		
T, K	*θ*	*H*
PBE-D3(BJ)/PAW & FF	1.117	0.895
MP2-F12:DFTB3-D4 & FF	0.921	1.085
MP2-F12:PBE-D3(BJ) & FF	1.040	0.962
CCSD(T):DFTB3-D4 & FF	0.947	1.108
MeOH, 300 K		
T, K	*θ*	*H*
PBE-D3(BJ)/PAW & FF	1.130	0.885
MP2-F12:DFTB3-D4 & FF	1.090	0.917
MP2-F12:PBE-D3(BJ) & FF	1.238	0.808
CCSD(T):DFTB3-D4 & FF	1.002	0.998
Me_2O, 245 K		
T, K	*θ*	*H*
PBE-D3(BJ)/PAW & FF	1.256	0.796
MP2-F12:DFTB3-D4 & FF	1.070	0.934
MP2-F12:PBE-D3(BJ) & FF	1.257	0.795
CCSD(T):DFTB3-D4 & FF	1.047	0.955

An important aspect is then to validate the promised efficiency improvements due to temperature and pressure scaling in the nested low-tier MC sequences. Using optimized *θ* and *η* values should result in a larger acceptance rate of the expensive high-tier MC steps without any losses of the quality of the phase-space sampling or distortions of the imposed temperature and pressure constraints in the high-tier MC sequence.[7] To investigate the impact of the *θ* and *η* scaling, short MC simulations using either optimized *θ* and *η* values or both parameter set to 1.00 were performed. The resulting acceptance rates are listed in Table S3. Apart from this particular methanol system, there is a visible trend indicating that the temperature and pressure scaling in the nested low-tier MC sequences can somewhat improve the overall computational efficiency of the nested QM & FF MC protocol. However, this improvement is rather minor as a very high acceptance rate of the high-tier MC steps is reached already with non-optimized *θ* and *η* values set to unity when reasonable low-tier models are used in the inner MC loop.

Table S3
Acceptance rates of the high-tier QM MC steps in simulations of the target liquids at selected temperatures, using either optimized scaling parameters θ and η or their values fixed to unity in the nested DFTB3-D4/3ob & FF MC simulations.

T, K	Optimized θ and η	$\theta = \eta = 1.00$
H_2O, 300 K	97.4 %	92.2 %
MeOH, 300 K	87.0 %	90.1 %
Me_2O, 300 K	86.5 %	81.5 %
Average	90.3 %	87.9 %

S1.9 Selecting an apropriate total length of nested MC simulations

Length of the outer MC loop of the simulations was adapted with respect to the desired statistical-sampling uncertainty of the target properties, and obviously bearing also in mind the computational affordability in the case of expensive QM MC steps. A preliminary statistical analysis of how the averages and associated sampling uncertainties of total energies and densities of bulk liquids simulated at the FF level was performed to gain an insight on the minimum necessary length of the subsequent production QM MC runs. Given the computational sophistication of the QM MC approach, especially when ab initio treatment of proximate pair interactions is involved, one would like also to reach sufficiently low sampling uncertainty to maximize the impact of the current simulations. These uncertainty thresholds to be targeted were primarily set to 0.5 kJ mol^{-1} and 1.5 kg m^{-3} for total energy and density of bulk liquids, respectively. Note that such values roughly correspond to the routinely reached uncertainties of experimental determinations of vaporization enthalpy and liquid-phase density.

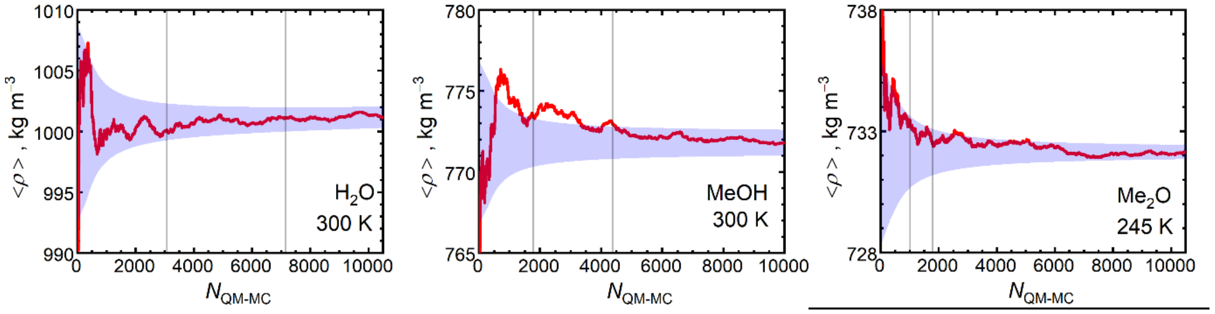

Figure S3. Average density of bulk liquid as a function of the number of high-tier QM treated MC configurations that were included in the averaging (N_{QM-MC}) from a long nested DFTB MC simulation. Blue-shaded areas depict the 67% confidence intervals for the average density derived from a DFTB treated MC sequence of a particular length. Vertical grey lines indicate the lengths of DFTB MC sequences required to converge the standard uncertainty of bulk density below 1.5 and 1.0 kg m^{-3}, respectively.

The block-averaging procedure[53] was applied for the entire MC sequence generated with the DFTB level of theory, gradually truncating it at selected lengths, to investigate how the real statistical sampling uncertainty of the mean bulk density varies with the MC sequence length. Figure S3 depicts the uncertainty trends that were obtained for the target bulk liquids, illustrating that thousands of MC

configurations to be treated with the high-tier theory are needed to converge the uncertainty of bulk density below the desired threshold. The exact length of these MC sequences depends on the system size with smaller molecules or atomic ensembles requiring longer MC sequences. For larger systems, current results indicate that generating around 1000 high-tier MC steps should be sufficient to reach the desired statistical accuracy. On the other hand, further minimization of the statistical uncertainty would be extremely demanding as generation of 10000 high-tier MC steps yields standard uncertainties of the densities around 0.6 kg m^{-3}.

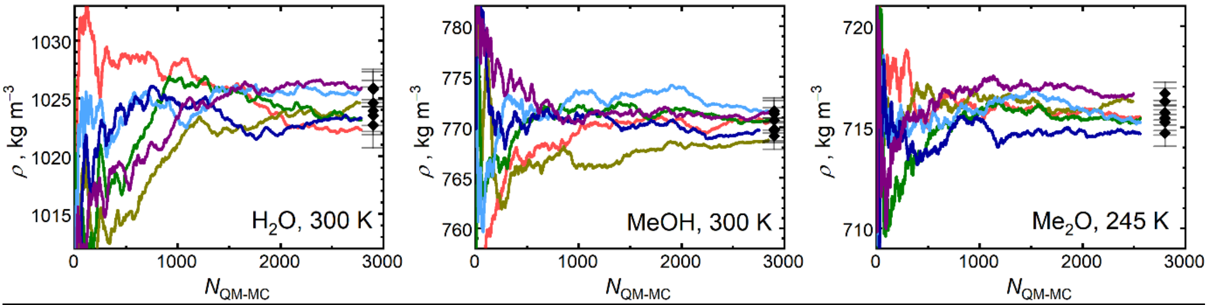

Figure S4. Mean bulk liquid densities obtained from DFTB MC simulations plotted as a function of the number of QM-accepted MC trial configurations. Individual colored curves represent independent simulation replica, the final averaged results of which are given with black diamonds.

Another aspect of performing equilibrium bulk phase simulations is the reproducibility of the results. To verify the reproducibility of bulk density results from nested QM MC simulations, six independent replica of each target systems were simulated using the same DFTB MC protocol, only starting from distinct initial random seed. Figure S4 shows that density averages of less than 500 QM-accepted configurations (1000 for water) are burdened by massive uncertainties and individual replica can yield largely different results. However, this situation improves appreciably when more than 1000 QM-accepted MC configurations are considered. Finally, including about 3000 configurations in the density averaging then leads to consistent results of individual replica that mutually agree within the ±1.5 kg m^{-3} standard uncertainties that were assigned to the mean densities of each individual simulation replica by its block averaging.

Generating multiple independent MC simulation replica, each spanning only a relatively small number of accepted configurations, can be beneficial to reach acceptably low sampling uncertainties for the most expensive FrAMonC simulations. For instance, results of the FrAMonC simulations relying on the MBE energy evaluation at the CC:DFTB and MP2:PBE levels of theory were averaged from four or five independent simulation replica, each spanning only low hundreds of QM-accepted configurations. Statistical-sampling uncertainty was in such cases estimated using the procedure described in ref. [54].

S1.10 Impact of the monomer geometry

The underlying MC software Cassandra that was used throughout this work to design and process all nested MC moves within the inner MC loop at the FF level of theory imposes constraints on all bond lengths in the simulated ensemble.[1] This geometric constraint implemented in Cassandra prevents wasting too much computational resources on MC sampling of populations of bond lengths the perturbation of which from the equilibrium length inevitably results in a steep energy increase, and thus also in too frequent rejections of such configurations. In this context, it is crucial to select an appropriate set of bond lengths related to particular molecular targets. To preserve the first-principles nature of the current QM MC simulations, monomer (and optionally dimer) geometries of the target compounds had been optimized in the gas phase using the same QM methods that were subsequently employed in the QM MC simulations. Such optimized geometries were obtained in the Gaussian 16 software, revision B.01[55] (and the aug-cc-pVTZ basis set[48] and D3(BJ) dispersion correction[16] for DFT calculations), with the exception of the semi-empirical DFTB3-D4/3ob calculation[10, 12] which was performed in the DFTB+ code, version 23.1.[13] This protocol yielded distinct monomer geometries for every level of theory considered with particular bond lengths varying appreciably among all the data sets. Since hydrogen-bonding in bulk usually affects the optimum bond lengths of adjacent functional groups, hydrogen-bonded dimer structures were optimized for water and methanol to capture also this aspect. Final bond lengths to be used for bulk simulations were then obtained by averaging the given bond lengths in the optimized monomer and in the optimized dimer. All bond lengths developed and used in MC simulations in this work are summarized in Table S4.

Table S4
Monomer bond lengths (*d*, values in Å) that were found in this work by geometry optimizations of isolated monomers (and optionally hydrogen-bonded dimers) and to be constrained in QM MC simulations of bulk liquid at the individual levels of theory. Experimental data are given for comparison.

				H_2O				
T, K	Exp. [56]	FF[2]	DFTB3	PBE	r2SCAN	B3LYP	MP2	CCSD(T)
d(O–H)	0.9580	0.9570	0.9617	0.9735	0.9652	0.9644	0.9638	0.9643
				MeOH				
T, K	Exp. [57]	FF[3]	DFTB3	PBE	r2SCAN	B3LYP	MP2	CCSD(T)
d(O–H)	0.9560	0.9450	0.9697	0.9737	0.9643	0.9641	0.9645	0.9644
d(O–C)	1.4270	1.4100	1.4055	1.4265	1.4200	1.4255	1.4219	1.4234
d(C–H)	1.0960	1.0900	1.1013	1.1017	1.0938	1.0923	1.0901	1.0936
				Me_2O				
T, K	Exp.[58]	FF[3]	DFTB3	PBE	r2SCAN	B3LYP	MP2	CCSD(T)
d(O–C)	1.4110	1.4100	1.4115	1.4173	1.4107	1.4111	1.4122	1.4144
d(C–H)	1.0943	1.0900	1.1019	1.1038	1.0955	1.0940	1.0925	1.0954

Furthermore, anharmonicity of the stretch vibrational modes is another factor that may affect the molecular geometry. For this purpose, relaxed scans of molecular potential energy along the stretching modes of relevant bonds in the target monomer molecules were performed along with calculations of

anharmonic vibrational frequencies in Gaussian. Morse potential was then fitted to the scanned potential energies and discrete energy levels of relevant vibrational stretching modes were used to compute the mean equilibrium bond length at 0 K (including the anharmonic impact of the zero-point energy) and at selected finite-temperatures.

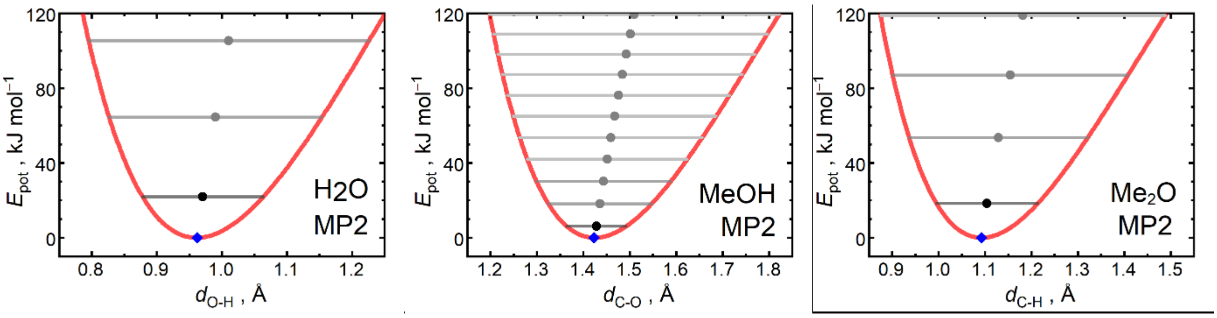

Figure S5. Potential energy of selected stretching vibrational modes obtained from relaxed scans of target monomers at the MP2/aug-cc-pVTZ levels of theory along with the energy levels of according Morse oscillators and mean bond lengths corresponding to individual energy levels (grey circles) and minimum of the potential energy surface (blue diamond).

Figure S5 illustrates the procedure followed to derive the mean bond lengths for individual vibrational levels. Since the excited vibrational states get barely populated at (sub-)ambient temperatures, the temperature-induced bond elongation is not pronounced in the temperature region that is relevant to simulate the target bulk liquids. At ambient conditions, the mean bond lengths exhibit variations in the order of at most 10^{-5} Å as temperature changes even for the C-O bonds, which is below a typical force-field resolution. Still, the zero-point vibrational phenomenon itself elongates the bond lengths from the position given by the potential energy minimum (i. e. optimized molecular geometries) by some 10^{-2} Å, being already a considerable effect, corresponding roughly to 1% of the bond length. Table S5 lists the bond lengths for the target molecules yielded by this anharmonic analysis of monomer vibrations.

Table S5
Monomer bond lengths (*d*, values in Å) at 0 K that were found in this work by anahrmonic analysis considering the potential energy shapes and anharmonic vibrational energies related to bond stretching modes that govern mean bond lengths of the anharmonic oscillators. Experimental data are given for comparison.

		H_2O			
T, K	Exp. [56]	DFTB3	PBE	MP2	CCSD(T)
d(O–H)	0.9580	0.9792	0.9832	0.9733	0.9738
		MeOH			
T, K	Exp. [57]	DFTB3	PBE	MP2	CCSD(T)
d(O–H)	0.9560	0.9803	0.9837	0.9742	0.9741
d(O–C)	1.4270	1.4089	1.4340	1.4256	1.4272
d(C–H)	1.0960	1.1096	1.1125	1.0990	1.1026
		Me_2O			
T, K	Exp.[58]	DFTB3	PBE	MP2	CCSD(T)
d(O–C)	1.4110	1.4165	1.4214	1.4160	1.4183
d(C–H)	1.0943	1.1103	1.1138	1.1043	1.1073

Given the appreciable bond-length differences among individual levels of theory that reach up to 0.03 Å in the extreme cases (PBE yielding the longest and FF containing the shortest), non-negligible impact of the selection of a particular bond-length setting on bulk liquid density should be expected in real MC simulations in Cassandra. To quantify this possible source of computational uncertainty, several test runs of both fully classical and nested DFTB MC simulations of the bulk liquid were performed, each constraining molecular bond-lengths at distinct data sets from Table S4.

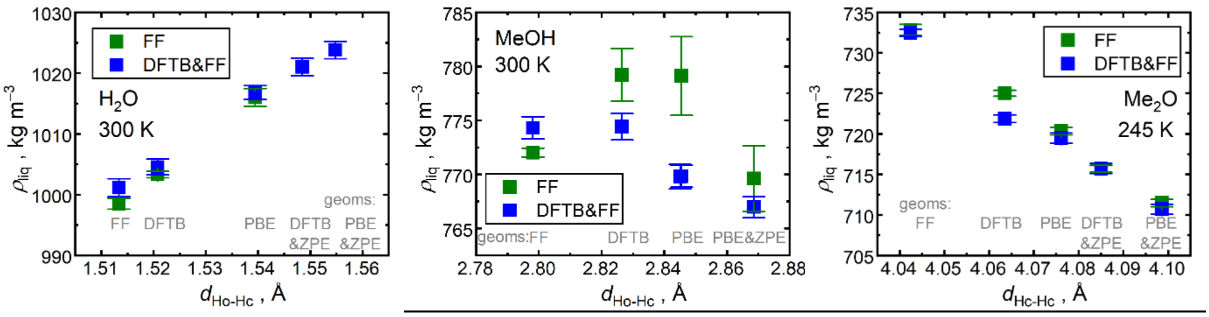

Figure S6. Bulk phase densities resulting from MC simulations either fully classical at the force field (FF) level only, or at the nested DFTB level of theory. Monomer bond lengths were constrained in all FF MC sequences generated in Cassandra at respective values given in Table S4 or in Table S5. Independent variable then corresponds to the largest distance between any two atoms in a molecule.

Figure S6 illustrates the variation in equilibrium bulk liquid densities due to the distinct constrained bond lengths in monomers. Despite the simulations mimicked otherwise identical conditions, differences in the observed densities far exceed the statistical sampling uncertainties, being comparable in magnitude to the differences in the very bond lengths among individual monomer models. Elongation of the monomer bond length by 2.3% (comparing the force field geometry and the PBE model including anharmonic ZPE) for water resulted in the same percentage increase of its bulk density. On the other hand, a molecular elongation by 1.5% for dimethyl ether resulted in 3% density decrease. This qualitatively different response can be interpreted in terms of the molecular interactions that the individual target molecules form in the bulk. Density of the dispersion-bound dimethyl ether naturally scales with the molecular size, whereas hydrogen-bonded water molecules violate this trend. Since hydrogen-bonding strength is negatively correlated to the length of the covalent O–H bonds donating the hydrogen atoms,[20, 59] longer water molecules then form stronger hydrogen bonds that corresponds to overbinding of the bulk phase and an artificial overestimation of its density.

To emphasize the first-principles nature of the developed nested MC simulation methodology, the QM MC simulation itself should be preceded by an analysis of the bond lengths in monomer molecules at the same QM level of theory for the sake of consistency of theories used for sampling the monomer structure and its non-covalent interactions. Throughout the production QM MC simulations, monomer geometries were always used consistently as presampled by the given computational method, including the ZPE effects on the bond lengths.

S2. Reference data

Low-uncertainty thermodynamic reference data were derived from existing literature to enable fair and stringent validation of the computational models and their results. For this purpose, we preferred using correlation models or fundamental equation of states that were developed on the basis of critically assessed experimental data on various thermodynamic properties and that enable to derive density as a function of temperature. Furthermore, vaporization enthalpies $\Delta_{vap}H$ were derived from the Clausius-Clapeyron equation:

$$\Delta_{vap}H = \Delta z\, RT^2 \left(\frac{\partial \ln p_s}{\partial T} \right), \qquad \text{(S10)}$$

where p_s represent saturated vapor pressures and $\Delta z = z_{gas} - z_{liq}$ stands for the difference between the compressibility factors of the gas and liquid phases. Note that the Δz term proves to be important as the non-ideal gas-phase behavior manifests itself at vapor pressures close to the normal boiling points of some of the target materials. Experimental data on both liquid and vapor phases along the saturation curve are thus needed to evaluate $\Delta_{vap}H$ according to equation S10. Reference data used throughout this work for benchmarking purposes are summarized in Table S6.

For water, a fundamental equation of state[60] was used to derive its liquid-phase gas-phase densities and vaporization enthalpy including the correction due to non-ideal gas-phase behavior.

For methanol, a fundamental equation of state[61] was used to derive its liquid-phase and gas-phase densities. Vaporization enthalpy was derived from vapor pressure data correlated in ref. [62].

For dimethyl ether, liquid-phase densities were correlated with a cubic polynomial from experimental data contained in refs.[63, 64] that were in a very good mutual agreement. Vaporization enthalpies were derived from vapor pressure data description using the Wagner equation from ref.[63] Peng-Robinson equation of state[65] (parametrized with respect to the critical coordinates from ref. [63]) was used to estimate the correction to the vaporization enthalpy due to non-ideal gas-phase behavior.

Table S6

Summary of reference data on liquid-phase densities (ρ_{liq}) at ambient pressure and vaporization enthalpies ($\Delta_{vap}H$) at selected temperatures that were used in this work for benchmarking of results of the computational models.

	Water		
T, K	300	320	340
ρ_{liq}, kg·m^{-3}	996.566 ± 0.001	989.428 ± 0.001	979.536 ± 0.001
$\Delta_{vap}H$, kJ·mol^{-1}	43.91 ± 0.01	43.05 ± 0.01	42.17 ± 0.01
	Methanol		
T, K	260	280	300
ρ_{liq}, kg·m^{-3}	822.46 ± 0.83	803.66 ± 0.81	784.86 ± 0.79
$\Delta_{vap}H$, kJ·mol^{-1}	39.23 ± 0.12	38.40 ± 0.12	37.42 ± 0.12
	Dimethyl ether		
T, K	205	225	245
ρ_{liq}, kg·m^{-3}	792.19 ± 0.73	766.43 ± 0.77	739.76 ± 0.74
$\Delta_{vap}H$, kJ·mol^{-1}	23.56 ± 0.16	22.70 ± 0.12	21.78 ± 0.11

S3. Detailed discussion of simulation results

S3.1. Examples of raw FrAMonC simulation data

To verify a proper functionality of the implemented nested QM MC simulations, this section provides representative examples of raw and intermediate simulation data assembled throughout the FrAMonC simulations performed at the MP2:PBE level of theory. As shown above, the keystone in the acceptance criterion of nested QM MC simulations is the energy difference evaluated for the final configuration of the inner nested MC sequence with the classical FF and with a QM method. Figure S7 illustrates the relationship between the FF and QM energies observed within a portion of FrAMonC simulations of bulk water at 300 K where the composite fragment-based MP2:DFTB model was used to express the QM energy of the bulk. It clearly demonstrates a strong positive correlation between the bulk liquid energies obtained by both FF and QM methods. Discrepancies between both methods, however, still leave potential for some broadening of this distribution. Following the idea of the nested MC simulations, the algorithm then preferentially rejects configurations where the FF model underestimates repulsive effects, i. e. it artificially overbinds the bulk cohesion in such configurations. That behavior can be glimpsed from the systematic shift of the cloud of red points (configurations rejected after QM assessment) with respect to the clouds of blue points (configurations accepted in the QM assessment). Since the QM assessment has naturally a stochastic character, there are overlaps of both the cloud types.

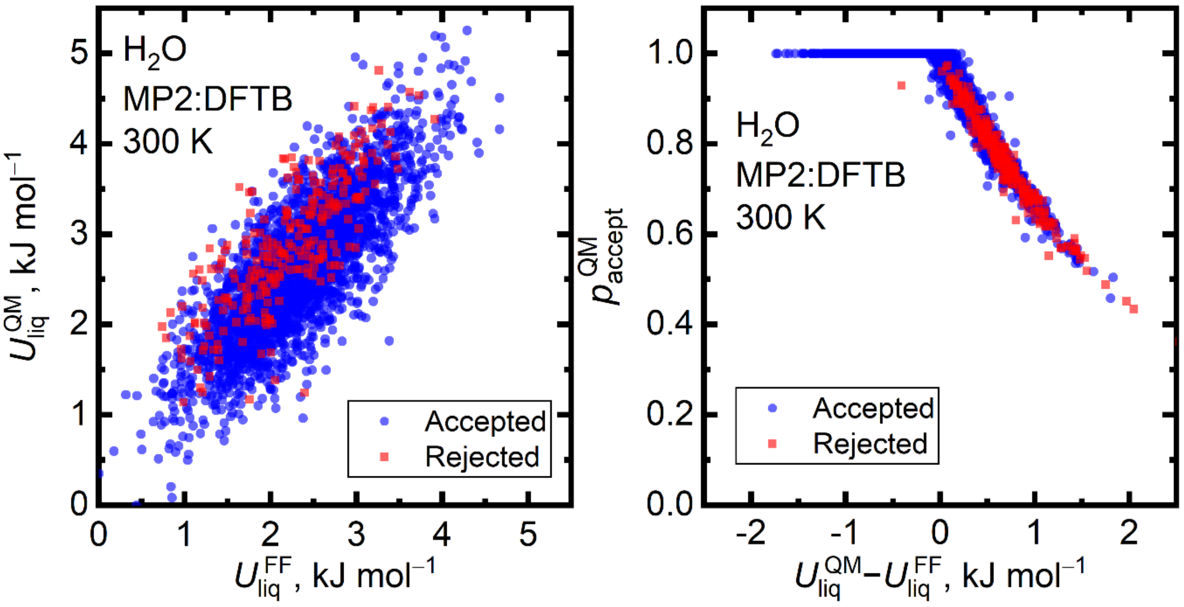

Figure S7. Nested FrAMonC sampling of bulk water at 300 K: Left – comparison of total energies observed within FrAMonC simulations of with a classical force field and with fragment-based composite MP2:DFTB approach to model the QM energy; Right – illustration of the probability of acceptance within the QM assessment as a function of the bulk energy difference observed between the FF and QM models.

Just for practical reasons, the zero energy level in the left part of Figure S7 corresponds to configuration with the lowest total energy observed within the given portion of the MC simulation. Furthermore, Figure S7 also illustrates the probability of acceptance within individual QM assessments, indicating that overbinding of the bulk by the QM model leads nearly exclusively to accepting the configurations, whereas overbinding by the MM model translates to an exponential decay of the probability of acceptance. Note that the width of those observed probability distributions can be attributed to the scaling of the temperature in the inner nested MC sequences. Also note that the right-most tails of data points gradually fade away as observing such large differences (over 3 kJ·mol^{-1}) between the instantaneous bulk energies are rather unlikely when reasonably compatible QM and FF models are used for the inner and outer MC sampling.

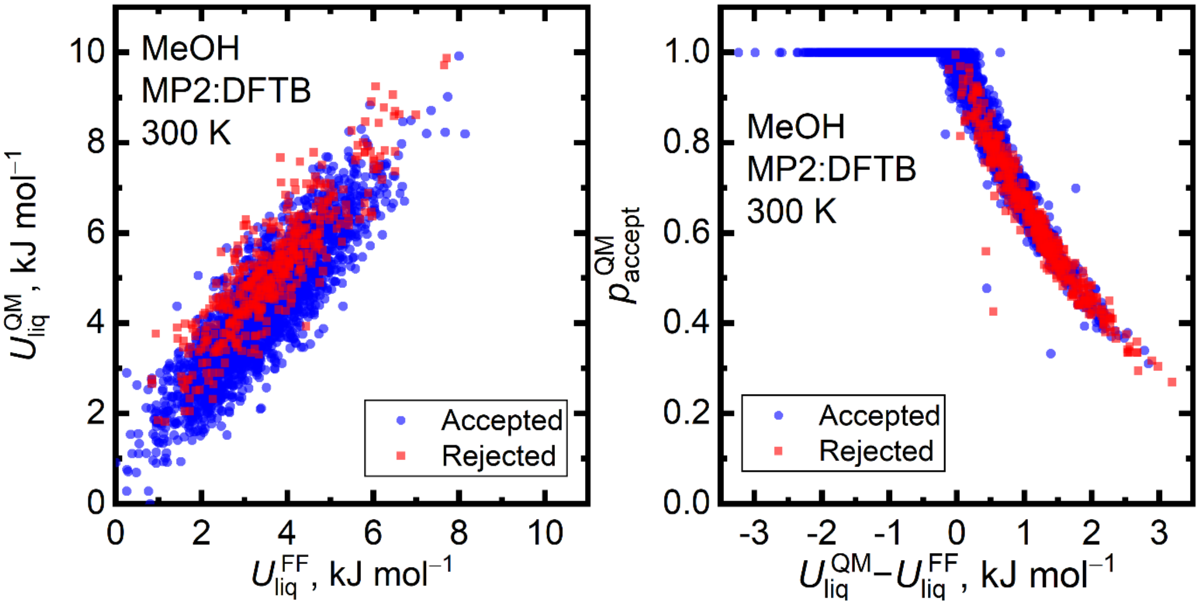

Figure S8. Nested FrAMonC sampling of bulk methanol at 300 K: Left – comparison of total energies observed within FrAMonC simulations of with a classical force field and with fragment-based composite MP2:DFTB approach to model the QM energy; Right – illustration of the probability of acceptance within the QM assessment as a function of the bulk energy difference observed between the FF and QM models.

A similar statistics is shown also for the FrAMonC simulations of bulk methanol at 300 K in Figure S8. More complex molecular shape of methanol, already allowing for some conformation degrees of freedom to manifest in the bulk liquid, propagates to a wider distribution of bulk energy points when compared to water simulations. Otherwise, the qualitative behavior of the QM assessment and related acceptance rates are comparable – the above diagonal points of the energy comparison in the left part of Figure S8 and the right-most branch of data points in the right part of Figure S8 correspond proportionally to rejected configurations. Finally, a similar analysis is presented for FrAMonC simulations of bulk dimethyl ether at 245 K in Figure S9.

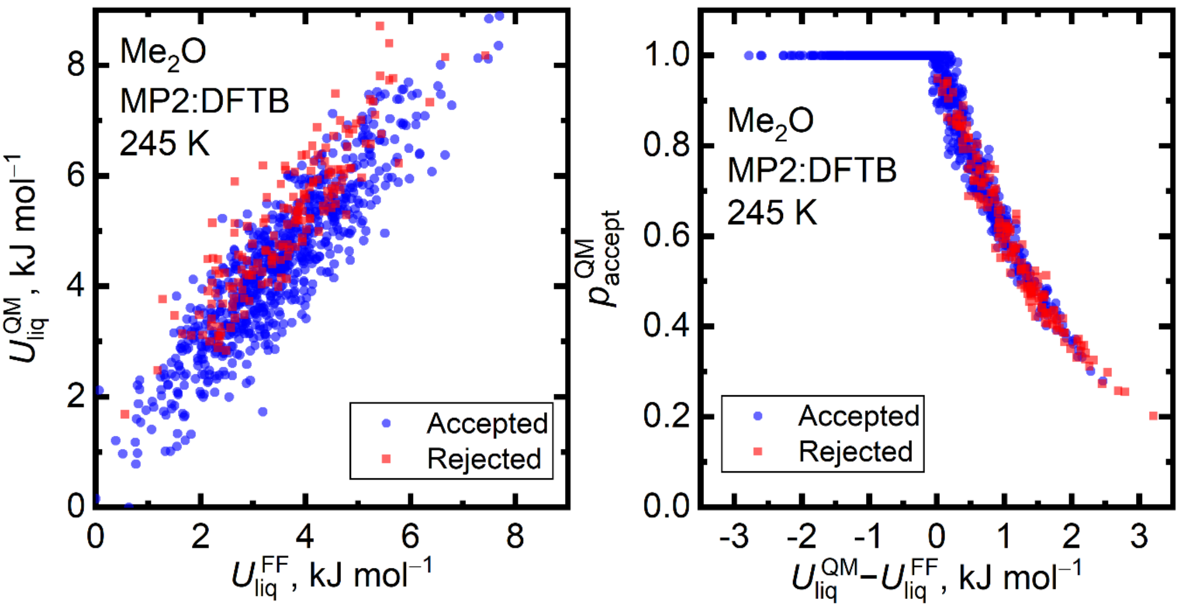

Figure S9. Nested FrAMonC sampling of bulk dimethyl ether at 245 K: Left – comparison of total energies observed within FrAMonC simulations of with a classical force field and with fragment-based composite MP2:DFTB approach to model the QM energy; Right – illustration of the probability of acceptance within the QM assessment as a function of the bulk energy difference observed between the FF and QM models.

A more detailed inspection of the nested FrAMonC workflow can be made from plotting the energies of the inner nested MC sequences, treated with FF, and the QM energies observed upon the energy assessment of the final configuration of each inner nested MC sequence, again paying attention to color-coding with respect of the verdict of the QM assessment of individual configurations. Such an analysis is depicted in Figure S10 for selected short portions of the FrAMonC simulations for all three materials employing the MP2:PBE level of theory for the QM treatment. In those plots in Figure S10, the lower box always corresponds to the classical FF treatment. Note that only every twentieth point generated in the inner nested MC sequences is shown for clarity of the plot. Acceptation or rejection of the inner nested MC sequences appears as completely random from the plot of the MM energies only as the terminal configurations of the inner nested sequences do not exhibit any visible bias towards higher energies. Furthermore, FF energies of those terminal configurations (separated always by 270 MC sweeps) still exhibit a certain level of mutual correlations. On the other hand, when one considers the QM energies evaluated for the identical configurations at the end of the inner MC loops (that are always contained in the upper boxes in Figure S10) there is an obvious bias of the rejected configurations towards higher QM energies and also individual energy points are clearly less correlated.

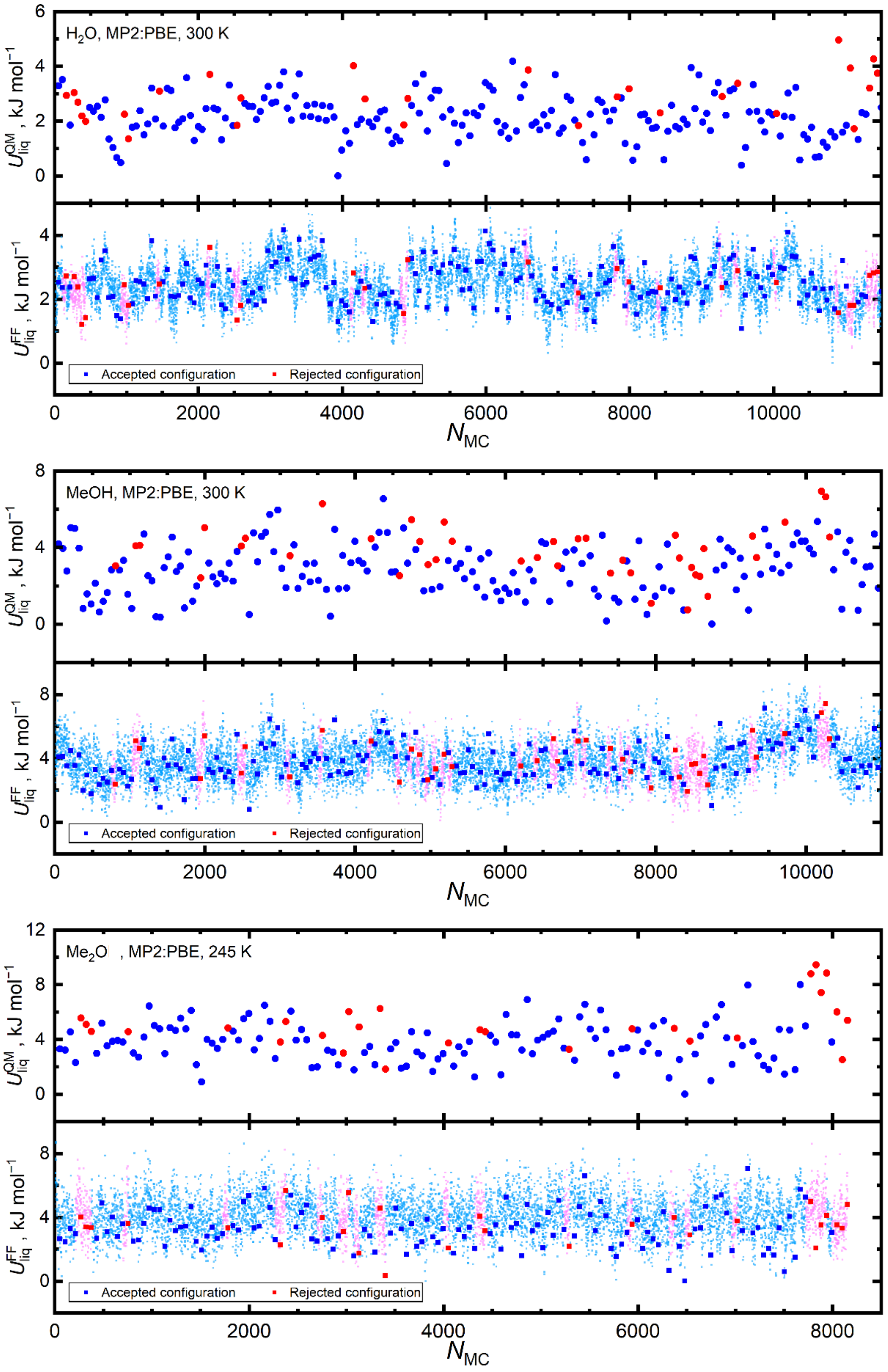

Figure S10. Raw total energies of bulk liquids assembled in parts of the MP2:PBE FrAMonC simulations of the target materials. Bottom frames correspond to the output of inner nested classical MC sequences ($U_{\mathrm{liq}}^{\mathrm{FF}}$, individual sampled configurations within the nested MC sequences given with thin points, and energies of the final configurations from each nested MC sequence given with thick squares). Top frames correspond to the output of the QM assessment of the individual final configurations ($U_{\mathrm{liq}}^{\mathrm{QM}}$).

S3.2. Detailed results on liquid-phase density

This section lists numerical values of simulated liquid-phase densities and compares them with the experimental reference values in Table S7. Convergence towards the simulated ensemble average density values and details on the structure of the simulated bulk liquids in terms of the radial distribution functions are given afterwards.

Table S7
Comparison of calculated[a] liquid-phase densities (kg·m^{-3}) of target molecular liquids at ambient pressures and selected temperatures with literature reference values.

	Water·		
Temperature, K	300	320	340
Experiment[b]	996.6	989.4	979.5
Force field	998.2	977.9	963.8
DFTB	1024.4	1004.8	989.6
PBE	1019.5	1010.5	995.5
MP2:DFTB	1014.3	998.5	979.7
CC:DFTB	1013.0	997.8	980.7
MP2:PBE	1010.0	994.9	979.1
CC:PBE	*1008.7*	*994.2*	*980.1*
	Methanol		
Temperature, K	260	280	300
Experiment[c]	822.5	803.7	784.9
Force field	817.3	794.6	773.0
DFTB	814.6	794.3	770.4
PBE	807.9	789.5	767.2
MP2:DFTB	813.7	793.0	774.4
CC:DFTB	814.4	797.2	775.0
MP2:PBE	815.0	795.8	775.4
CC:PBE	*815.8*	*800.0*	*776.0*
	Dimethyl ether		
Temperature, K	205	225	245
Experiment[c]	792.2	766.5	739.8
Force field	795.7	765.2	732.8
DFTB	775.3	746.3	715.7
PBE	771.8	741.4	711.1
MP2:DFTB	779.9	752.4	720.1
CC:DFTB	779.3	750.6	718.1
MP2:PBE	779.6	752.4	718.6
CC:PBE	*779.0*	*750.7*	*716.6*

[a] Statistical sampling uncertainty is 1.5 kg·m^{-3} or less; [b] Experimental uncertainty is 0.001 kg·m^{-3}; [c] Experimental uncertainty is 0.8 kg·m^{-3}.

A summary of the computational performance of individual considered QM MC and FrAMonC simulations in terms of the root-mean-squared errors (RMSE, absolute measure defined in equation S11) and root-mean squared relative deviations (RMSRD, defined in equation S12) of bulk liquid densities is given in Table S8.

$$\mathrm{RMSE} = \sqrt{\frac{1}{n}\sum_{i=1}^{n}\left(X_i^{\mathrm{calc}} - X^{\mathrm{exp}}\right)} \qquad \text{(S11)}$$

$$\mathrm{RMSRD} = \sqrt{\frac{1}{n}\sum_{i=1}^{n}\left(\frac{X_i^{\mathrm{calc}}}{X^{\mathrm{exp}}}-1\right)} \tag{S11}$$

Note that both the given RMSE and RMSRD values were computed with respect to experimental data and averaged over the entire set of three temperatures considered for each of the three target materials. In the literature, there are reports of earlier DFT MD simulations of liquid methanol with common functionals, such as PBE, BLYP, and vdW-DF, yielding errors of the methanol density at ambient-conditions ranging from −8 to +23%, depending on the functional.[66] In this context, current FrAMonC simulations exhibit a superior performance over DFT MD simulations.

Table S8
Statistics about the accuracy of bulk liquid densities simulated using individual methods from experimental reference data. Values are shown both at the relative and absolute (in kg·m^{-3}) scales.

Model	RMSRD	RMSE
Force field	1.00%	8.77
DFTB	2.08%	17.45
PBE	2.50%	20.59
MP2:DFTB	1.55%	12.62
CC:DFTB	1.57%	12.59
MP2:PBE	1.47%	11.65
CC:PBE	*1.51%*	*11.85*

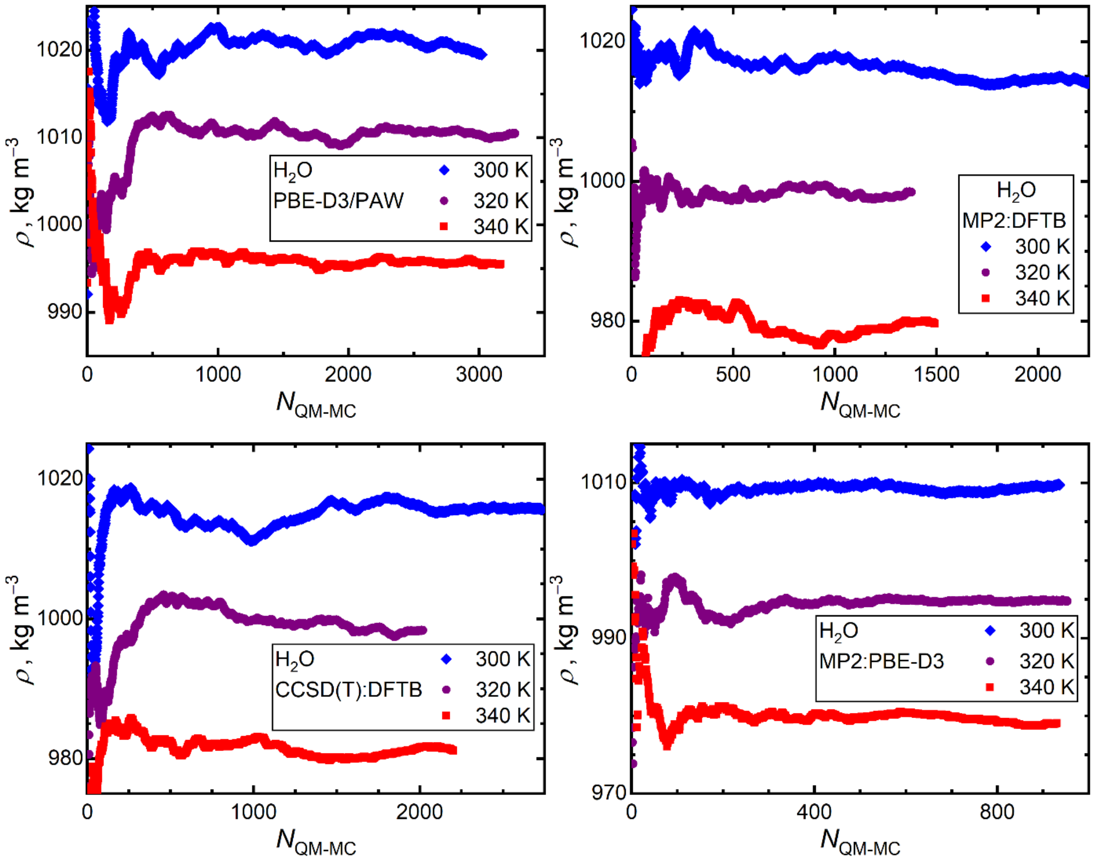

Figure S11. Running averages of liquid water densities as a function of the numbers of accepted configurations $N_{\text{QM-MC}}$ assembled during various QM MC simulations.

Similarly to the above shown analysis of the running averages of simulated observables and related statistical sampling uncertainties from the validation stage, using FF or DFTB levels of theory within the MC simulations, Figure S11 to S13 depict the running averages of the bulk liquid densities obtained from production QM MC simulations, employing PBE/PAW and FrAMonC sampling (with MP2:DFTB, CC:DFTB or CC:PBE methods) at three selected temperatures. Figure S11 to S13 also indicate the total numbers of QM-MC accepted configurations, exceeding 1500, 800 and 500 for most cases of such simulations for water, methanol and dimethyl ether, respectively.

It can be seen that the running average of the density dramatically oscillates when only less than 200 accepted configurations are used for the averaging. Such a modest computational setup, relying on a too limited number of configurations, can be proven as useless even for qualitative interpretations although high-level electron-structure theories may be involved. Not even the correct hierarchy of densities at individual temperatures with respect to the expected thermal expansion of the bulk would be captured in such a case.

Including over 500 accepted configurations for the ensemble averaging (over 1200 for water) then minimizes any fluctuations of the average simulated densities with respect to generating additional configurations. For such sizes of the simulated configuration ensembles, simulation sampling uncertainty of bulk densities can be then generally converged within the targeted 1.5 kg·m^{-3} threshold also for the considered QM MC simulation setups, regardless of the combination of high-level and medium-level QM methods within the FrAMonC scheme. General trends of the running averages and their convergence rate seem to be insensitive to the actual employed QM level of theory. Using at least 300 configurations seems to be sufficient to qualitatively capture the correct density hierarchy at individual temperatures given by the thermal expansion.

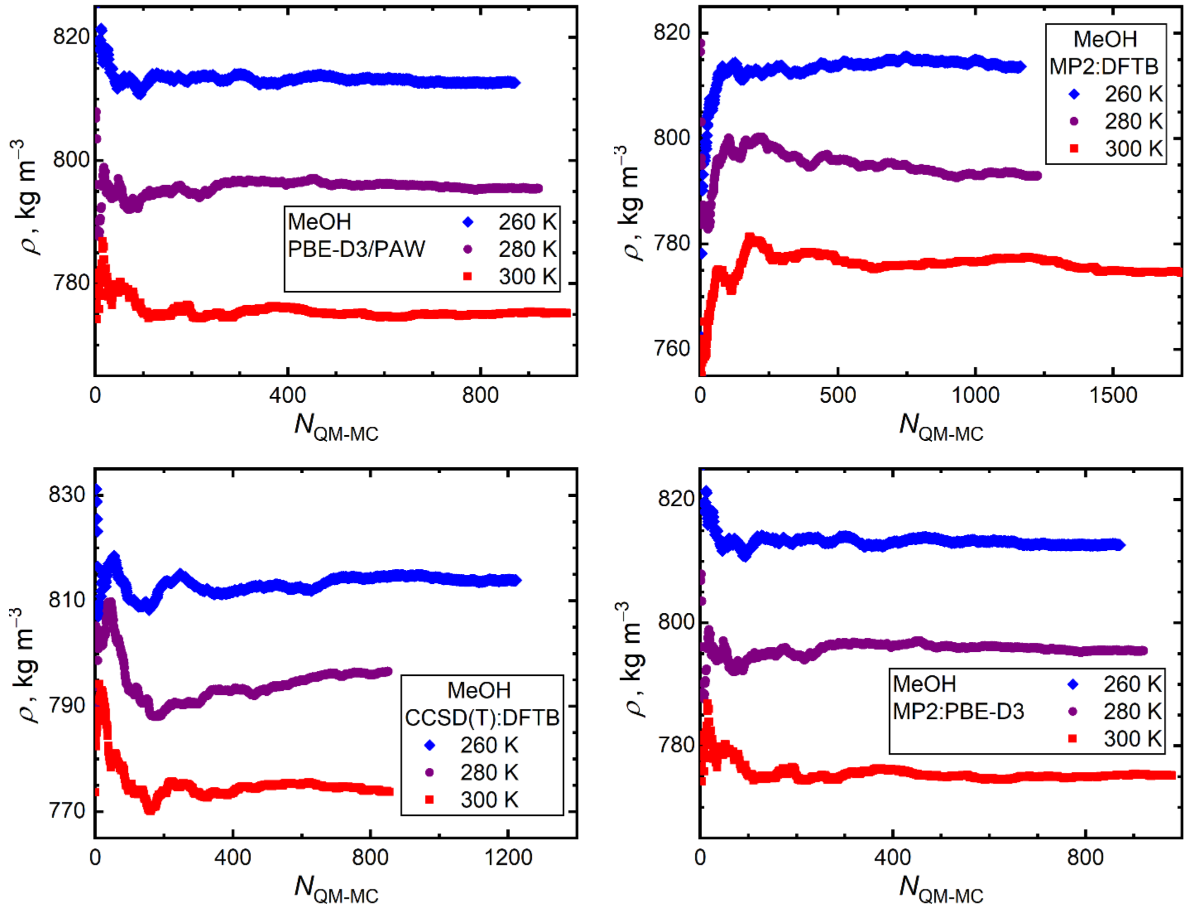

Figure S12. Running averages of liquid methanol densities as a function of the numbers of accepted configurations $N_{\text{QM-MC}}$ assembled during various QM MC simulations.

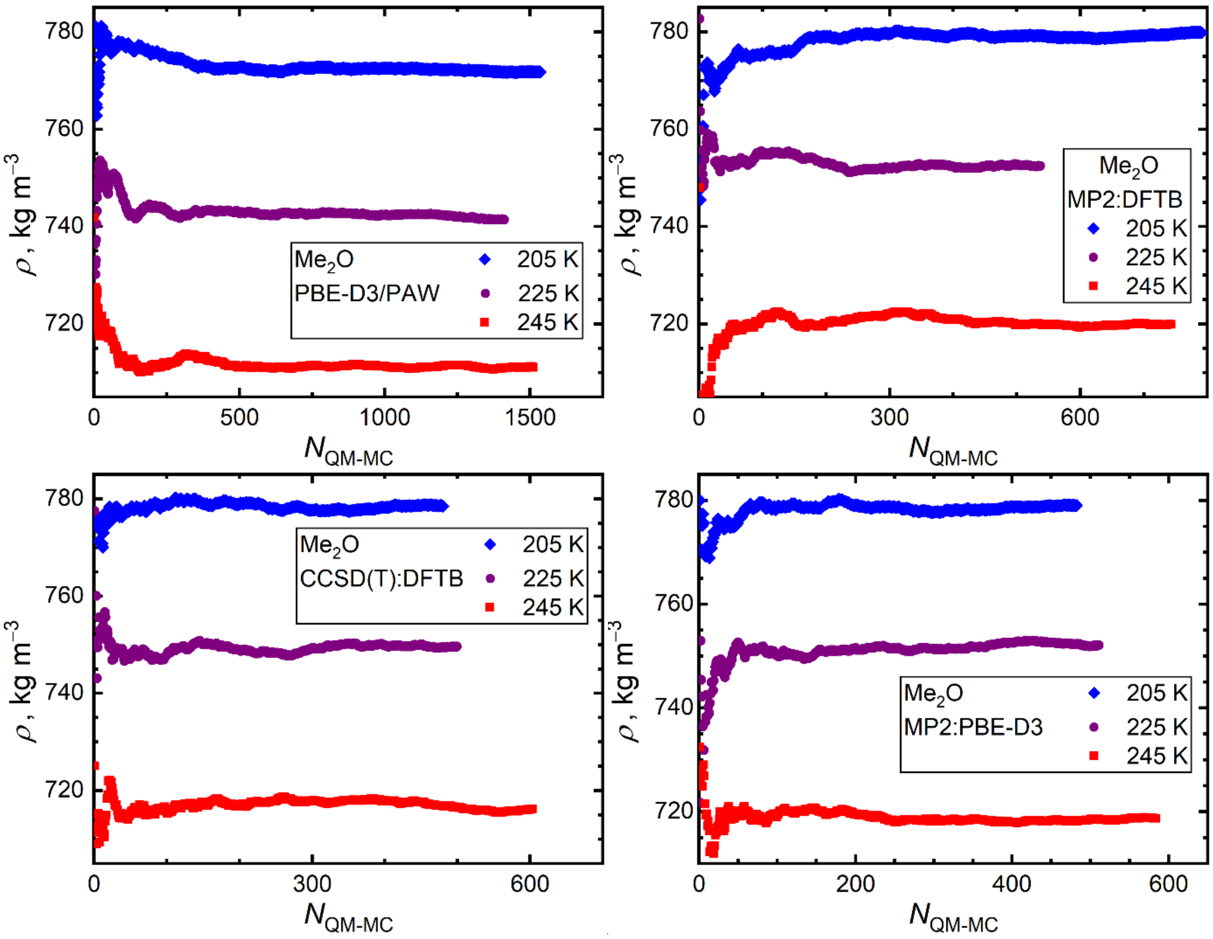

Figure S13. Running averages of liquid dimethyl ether densities as a function of the numbers of accepted configurations $N_{QM\text{-}MC}$ assembled during various QM MC simulations.

In case of using the most expensive FrAMonC computational setup, relying either on the DLPNO-CCSD(T) as the high-level method for treatment of pair interactions or on PBE-D3(BJ) as the medium-level method, generation of a single continuous MC sequence spanning over 1000 QM assessed configurations (granting a sufficiently statistical sampling uncertainty) can become too costly. At the same time, relying on a single shorter continuous MC sequence would lead to inacceptable sampling uncertainty increase. An optimum solution to this is to generate multiple independent FrAMonC sequences in parallel, to compute the statistical averages of the targeted observables for each such simulation separately, and finally to compute the resulting observable value as an uncertainty-weighted average over the independent runs. In such as case, the uncertainty of such as averaged value needs to take both the scatter of results of the individual FrAMonC runs as well as the sampling uncertainties of each of those runs.[54]

S3.3. Detailed results on liquid-phase radial distribution functions

To investigate structural details about molecular packing in the bulk liquids, radial distribution functions $g(r)$ for important atomic contacts were calculated from the ensembles of MC configurations that were

accepted in the outer, QM-treated, MC loop. The aim is laid again on comparison of the results of individual computational models with experimental data. At this point, relatively small size of the simulation boxes spanning at least 12 Å enabled us to sample *g*(*r*) only over atomic separation no longer than 6 Å. Considering the small size and compactness of the target molecules, however, such a cut-off distance suffices to cover the first and second solvation shells in the bulk, which represent the most important *g*(*r*) features anyway. We compare our results simulated at 300 K and 100 kPa with experimental coordinates of the first two *g*(*r*) peaks that were culled from literature for water[67, 68] and methanol.[69, 70] In both cases, *g*(*r*) corresponding to non-covalent O…O contacts are compared. For dimethyl ether, we are not aware of any experimental *g*(*r*) data interpreting the structure of its bulk liquid.

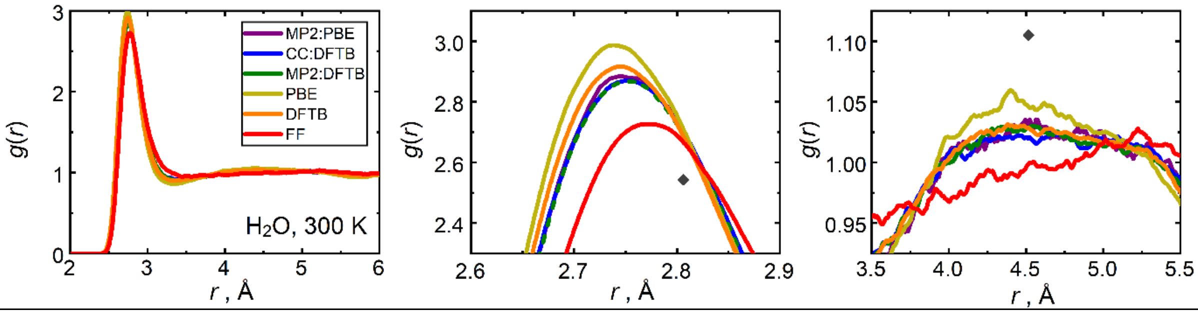

Figure S14. Radial distribution functions *g*(*r*) for O…O contacts in liquid water at 300 K and 100 kPa as obtained from current MC simulations. Left box displays the entire sampled interval while the middle and right boxes zoom on the first and second *g*(*r*) peaks, respectively. Gray diamonds correspond to experimental coordinates of the first and second *g*(*r*) peaks taken from refs.[67, 68].

Detailed *g*(*r*) results for water are depicted in Figure S14. All computational MC models yield very similar *g*(*r*) profiles with a sharp peak due to the first O…O coordination shell followed by a very flat and rather indistinct maximum due to the second coordination shell. A closer inspection of the first *g*(*r*) peak in Figure S14 reveals that all computational models underestimate the distance coordinate of the first peak and overestimate its amplitude when compared to experiment. In this case, the closest agreement was reached by the empiric force field which is not that surprising since its van der Waals parameters have origins in parametrization to experimental properties of bulk liquid. First-peak coordinates obtained from PBE simulations are then the most outlying one, exhibiting a twice as large error the FF model. Importantly, adopting the ab initio corrections within the FrAMonC model leads to improvements of the *g*(*r*) as both MP2 and CCSD(T) models yield lower errors than DFTB does. Within our FrAMonC simulations, the CCSD(T) model underestimates the position of the first peak by 0.05 Å (or 1.8%), and it overestimates its amplitude by 13%.

Focusing on the second O…O coordination shell in bulk water, Figure S14 reveals that the FF model is not able to reproduce the diffuse shape of the second *g*(*r*) peak correctly and it overestimates its position by more than 1.2 Å. On the other hand, all our QM based MC simulations predict the distance coordinate very close to its experimental location, this time somewhat underestimating its otherwise very weak amplitude. Surprisingly, PBE is now at the closest from the experiment. Within our FrAMonC

simulations, the CCSD(T) model misses the experimental coordinates of the second $g(r)$ peak by 0.06 Å (≈1.3%) in terms of its position and by 7.5% in terms of its amplitude.

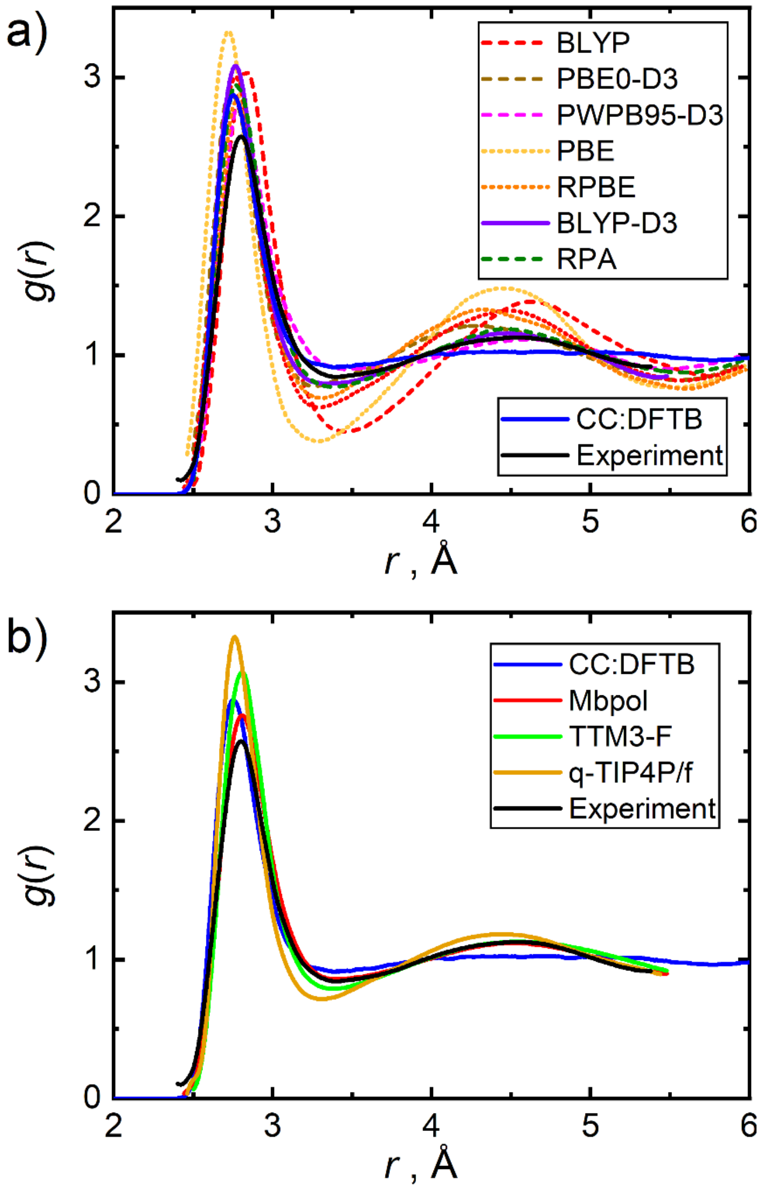

Figure S15. Comparison of O…O radial distribution function $g(r)$ in liquid water near 298 K and 100 K calculated from first principles in the recent literature and of experimental data.[68] Color-coding with respect to the underlying levels of theory, legend for part a): solid blue line – this work; solid violet line – ref.[71]; dashed lines – ref.[72]; dotted lines – ref. [73], legend for part b): solid blue line – this work; other solid lines – ref. [71].

Analyzing literature results of $g(r)$ calculated from first principles for liquid water at ambient conditions, Figure S15 depicts that our current CC:DFTB results agree with the experimental $g(r)$ data[68] closer than most reported results of DFT functionals performed either with GGA or hybrid functionals. Our CC:DFTB results are then relatively close to the results of AIMD simulations performed at RPA or PWPB95-D3 levels of theory. This level of (dis)agreement of individual datasets is in particular visible

for the coordinates and amplitudes of the first $g(r)$ minimum and the second $g(r)$ maximum that are relatively well captured by our CC:DFTB FrAMonC simulations, as well as by the RPA or PWPB95-D3 AIMD simulations reported in the literature.[72, 74] Lower-tier DFT calculations, often even lacking any dispersion corrections then exhibit a massive scatter in its radial coordinate, and significantly overstate the depth of the first minimum and the height of the second maximum,[73] being in a stark contrast to experimental data.

Figure S15 also compares current FrAMonC results on $g(r)$ for liquid water with results of simulations using selected classical potentials or ab initio machine-learned MBpol potential that were presented in the literature.[71] While the current CC:DFTB $g(r)$ results agree at the closest with both MBpol and experimental coordinates and amplitudes of the first $g(r)$ maximum and maximum, the amplitude of the second $g(r)$ maximum is somewhat underestimated by the CC:DFTB FrAMonC model, unlike the other considered data sets.

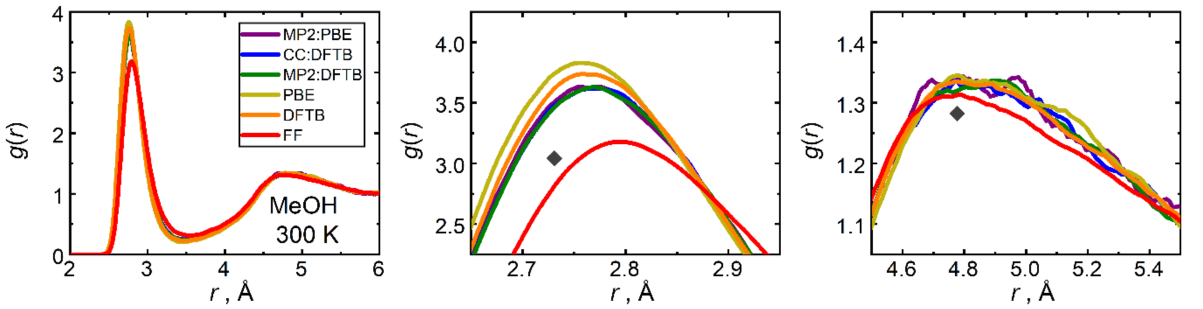

Figure S16. Radial distribution functions $g(r)$ for O…O contacts in liquid methanol at 300 K and 100 kPa as obtained from current MC simulations. Left box displays the entire sampled interval while the middle and right boxes zoom on the first and second $g(r)$ peaks, respectively. Gray diamonds correspond to experimental coordinates of the first and second $g(r)$ peaks taken from refs.[69, 70].

Detailed $g(r)$ results for methanol are depicted in Figure S16. Its O…O contacts can be described with $g(r)$ functions similar to those of water, this time with a more pronounced gap between the first two coordination shells and with a more distinct second peak. A closer inspection of the first $g(r)$ peak in Figure S16 shows that the FF model captures the correct peak amplitude but it overestimates the distance coordinate. All QM based MC simulations then predict the distance coordinate closer to the experiment, but somewhat overestimate the peak amplitude. A similar pattern among individual QM models repeats here with PBE yielding the most outlying amplitudes and both MP2 and CCSD(T) correcting the signal of DFTB when coupled in the FrAMonC scheme. Our CCSD(T) model misses the actual first peak coordinates by 0.04 Å (≈1.5%) and by 19% in terms of the distance and amplitude, respectively.

All computational models then agree very closely on the shape and position of the second $g(r)$ peak for methanol and their agreement with the experiment is very good in this case. Our CCSD(T) based FrAMonC simulations differ from the experiment by less than 0.01 Å (≈0.04%) and 2.9% in terms of the position and amplitude of the second peak.

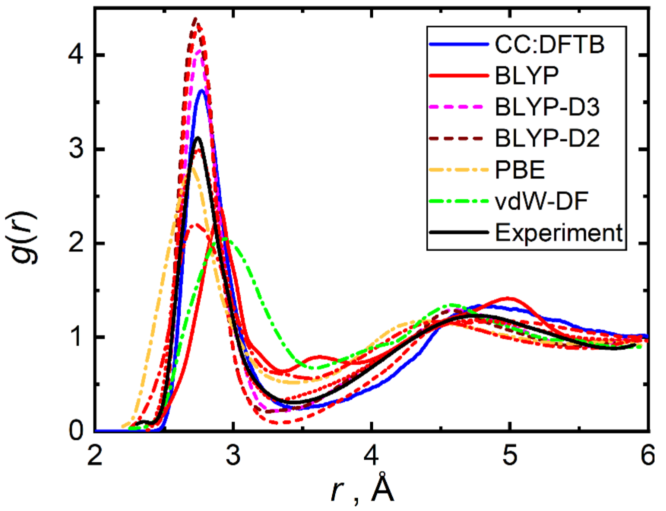

Figure S17. Comparison of O…O radial distribution function $g(r)$ in liquid methanol near 293–298 K and 100 kPa calculated from first principles in the recent literature and of experimental data .[69] Legend: solid blue line – this work; solid red line – ref.[75]; dashed lines – ref.[76]; dash-dotted lines – ref.[66]; dotted lines – ref. [77].

A comparison of current FrAMonC results on $g(r)$ for liquid methanol with experimental data[69, 70] and literature results of relevant ab initio simulations is presented in Figure S17. Clearly, current CC:DFTB $g(r)$ data set exhibits one of the closest agreements with the experiment in terms of both the coordinates and amplitudes of all of the first maximum, first minimum, and second maximum. Literature results obtained from earlier AIMD simulations, relying mostly on dispersion-uncorrected GGA DFT models exhibit considerable scatter, especially for the amplitude of the first $g(r)$ maximum.[75-77] Notably, even different AIMD data sets on the O…O $g(r)$ functions, reported in the literature to be all computed with the BLYP functional, are severely scattered. That suggests that detailed computational setup of the AIMD simulations, such as box size, pseudopotentials, basis-set size, reciprocal space sampling, etc. may affect the resulting $g(r)$ importantly.

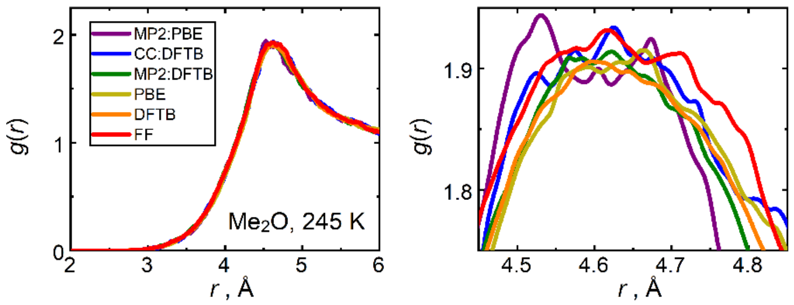

Figure S18. Radial distribution functions $g(r)$ for O…O contacts in liquid dimethyl ether at 245 K and 100 kPa as obtained from current MC simulations. Left box displays the entire sampled interval while the right box zooms on the first (r) peaks.

Finally, detailed $g(r)$ results for dimethyl ether are depicted in Figure S18. Due to the lack of any site-specific strong cohesive interactions, such as hydrogen bonding, $g(r)$ corresponding to its O…O contacts does not exhibit a sharp peak below 2.8 Å that was characteristic for the other liquids. There is only one important, yet lower and broader, $g(r)$ peak within the 6 Å cut-off, agreed upon closely by all the computational models. Among the individual models, scatter in the predicted position and amplitude of the first peak is as low as 0.06 Å and 0.02, respectively.

S3.4. Detailed results on thermal expansion

Being equipped with trends of bulk liquid densities with respect to temperature, one can evaluate the coefficients of thermal expansion of the liquid α. Table S9 lists the α values derived from all simulations for all three considered liquids and compares them with the experimental values over relevant temperature ranges.

Table S9
Comparison of calculated[a] coefficients of thermal expansion (α, in K^{-1}) of target molecular liquids at ambient pressures and within selected temperatures ranges with literature reference values. Root-mean-squared relative deviations (RMSRD) of data sets on α computed using individual methods are also listed.

Material	Water	Methanol	Dimethyl ether	RMSRD
Temperature range	300–340 K	260–300 K	205–245 K	
Experiment[b]	4.31×10^{-4}	1.17×10^{-3}	1.71×10^{-3}	–
Force field	8.74×10^{-4}	1.39×10^{-3}	2.06×10^{-3}	61.4%
DFTB	8.63×10^{-4}	1.40×10^{-3}	2.01×10^{-3}	59.7%
PBE	5.97×10^{-4}	1.30×10^{-3}	2.05×10^{-3}	23.0%
MP2:DFTB	8.70×10^{-4}	1.24×10^{-3}	2.00×10^{-3}	58.8%
CC:DFTB	8.11×10^{-4}	1.25×10^{-3}	2.05×10^{-3}	52.2%
MP2:PBE	7.77×10^{-4}	1.25×10^{-3}	2.05×10^{-3}	47.8%
CC:PBE	7.18×10^{-4}	1.26×10^{-3}	2.10×10^{-3}	*40.6%*

S3.5. Detailed results on vaporization enthalpies

This section lists numerical values of simulated vaporization enthalpies and compares them with the experimental reference values in Table S10. Convergence towards the simulated ensemble average density values and details on individual simulated molecular interactions taking place in the bulk liquid are given afterwards.

Table S10
Comparison of calculated vaporization enthalpies (kJ·mol^{-1}) of target molecular liquids at ambient pressure and selected temperatures with literature reference values.

	Water [a].		
Temperature, K	300	320	340
Experiment[b]	43.91	43.05	42.17
Force field	43.75	42.87	41.97
DFTB	44.02	43.13	42.26
PBE	46.35	45.26	44.31
MP2:DFTB	39.36	38.42	37.62
CC:DFTB	38.08	37.35	36.35
MP2:PBE	37.83	36.97	36.01
CC:PBE	*36.52*	*35.90*	*34.75*

	Methanol [c]		
Temperature, K	260	280	300
Experiment[d]	39.2	38.4	37.4
Force field	38.41	37.34	36.15
DFTB	31.00	30.31	28.81
PBE	41.27	38.57	38.04
MP2:DFTB	33.38	32.37	31.20
CC:DFTB	32.85	32.20	30.85
MP2:PBE	34.01	32.89	31.90
CC:PBE	*33.47*	*32.72*	*31.55*
	Dimethyl ether [c]		
Temperature, K	205	225	245
Experiment[e]	23.6	22.7	21.8
Force field	24.41	22.76	20.47
DFTB	17.29	16.05	14.99
PBE	21.56	20.07	19.31
MP2:DFTB	16.13	15.23	14.25
CC:DFTB	18.31	16.60	16.36
MP2:PBE	14.84	14.18	13.34
CC:PBE	*17.02*	*15.55*	*15.45*

[a] Statistical sampling uncertainty is 0.2 kJ·mol^{-1} or less; [b] Experimental uncertainty is 0.01 kJ·mol^{-3}; [c] Statistical sampling uncertainty is 0.5 kJ·mol^{-1} or less; [d] Experimental uncertainty is 0.1 kJ·mol^{-1}; [e] Experimental uncertainty is 0.2 kJ·mol^{-1}.

A summary of the computational performance of individual considered QM MC and FrAMonC simulations in terms of both the RMSE and RMSRD of vaporization enthalpies is given in Table S11. Note that those values were computed with respect to experimental data and averaged over the entire set of three temperatures considered for each of the three target materials.

Table S11
Statistics about the accuracy of vaporization enthalpies simulated using individual methods from experimental reference data. Values are shown both at the relative and absolute (in kJ·mol^{-1}) scales

Material	RMSRD	RMSE
Force field	2.86%	0.81
DFTB	20.96%	6.12
PBE	7.06%	2.03
MP2:DFTB	22.01%	6.15
CC:DFTB	18.91%	5.94
MP2:PBE	24.70%	6.83
CC:PBE	*21.50%*	*6.62*

Extending the cut-off distance for an explicit high-level treatment within the MBE energy expression applied on the QM configuration assessment within the FrAMonC simulations would seem as a logical choice how to attempt at improving the numerical accuracy of the predicted enthalpic properties. Our attempts at these improvements performed at the MP2:DFTB level of theory were, nevertheless, not convincing enough to follow this extended computational protocol widely in all production simulations,

as listed in Table S12. For water and methanol, Table S12 shows that there is some sub-kJ/mol variation of $\Delta_{vap}H$ results upon enlarging the dimer cut-off distance from 3 to 6 Å. Although that trend goes in the right direction, its magnitude is too weak to justify the larger computational demands imparted by the larger cut-offs.

Table S12
Results of FrAMonC simulations performed at the MP2:DFTB level of theory with various cut-off distances (d_{cut}, given in Å) for an explicit high-level (i. e. MP2) treatment of the pair interactions. Bulk liquid density data (ρ) given in g·cm^{-3} and vaporization enthalpy data ($\Delta_{vap}H$) given in kJ·mol^{-1}.

Water at 300 K					
d_{cut}	3	4	5	6	Exp.
ρ	1.0163	1.0164	1.0143	1.0155	0.9966
$\Delta_{vap}H$	39.12	39.36	39.49	39.53	43.9
Methanol at 300 K					
d_{cut}	3	4	5	6	Exp.
ρ	0.7736	0.7744	0.7757	0.7740	0.7849
$\Delta_{vap}H$	30.68	31.20	31.46	31.44	37.4
Dimethyl ether at 245 K					
d_{cut}	3	4	5	6	Exp.
ρ	0.7202	0.7201	0.7213	0.7201	0.7398
$\Delta_{vap}H$	13.77	14.25	14.35	14.41	21.8

Being equipped with trends of vaporization enthalpies with respect to temperature, one can evaluate the difference in isobaric heat capacities of vapor and liquid phases, $\Delta_{vap}C_p$. Table S13 lists the $\Delta_{vap}C_p$ values derived from all simulations for all three considered materials and compares them with the experimental values over relevant temperature ranges.

Table S13
Comparison of calculated difference in isobaric heat capacities of vapor and liquid phases ($\Delta_{vap}C_p$, in J·K^{-1}·mol^{-1}) of target molecular liquids at ambient pressures and within selected temperatures ranges with literature reference values. Root-mean-squared relative deviations (RMSRD) of data sets on $\Delta_{vap}C_p$ computed using individual methods are also listed.

Material<br>Temperature range	Water<br>300–340 K	Methanol<br>260–300 K	Dimethyl ether<br>205–245 K	RMSRD
Experiment	−43.6	−45.2	−44.6	–
Force field	−44.7	−56.4	−98.5	71.4%
DFTB	−44.1	−54.9	−57.6	21.0%
PBE	−51.0	−80.8	−56.3	48.9%
MP2:DFTB	−43.5	−54.6	−47.1	12.4%
CC:DFTB	−42.5	−49.9	−48.9	8.4%
MP2:PBE	−45.3	−52.6	−37.5	13.4%
CC:PBE	*−44.4*	*−48.0*	*−39.2*	*7.9%*

MC simulations of isolated monomers, mimicking the ideal-gaseous vapor phase, are generally required to compute the vaporization energy $\Delta_{vap}U$, and thence the vaporization enthalpy. The same computational nested MC setup described above for simulations of the bulk phase was used also to model the vapor phase, relying thus on the Cassandra code for the inner MC sequences where the energies of individual configurations are evaluated from a classical force field. MC sampling of monomers therefore includes variation of conformational degrees of freedom and of bond angles (corresponding rather to lower-frequency molecular vibration modes), whereas bond lengths (corresponding rather to higher-frequency vibrations) are kept rigid.

Figure S19 shows running averages of simulated $\Delta_{vap}U$ values for water using either periodic PBE/PAW calculations or FrAMonC sampling based on various combinations of MP2 and DLPNO-CCSD(T) as the high-tier methods and DFTB or PBE/pob-TZVP-rev2 as the medium-tier methods within the FrAMonC scheme. Performing the underlying repeated ab initio monomer calculations with atom-centered Gaussian orbitals is very cheap, and such MC sampling of the monomer energies costs only a fraction of the computational cost of FrAMonC sampling of the bulk. Therefore, the computational uncertainty is here governed by the number of averaged FrAMonC bulk configurations and no severe limitations arise from the monomer sampling.

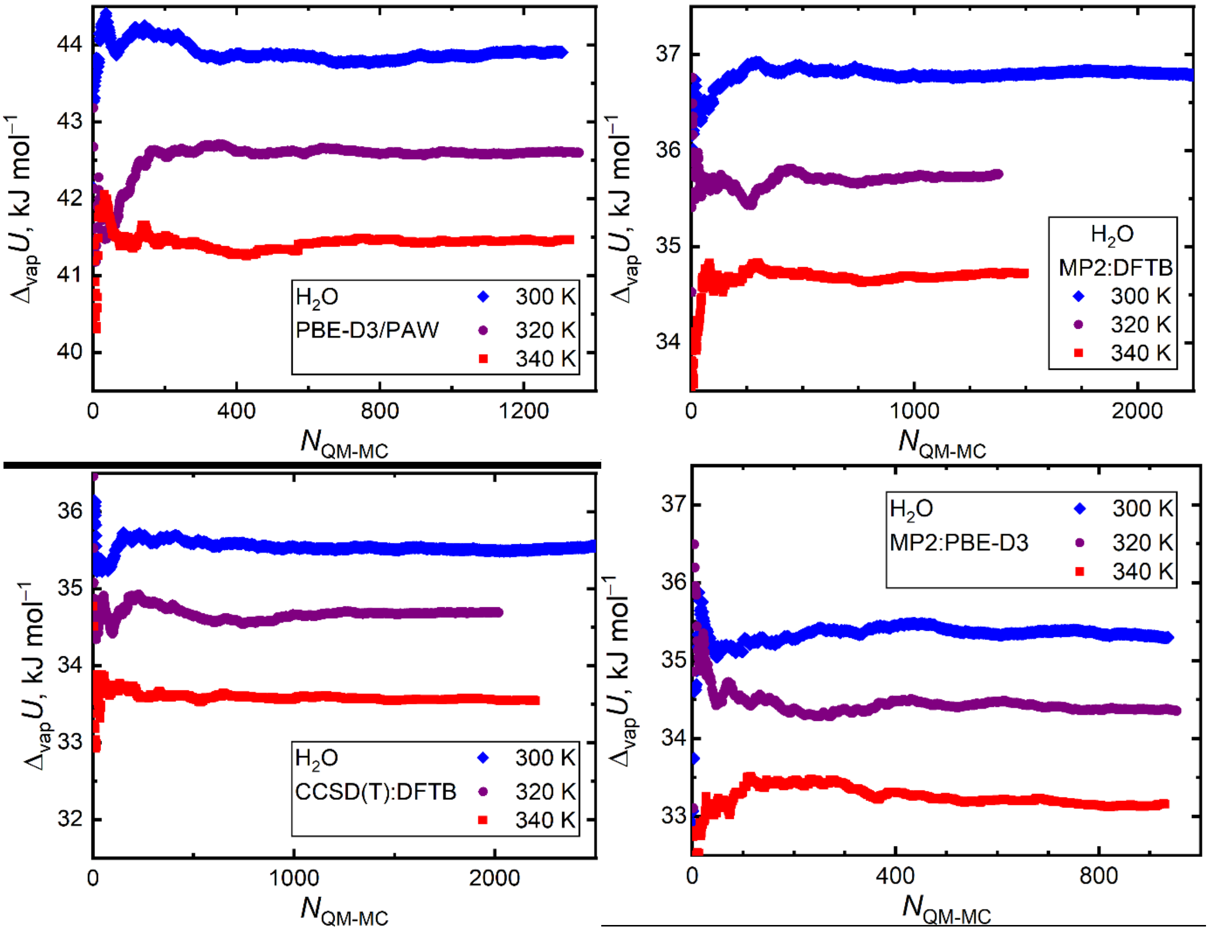

Figure S19. Running averages of vaporization energy of water as a function of the numbers of accepted configurations $N_{QM\text{-}MC}$ assembled during various QM MC simulations.

However, in the case of exploiting periodic DFT models, coupled with the PAW formalism, simulations of monomers in large void virtual cells becomes very costly both in terms of CPU time and memory.

Performing larger numbers of such repeated PBE-D3/PAW monomer calculations then results in a massive increase of the computational cost and such a computational workflow is clearly not efficient. Still, we performed for comparison such PBE-D3/PAW MC simulations of the monomers for benchmark purposes, somewhat limiting the lengths of those sampled monomer sequences, however to be able to accommodate the related computational demands. For this reason, there are less data points shown in Figure S19 for vaporization energy of water from PBE-D3/PAW calculations than above in Figure S11 for density of liquid water and similarly below for the remaining target materials.

Figure S20 and S21 illustrate the running average of vaporization energy for methanol and dimethyl ether, respectively. Assembling in general at least around 600 accepted configurations of both the liquid and vapor proved to be necessary in the FraMonC calculations to suppress the statistical sampling uncertainty in vaporization energy well below 0.5 kJ·mol^{-1}. Focusing on variation of the vaporization energy due to temperature, at least 400 such configurations of both phases was required to capture the correct hierarchy of the vaporization energy monotonously decreasing with temperature.

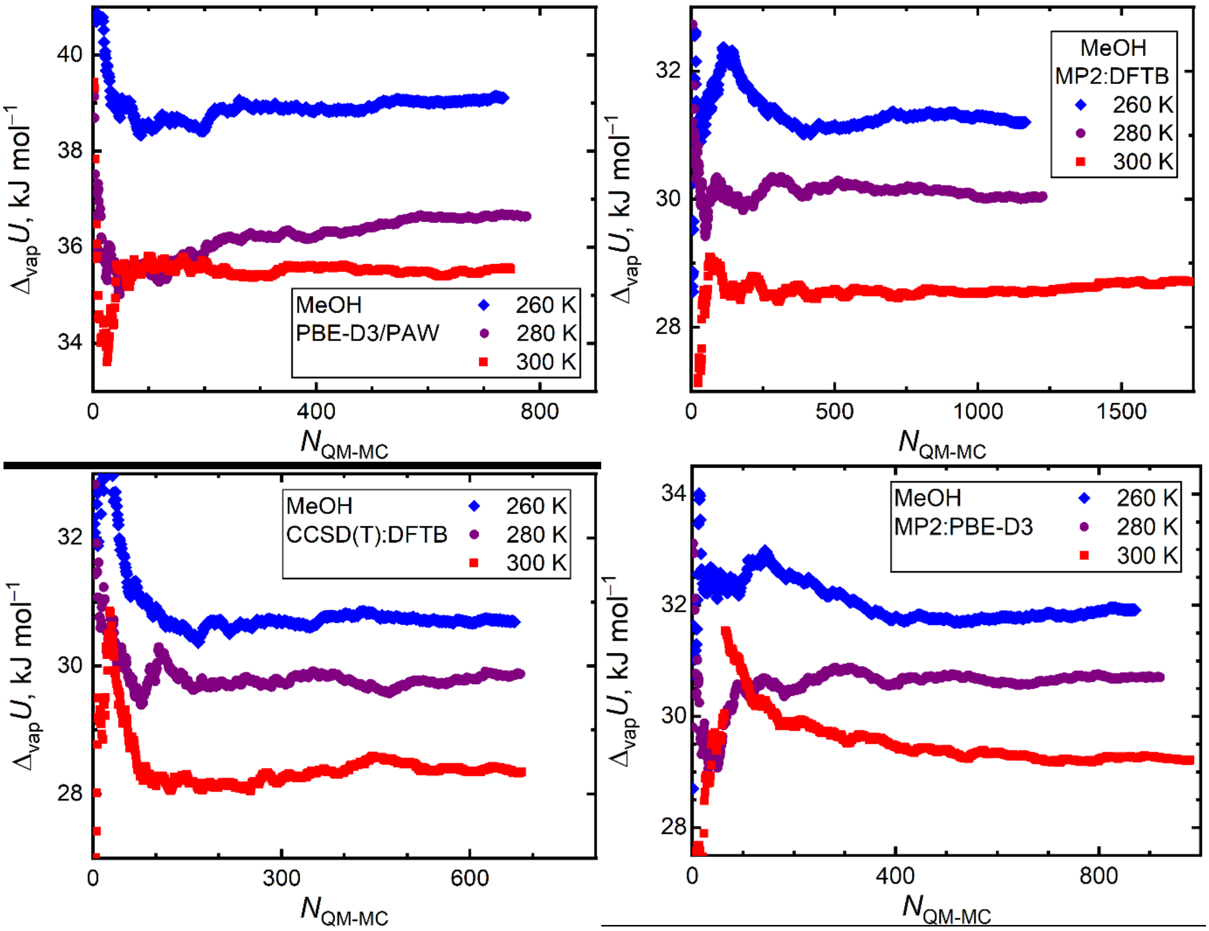

Figure S20. Running averages of vaporization energy of methanol as a function of the numbers of accepted configurations N_{QM-MC} assembled during various QM MC simulations.

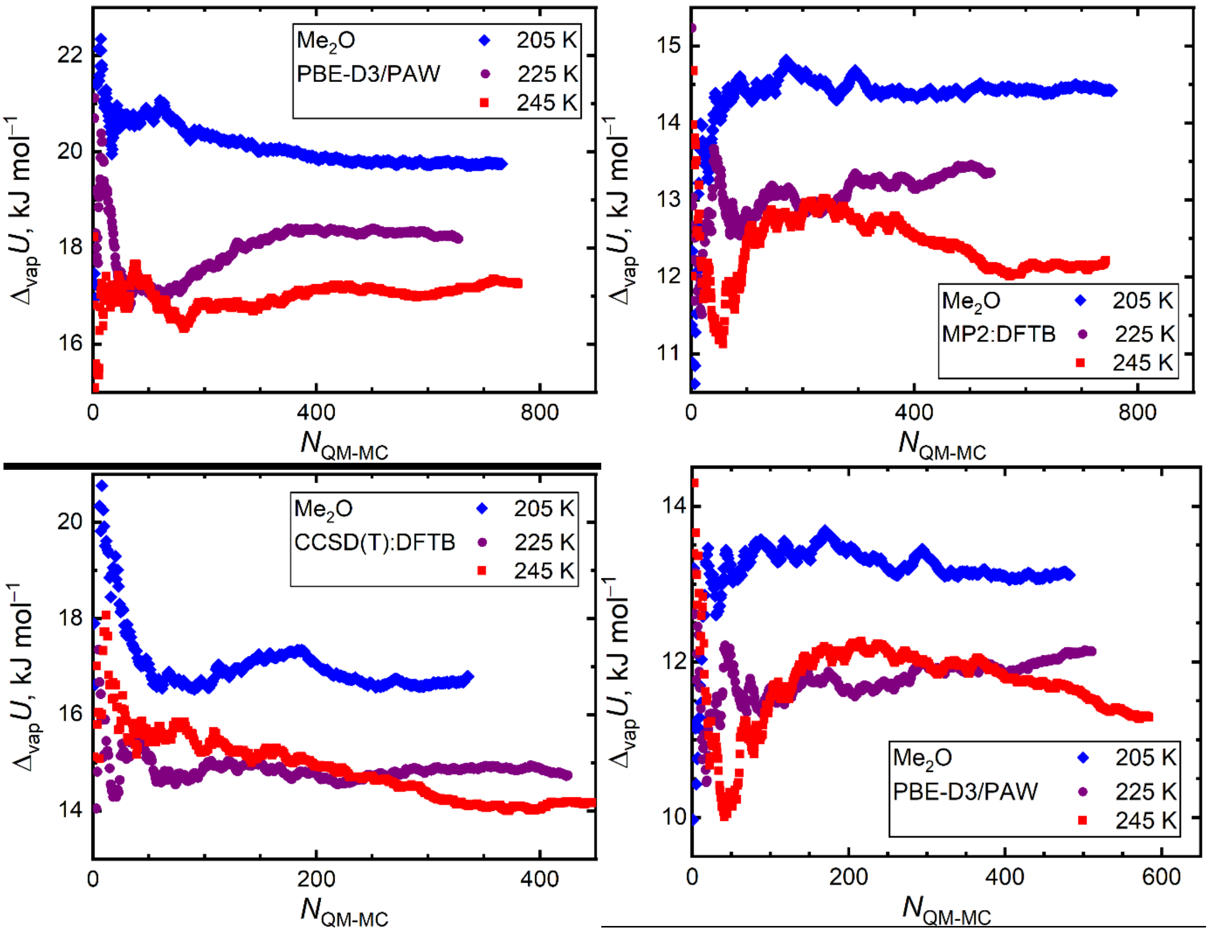

Figure S21. Running averages of vaporization energy of dimethyl ether as a function of the numbers of accepted configurations $N_{QM\text{-}MC}$ assembled during various QM MC simulations.

S4. Libraries of calculated molecular interactions

Apart from the macroscopic view on the ab initio vaporization energies, performing the FrAMonC simulations enables a detailed analysis of the individual pair interactions in the bulk that contribute to its cohesion the most. Focusing only on the first two solvation shells in the bulk liquid, Figure S22 depicts all pair interaction identified in the accepted FrAMonC configurations in liquid water at 300 K. Given the amorphous character of the bulk liquid, one can trace there a massive diversity of mutual orientations of proximate molecular pairs. As a consequence, the resulting data points of all pair interactions fill an area in the energy – distance coordinates the shape which is characteristic for each material. This area is delimited by virtual curves indicating from below and from above the most beneficial attractive and the most repulsive interactions that a molecular pair can form at a particular distance in its bulk liquid environment, respectively.

Provided that the FrAMonC simulations are run to assemble a sufficient number of accepted bulk configurations, the lower and rightmost regions of this energy-distance area are sampled completely. Density of the most repulsive recorded points in the topmost region then scales with Boltzmann probability of occurrence of such unfavorable arrangements of molecular pairs in the bulk structure. For water at 300 K, the most repulsive sampled pair interactions reached nearly 40 kJ·mol^{-1} at the MP2 level of theory, while the entire region of interactions above 15 kJ·mol^{-1} is sampled rather sparsely.

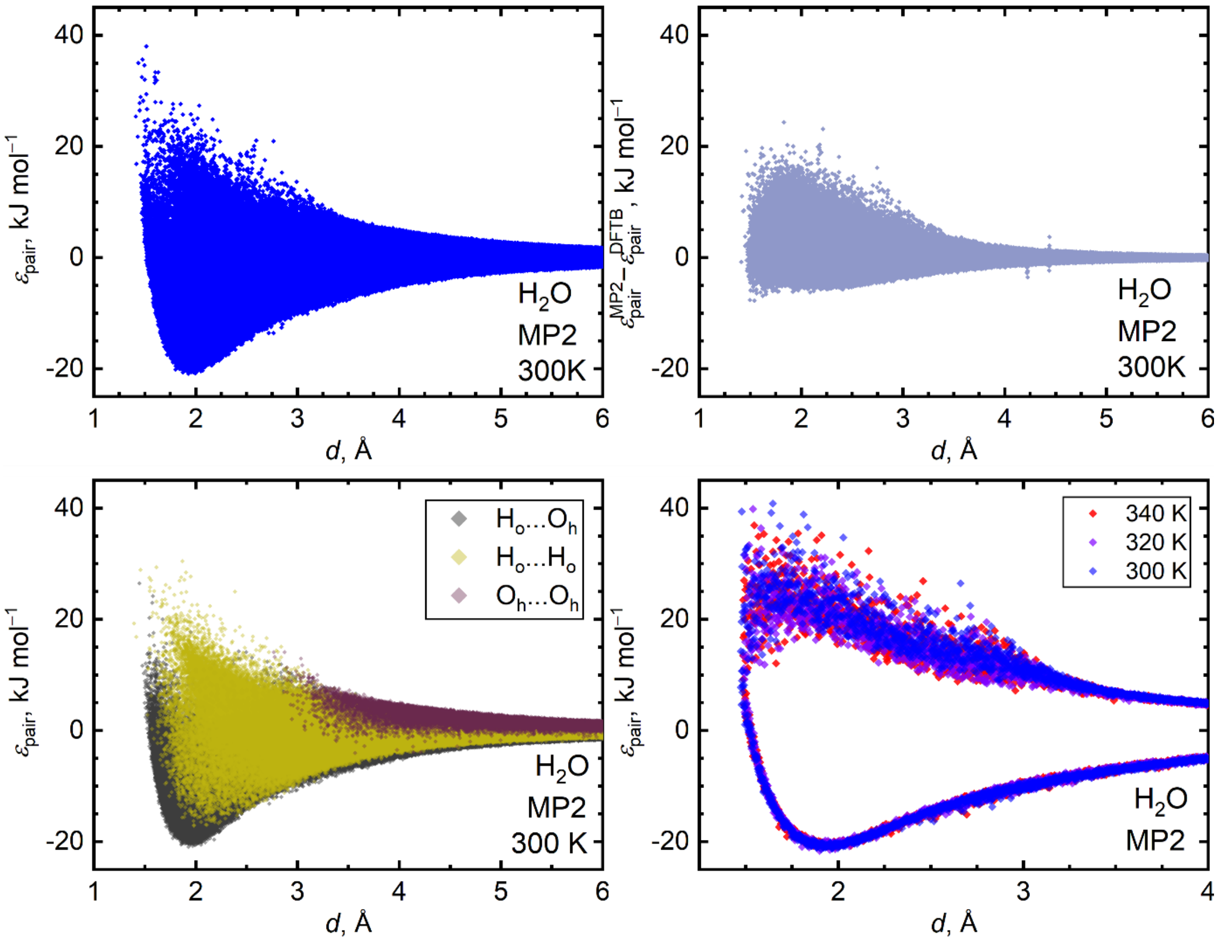

Figure S22. Pair interactions in liquid water: top left – pair interaction energies calculated at the RI-MP2-F12/aug-cc-pVDZ level of theory for dimer geometries extracted from FrAMonC accepted bulk liquid configurations; top right – difference between the same MP2 and DFTB pair interactions; bottom left – pair interaction energies classified according to the atoms forming the closest contact; bottom right – envelope plot of maximum and minimum pair interactions within 0.002 Å bins of the dimer separation. Libraries of pair interactions calculated in this work can be found in a public repository.[8]

Using a color-coding of individual dimer points with respect to the closest atom types that get into contact in each dimer in Figure S22b enables to compare the magnitudes of individual interaction types. As expected, the most attractive interactions of water dimers, reaching roughly −20 kJ·mol^{-1} in liquid water at 300 K according to the MP2 theory, are governed by hydrogen bonding. Its magnitude decays with the distance, rather quickly losing the hydrogen-bonded character and transforming to dispersion interactions. That observation can be supported by a very similar decay trend of interactions of two hydroxyl hydrogen atoms in bulk water beyond 2.5 Å. Observation of the closest recorded oxygen-oxygen contacts (molecular arrangement excluding the possibility of a hydrogen bond) in bulk water naturally reveals repulsive contacts governed by electrostatic interactions, with these contacts occurring predominantly beyond 3.0 Å. The closest repulsive contacts of two hydroxyl hydrogen are found to take place already at 1.7 Å. Importantly, even too close oxygen-hydrogen contacts do not necessarily result in a proper hydrogen bond as numerous such contacts already at 1.5 Å exhibit a strongly repulsive character, governed by exchange effects. Depicting only the most intense attractive and repulsive interactions recorded in particular distance intervals results in an envelope plot shown in Figure S22c. FrAMonC simulations also enable to quantify the difference between pair interactions computed at the high-tier level (here MP2) and a medium-tier level (here DFTB). Figure S22d illustrates that the differences in how both QM methods treat individual pair interactions are very large (being predominantly scattered from −7 to +20 kJ·mol^{-1}) when compared to the overall vaporization energy of

water. For the close interactions within roughly 3.0 Å, DFTB is predicted to overbind such dimers more frequently, while this distribution of ε^{MP2}–ε^{DFTB} is less biased at longer molecular separations.

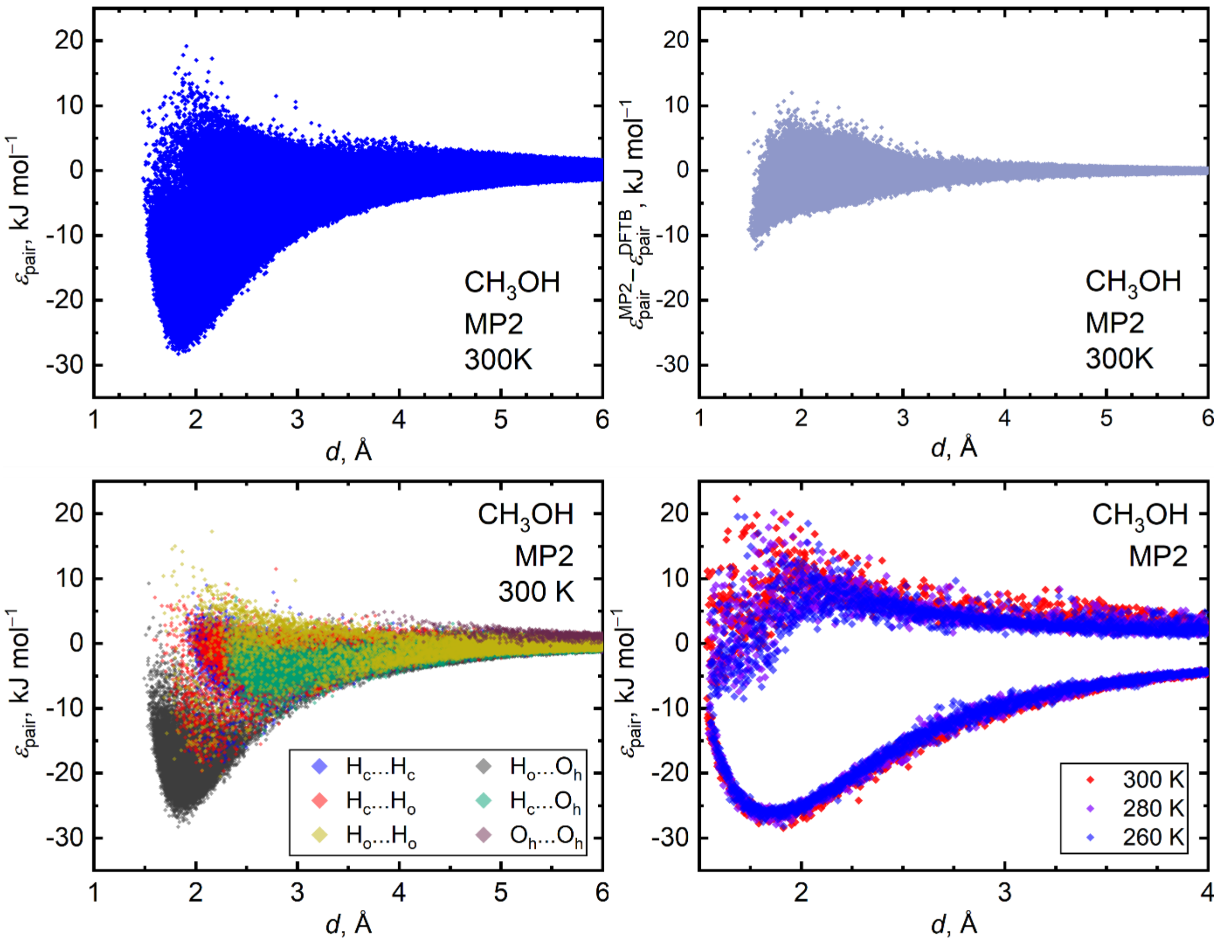

Figure S23. Pair interactions in liquid methanol: top left – pair interaction energies calculated at the RI-MP2-F12/aug-cc-pVDZ level of theory for dimer geometries extracted from FrAMonC accepted bulk liquid configurations; top right – difference between the same MP2 and DFTB pair interactions; bottom left – pair interaction energies classified according to the atoms forming the closest contact; bottom right – envelope plot of maximum and minimum pair interactions within 0.002 Å bins of the dimer separation. Libraries of pair interactions calculated in this work can be found in a public repository.[8]

An analogous ab initio analysis of pairwise interactions in bulk liquid methanol recorded in its FrAMonC simulations is presented in Figure S23. It reveals that the most attractive hydrogen bonds are somewhat stronger (reaching roughly −28 kJ·mol^{-1}) than those in water. On the other hand, significantly less repulsive molecular contacts were identified in liquid methanol, peaking at 20 kJ·mol^{-1}, but scarcely exceeding 10 kJ·mol^{-1}. Figure S23b reveals that methanol dimers with the closest hydrogen-hydrogen contacts can reach significantly more attractive character than those with the closest hydrogen-oxygen contacts. Repulsive oxygen-oxygen contacts are modeled to be appreciably less frequent at distances below 4.0 Å in liquid methanol, again differing from the situation in water. For liquid methanol, the envelop plot of pairwise interactions in Figure S23c shows that the sampling of the rare strongly repulsive dimers correlates with temperature. Occurrence of dimers located above 15 kJ·mol^{-1} is dominated by those extracted from methanol configurations at 300 K, representing the highest considered temperature in this case. Lowering the temperature leads to a drop of occurrence of such unfavorable repulsive dimers in the bulk liquid. Figure S23d illustrates that the differences in how both QM methods treat individual pair interactions reach up to ±10 kJ·mol^{-1}. For the closest rather repulsive interactions within 1.7 Å, DFTB seems to overstate their repulsion.

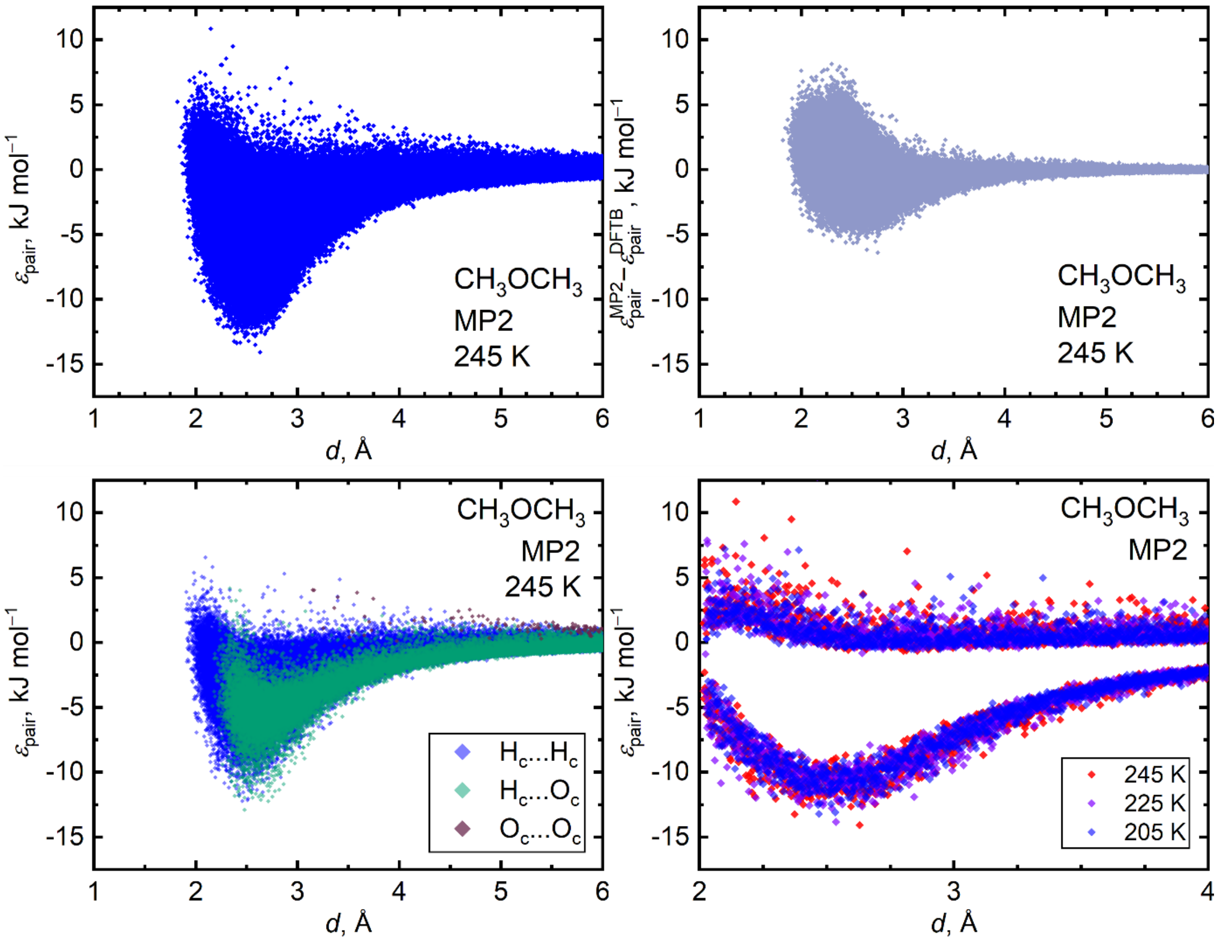

Figure S24. Pair interactions in liquid dimethyl ether: top left – pair interaction energies calculated at the RI-MP2-F12/aug-cc-pVDZ level of theory for dimer geometries extracted from FrAMonC accepted bulk liquid configurations; top right – difference between the same MP2 and DFTB pair interactions; bottom left – pair interaction energies classified according to the atoms forming the closest contact; bottom right – envelope plot of maximum and minimum pair interactions within 0.002 Å bins of the dimer separation. Libraries of pair interactions calculated in this work can be found in a public repository.[8]

For dimethyl ether, lacking any proper hydrogen bonds, the analysis of pairwise interactions, extracted from FrAMonC simulations of its bulk liquid results in significantly different plots in Figure S24. Recorded pairwise interactions in liquid dimethyl ether at 245 K range from −13 kJ·mol^{-1} (corresponding to both hydrogen-hydrogen and hydrogen-oxygen contacts) to +6 kJ·mol^{-1} (dominated by hydrogen-hydrogen contacts that occur also at closer separations). In general repulsive oxygen-oxygen contacts are very rare below 4.0 Å. The envelop plot in Figure S24c follows again the pattern with the most repulsive interactions above 5 kJ·mol^{-1} being recorded at the highest considered temperature, 245 K. Comparing the differences between DFTB and MP2 interaction energies, these values are somewhat lower than for the above discussed hydrogen-bonded materials, fitting within ±8 kJ·mol^{-1}.

Finally, a comparison of the envelop plots of pairwise interactions recorded in all three bulk liquids and evaluated using three QM levels of theory is given in Figure S25. This analysis shows that the most intense DFTB interaction energies of water tend to be shifted downwards, corresponding to stronger attraction (or weaker repulsion) when compared the ab initio results. The envelopes delimited in energy-distance coordinates by MP2 and DLPNO-CCSD(T) theories are very similar for bulk water then. On the other hand, Figure S25 also depicts that the envelops delimited the most intense interactions in bulk

methanol and dimethyl ether calculated by individual QM methods follow a systematic hierarchy with the envelop area enlarging in the row DFTB < DLPNO-CCSD(T) < MP2. That indicates that the MP2 level of theory tend to emphasize the most intense interactions manifested in bulk liquids, regardless of the sign of the interaction energy.

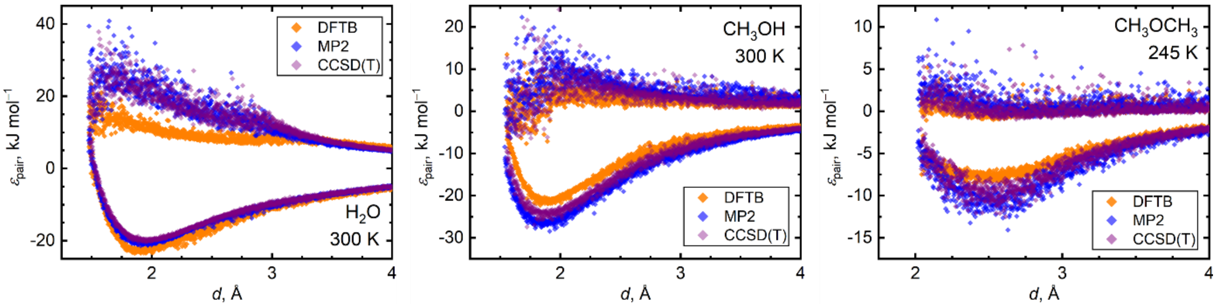

Figure S25. Envelope plots of pair interactions in liquid water, methanol and dimethyl ether at selected temperatures computed by various QM methods – maximum and minimum pair interactions within 0.002 Å bins of the dimer separation are shown.

Presented ab initio analyses of pairwise interactions extracted from the structure of bulk liquids is a valuable complement of the simulated radial distribution functions which do not include any explicit energetic information. Note that for water and methanol, the first peaks of the O…O $g(r)$ functions are located at closer distances than the oxygen-oxygen interactions start to appear in the plots of pairwise interactions in Figure S22 and S23. This is caused by the fact that the $g(r)$ functions also count hydrogen-bonded dimers where the oxygen atoms from both molecules are relatively close, but their distance naturally does not correspond the closest intermolecular contact which is given by the O…H distance. On the other hand for dimethyl ether, lacking such hydrogen bonding, position of the first peak of its O…O $g(r)$ function near 4.6 Å closely matches the distance where the closest oxygen-oxygen intermolecular contacts start to appear in Figure S24.

A numerical comparison of the most extreme recorded pairwise interactions (attractive, repulsive and closest) for all three bulk materials and three considered temperature for each is listed in Table S14. It confirms that molecules can instantaneously approach as close as roughly 1.48 Å, 1.55 Å, and 2.04 Å for water at 300 K, methanol at 300 K, and dimethyl ether at 245 K, respectively. Those very close contacts, however, do not persist for substantial time periods due to their overly repulsive character. Their relatively low concentration also does not completely disrupts the cohesion. At the same time, the exchange interactions posing steep potential walls prevent further approach of the molecules in the bulk, and thus effectively control the compressibility of a bulk material.

Table S14

Extreme molecular pair interactions extracted from FraMonC simulations of target liquids in terms of the minimum and maximum observed interaction energies (ε_{pair} , in kJ·mol^{-1}) and the closest atomic contact distance (d_{pair} , in Å)

Water						
Theory		MP2			CCSD(T)	
T	340 K	320 K	300 K	340 K	320 K	300 K
$\varepsilon_{pair}{}^{min}$	−20.991	−21.622	−21.391	−20.868	−20.573	−20.508
$d_{pair}{}^{min}$	1.486	1.490	1.474	1.470	1.478	1.478
$\varepsilon_{pair}{}^{max}$	36.951	39.896	40.852	37.306	38.778	37.855

			Methanol			
Theory		MP2			CCSD(T)	
T	300 K	280 K	260 K	300 K	280 K	260 K
$\varepsilon_{pair}{}^{min}$	−28.441	−27.902	−28.140	−27.399	−26.679	−26.059
$d_{pair}{}^{min}$	1.538	1.546	1.554	1.554	1.550	1.556
$\varepsilon_{pair}{}^{max}$	26.401	20.188	19.561	24.140	19.359	19.009
			Dimethyl ether			
Theory		MP2			CCSD(T)	
T	245 K	225 K	205 K	245 K	225 K	205 K
$\varepsilon_{pair}{}^{min}$	−14.056	−13.826	−13.211	−12.486	−12.123	−12.488
$d_{pair}{}^{min}$	2.016	2.022	2.028	2.038	2.052	2.058
$\varepsilon_{pair}{}^{max}$	10.865	12.758	7.228	7.833	7.774	6.906

Averaging the differences of all high-level, i. e. MP2 or DLPNO-CCSD(T), and medium-level, i. e. DFTB or PBE-D3, pair-interaction energies identified among all accepted FrAMonC liquid-phase configurations results in an overall correction to the cohesive (and also to vaporization) energy that the high-level method impart on the top of the medium-level method. Figure S26 to S28 depict results of this analysis of the scatter of all such instantaneous ab initio corrections along with their running average for liquid water, methanol, and dimethyl ether, respectively. Regardless of the studied system and a particular combination of the high- and medium-level methods, assembling over 300 accepted configurations suffices to converge this ab initio FrAMonC correction to within ±0.1 kJ mol^{-1} uncertainty with respect to the length of simulated FrAMonC sequences.

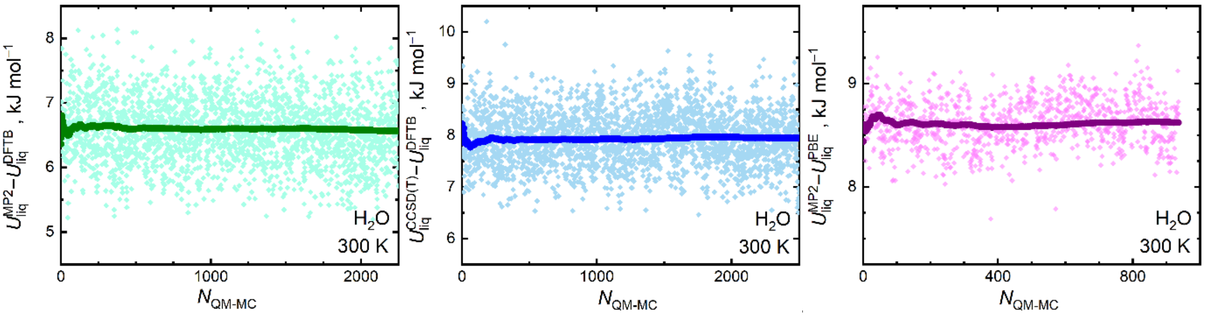

Figure S26. Distribution of instantaneous high-level (MP2 or DLPNO-CCSD(T)) corrections to the energy of liquid water at 300 K from the medium-level baseline (DFTB or PBE) and respective running average of such corrections shown as a function of the numbers of accepted configurations $N_{QM\text{-}MC}$ assembled during MP2:DFTB (left), DLPNO-CCSD(T):DFTB (center), and MP2:PBE (right) FraMonC simulations.

Comparing water with the remaining two target materials, these ab initio corrections to the liquid cohesion are repulsive, whereas they are attractive for methanol and dimethyl ether. Both MP2 and DLPNO-CCSD(T) corrections on the top of DFTB for water cohesion reach as much as 6.6 and 7.9 kJ mol^{-1}, respectively. Such corrections are appreciably smaller in magnitude for the latter materials, amounting to −3.0 and −1.7 kJ mol^{-1} for methanol, and ranging only to −0.3 and −0.7 kJ mol^{-1} for dimethyl ether. Interestingly, MP2 corrections to the cohesion on the top of PBE treatment exhibit an appreciably lower scatter of all materials than the MP2 corrections on the top of the DFTB cohesion.

That can be interpreted as if the PBE-D3(BJ) pair interaction energies were approaching the high-level MP2 results more closely than DFTB does. Notably, the average MP2 corrections on the top of PBE bulk liquid cohesion are significantly positive for all three materials, ranging from 3.8 kJ·mol^{-1} (dimethyl ether) to 8.7 kJ·mol^{-1} (water).

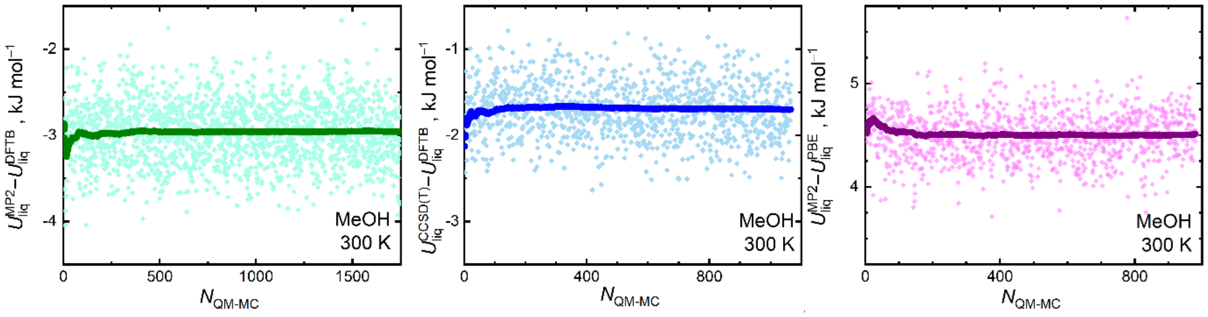

Figure S27. Distribution of instantaneous QM$_{high}$ corrections to the energy of liquid methanol at 300 K from the QM$_{med}$ baseline and respective running average of such corrections shown as a function of the numbers of accepted configurations $N_{QM\text{-}MC}$ assembled during MP2:DFTB (left), DLPNO-CCSD(T):DFTB (center), and DLPNO-CCSD(T):PBE (right) FraMonC simulations.

Interestingly, these presented ab initio corrections to the bulk liquid cohesion do not match the differences between vaporization enthalpies based purely on DFTB or PBE MC simulations and respective FrAMonC simulations exploiting DFTB or PBE as the medium-level method in the fragmentation scheme. Interpretation of this fact may be two-fold. First, the actual accepted configurations among individual FrAMonC simulations can somewhat differ, so that the molecular interactions can be calculated in both models for sufficiently distinct geometries of the closest molecular contacts. Secondly, there are also monomer corrections (due to distinct treatment of conformational energies in medium- and high-level QM models) included in the FrAMonC scheme.

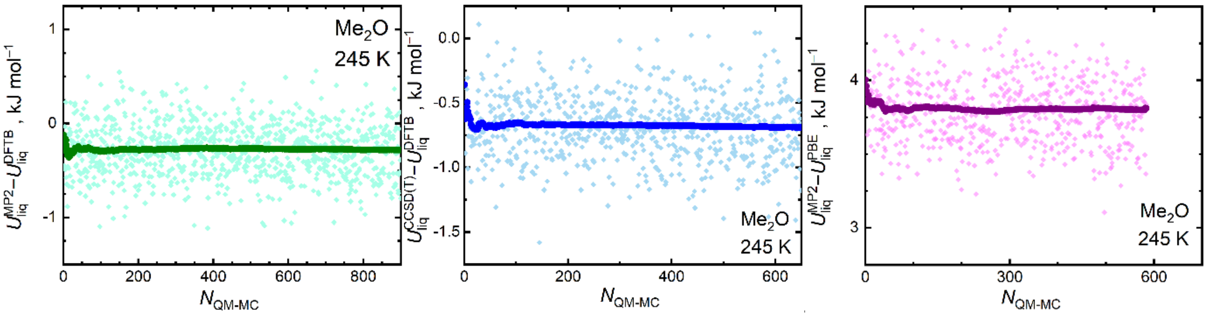

Figure S28. Distribution of instantaneous QM$_{high}$ corrections to the energy of liquid methanol at 245 K from the QM$_{med}$ baseline and respective running average of such corrections shown as a function of the numbers of accepted configurations $N_{QM\text{-}MC}$ assembled during MP2:DFTB (left), DLPNO-CCSD(T):DFTB (center), and DLPNO-CCSD(T):PBE (right) FraMonC simulations.

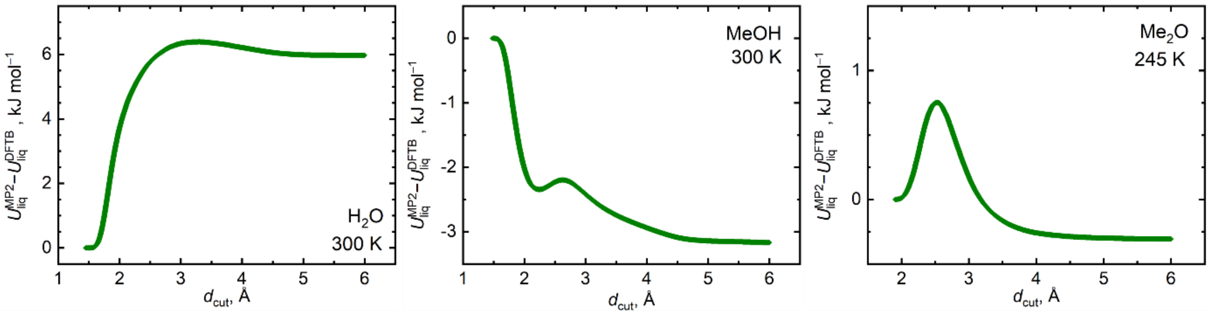

Figure S29. QM_{high} correction to the energy of the target bulk liquids at selected temperature as a function of the cut-off distance for explicit high-level QM treatment of proximate pair interactions within the bulk.

Figure S29. QMhigh correction to the energy of *the target bulk* liquids *at selected temperature as a function of the cut-off distance for explicit high-level QM treatment of proximate pair interactions within the bulk.*Figure S29 illustrates how the correction to the bulk-liquid energy (imposed in the FrAMonC scheme by correcting the pair interaction energies of proximate dimers from a medium-level method, here DFTB, to a high-level method, here MP2) varies with respect to the cut-off distance for the explicit high-level QM treatment of proximate pair interactions within the bulk material. It shows an important variation of this energy term throughout the first coordination shell at distances below 4 Å. There are only sub-kJ/mol variations of the energy term within the 4–6 Å interval, being in agreement with the integral data shown above in Table S12. Beyond this threshold, both medium-level and high-level QM methods indeed closely agree on the pair interaction energies, leaving only small potential for any substantial variation of the bulk liquid energy due to extending this cut-off distance.

Note that it has been shown in our earlier work that errors caused due to neglecting the long-range dispersion interactions (i. e. not using any medium-level method for the long-range effects) beyond 6 Å can lead to roughly 20% errors in the bulk energy of crystals of non-polar molecules.[29] However, in this case of the FrAMonC model where the long-range effects are captured at the medium-level QM theory, variation of the bulk energy is expected to be marginal, so that extending the cut-off for the explicit dimer treatment does not appear as the most suitable way how to improve the enthalpic accuracy. Instead, including also an explicit high-level QM treatment of proximate three-body interactions may be more appropriate for this purpose. Note that recent studies evaluating the impact of three-body interactions on the cohesion of molecular crystals indicate that high-level QM trimer treatment is necessary to approach the sub-chemical accuracy of bulk cohesion.[29, 31] Due to very high costs related to ab initio calculations of these three-body terms, we prefer not to perform those within the current study, be it a motivation for further development of the FrAMonC model.

S5. Details on computational costs

An extremely important aspect of any ab initio treatment of bulk liquids is the associated computational cost and the overall feasibility of such calculations. Table S15 lists the statistics about the computational performance and costs of the performed nested QM MC and FrAMonC simulations of the liquid phase. In general, the acceptance rate of the outer QM MC loop, being crucial to be maximized through the nesting scheme for an efficient workflow, is indeed maintained at very high values within an interval from 80% to 90%. In particular, such acceptance rates are reached also for the most costly and sophisticate FrAMonC scheme, justifying its computational efficiency. Interestingly, the lowest acceptance rates were reached for QM MC simulations relying on periodic PBE-D3/PAW theory. Also, the acceptance rate within the outer QM MC loop seems to be correlated with the molecular complexity, gradually decreasing from values for water (even exceeding 91% in some cases) to dimethyl ether (hardly exceeding 80%). Sampling of the conformational flexibility of molecules packed in bulk liquid and its interplay with relevant non-covalent interactions at the level of a classical force field inevitably deviates from the QM treatment. That leads to a somewhat looser similarity of energies of the bulk liquid sampled from both FF and QM potentials, propagating also to a slight decrease of the acceptance rates of the outer QM MC loops.

Table S15

Statistics about the computational performance of the outer loop (QM treatment) of performed MC simulations: generated number of QM-accepted configurations (N_{accept}), acceptance rate for the QM-assessed configurations (p_{accept}), real time (τ_{step} in seconds) required for one iteration of the outer QM loop (generation of the nested inner MC sequence and QM assessment of its final configuration), and number of CPU cores (n_{core} at AMD 7H12 2.6 GHz) used for the individual simulations.

						Water						
Theory	DFTB3-D4			PBE-D3			MP2-F12 [a]			DLPNO-CCSD(T) [a]		
T, K	300	320	340	300	320	340	300	320	340	300	320	340
N_{accept}	4219	7055	7134	1912	2605	2593	1963	1309	1211	2735	2017	2201
p_{accept}	0.913	0.915	0.914	0.790	0.826	0.821	0.912	0.902	0.770	0.907	0.852	0.902
τ_{step}	56	45	44	307	293	293	641	713	713	2220	2176	2112
n_{core}	16	16	16	64	64	64	128	128	128	128	128	128
						Methanol						
Theory	DFTB3-D4			PBE-D3			MP2-F12 [a]			DLPNO-CCSD(T) [a]		
T, K	260	280	300	260	280	300	260	280	300	260	280	300
N_{accept}	7058	6986	4361	2268	1841	2978	1164	1225	1768	1179	1263	811
p_{accept}	0.872	0.874	0.880	0.779	0.801	0.814	0.826	0.851	0.849	0.782	0.824	0.767
τ_{step}	43	43	52	311	330	333	734	719	663	5082	5001	4835
n_{core}	16	16	16	64	64	64	128	128	128	128	128	128
						Dimethyl ether						
Theory	DFTB3-D4			PBE-D3			MP2-F12 [a]			DLPNO-CCSD(T) [a]		
T, K	205	225	245	205	225	245	205	225	245	205	225	245
N_{accept}	6182	5653	3780	1330	1411	1445	662	691	743	480	499	604
p_{accept}	0.838	0.825	0.831	0.652	0.692	0.714	0.778	0.788	0.819	0.707	0.803	0.744
τ_{step}	47	50	57	438	458	514	1819	1766	1706	11073	11113	11782
n_{core}	16	16	16	64	64	64	128	128	128	128	128	128

[a] All FrAMonC simulations used a 4 Å cut-off distance for explicit ab initio high-level treatment and DFTB as the medium-level method for proximate pair interactions in the structure of bulk liquid.

Table S16
Statistics about the computational performance of the outer loop (QM treatment) of performed MC simulations: generated number of QM-accepted configurations (N_{accept}), acceptance rate for the QM-assessed configurations (p_{accept}), real time (τ_{step} in seconds) required for one iteration of the outer QM loop (generation of the nested inner MC sequence and QM assessment of its final configuration), and number of CPU cores (n_{core} at AMD 7H12 2.6 GHz) used for the individual simulations.

Method	MP2-F12:PBE-D3(BJ)								
Material	Water			Methanol			Dimethyl ether		
T, K	300	320	340	260	280	300	205	225	245
N_{accept}	936	953	930	979	920	871	482	511	584
p_{accept}	0.860	0.849	0.867	0.817	0.806	0.800	0.744	0.733	0.777
τ_{step}	4233	4116	3990	4224	4048	3868	6864	6444	5984
n_{core}	128	128	128	128	128	128	128	128	128

[a] All FrAMonC simulations used a 4 Å cut-off distance for explicit ab initio high-level treatment and PBE-D3(BJ) as the medium-level method for proximate pair interactions in the structure of bulk liquid.

Table S16 lists computational statistics of FrAMonC simulations relying on the MP2-F12:PBE-D3(BJ) composite approach. Comparing these values with those for MP2-F12:DFTB3-D4 in Table S15, it can be seen that the acceptance rates of the QM assessment of configurations within the FrAMonC scheme are somewhat lower when PBE-D3(BJ) is used as the medium-level theory therein. That is consistent with the fact that QM MC relying on the periodic PBE-D3(BJ)/PAW theory itself yielded the lowest acceptance rates.

Commenting on the particular consumed processor wall times, DFTB MC enabled us to generate 1.0-1.4 considered liquid-phase configurations per second to be treated within the outer QM MC loop when run on 16 CPU cores. QM MC relying on periodic PBE-D3/PAW was observed to be more expensive by a factor of 30-40 than DFTB MC when the higher number of CPU cores was considered as well. Adopting the MP2:DFTB scheme within FrAMonC led then to an additional four- to eight-fold costs increase over PBE-D3/PAW MC. Substituting of MP2 with DLPNO-CCSD(T) led then to additional costs increase by a factor of 3 for water and roughly 6 to 7 for both methanol and dimethyl ether. Such timings correspond to generating 100 accepted DLPNO-CCSD(T):DFTB FrAMonC configurations of liquid water on 128 CPU cores within about 60 hours, further prolonging to 140 and 315 hours for liquid methanol and dimethyl ether, respectively.

Furthermore, computational cost required to generate and perform a QM assessment of a single configuration using the MP2-F12:PBE-D3(BJ) FrAMonC scheme is roughly doubled when compared to the cost of DLPNO-CCSD(T):DFTB3-D4 scheme for water, representing the smallest considered molecule for which the monomer and dimer calculations at the DLPNO-CCSD(T) level of theory are still relatively cheap and the computational cost is largely affected by performing the periodic PBE-D3(BJ)/GTO calculations. Significant costs of using the PBE-D3(BJ)/GTO as the medium-level method can be also documented by the five- to six-fold cost increase per QM configuration assessment when compared to the FrAMonC simulations relying on the MP2-F12:DFTB3-D4 composite setup. On the other hand, for the remaining target molecules being larger than water, the associated costs of DLPNO-

CCSD(T) treatment of monomers and dimers already exceeds that of the PBE-D3(BJ)/GTO treatment within the medium-level regime, as listed in Table S15 and Table S16.

Table S17

Statistics about the computational performance of the outer loop (QM treatment) of performed FrAMonC simulations: generated number of QM-accepted configurations (N_{accept}), acceptance rate for the QM-assessed configurations (p_{accept}), real time (τ_{step} in seconds) required for one iteration of the outer QM loop (generation of the nested inner MC sequence and QM assessment of its final configuration), and average number of unique pair interactions found in the structure of bulk liquid within the specified cut-off distance (d_{cut}) in Å. All simulations were run on 128 AMD 7H12 2.6 GHz CPU cores.

			Water at 300 K			
Theory	MP2-F12				DLPNO-CCSD(T)	
d_{cut}, Å	3	4	5	6	3	4
N_{accept}	2703	1963	2171	1827	1239	2735
p_{accept}	0.916	0.912	0.906	0.904	0.912	0.907
τ_{step}	351	641	1184	1870	1141	2220
N_{pairs}	338	713	1382	2197	338	713
			Methanol at 300 K			
Theory	MP2-F12				DLPNO-CCSD(T)	
d_{cut}, Å	3	4	5	6	3	4
N_{accept}	2396	1768	1762	1228	827	811
p_{accept}	0.845	0.849	0.820	0.838	0.852	0.767
τ_{step}	431	663	963	1408	3173	4835
N_{pairs}	170	302	457	719	170	301
			Dimethyl ether at 245 K			
Theory	MP2-F12				DLPNO-CCSD(T)	
d_{cut}, Å	3	4	5	6	3	4
N_{accept}	1073	743	562	407	290	604
p_{accept}	0.800	0.819	0.801	0.816	0.799	0.744
τ_{step}	1156	1706	2201	3088	8362	11782
N_{pairs}	150	239	321	478	150	232

Computational performance of the FrAMonC simulations naturally depends on a particular setup of the fragmentation protocol, leading to identification and explicit QM treatment of relevant monomer and dimer contributions to the bulk cohesion. Table S17 summarizes variations of the FrAMonC computational costs with respect of the cut-off distance for the dimers to be treated with the high-level QM method. For water, increasing this cut-off distance by every 1 Å results in a 55%-85% increase of the computational costs as many more individual interactions need to be computed explicitly, whereas these values range within 44%-55% for methanol, and 30%-50% for dimethyl ether. Imposing the cut-off for explicit dimer treatment at the 4 Å distance maintains the computational complexity within reasonable range to make the FrAMonC simulations viable.

References to the SI

(1) Shah, J. K.; Marin-Rimoldi, E.; Mullen, R. G.; Keene, B. P.; Khan, S.; Paluch, A. S.; Rai, N.; Romanielo, L. L.; Rosch, T. W.; Yoo, B.; et al. Cassandra: An open source Monte Carlo package for molecular simulation. *Journal of Computational Chemistry* **2017**, *38* (19), 1727–1739. DOI: https://doi.org/10.1002/jcc.24807 (acccessed 2024/10/23).
(2) Jorgensen, W. L.; Chandrasekhar, J.; Madura, J. D.; Impey, R. W.; Klein, M. L. Comparison of simple potential functions for simulating liquid water. *The Journal of Chemical Physics* **1983**, *79* (2), 926–935. DOI: 10.1063/1.445869 (acccessed 10/23/2024).
(3) Jorgensen, W. L.; Maxwell, D. S.; Tirado-Rives, J. Development and Testing of the OPLS All-Atom Force Field on Conformational Energetics and Properties of Organic Liquids. *Journal of the American Chemical Society* **1996**, *118* (45), 11225–11236. DOI: 10.1021/ja9621760.
(4) Shah, J. K.; Maginn, E. J. A general and efficient Monte Carlo method for sampling intramolecular degrees of freedom of branched and cyclic molecules. *The Journal of Chemical Physics* **2011**, *135* (13). DOI: 10.1063/1.3644939 (acccessed 10/23/2024).
(5) Metropolis, N.; Rosenbluth, A. W.; Rosenbluth, M. N.; Teller, A. H.; Teller, E. Equation of State Calculations by Fast Computing Machines. *The Journal of Chemical Physics* **1953**, *21* (6), 1087–1092. DOI: 10.1063/1.1699114 (acccessed 3/18/2025).
(6) Iftimie, R.; Salahub, D.; Wei, D.; Schofield, J. Using a classical potential as an efficient importance function for sampling from an ab initio potential. *The Journal of Chemical Physics* **2000**, *113* (12), 4852–4862. DOI: 10.1063/1.1289534 (acccessed 7/3/2025).
(7) Leiding, J.; Coe, J. D. An efficient approach to ab initio Monte Carlo simulation. *The Journal of Chemical Physics* **2014**, *140* (3). DOI: 10.1063/1.4855755 (acccessed 10/22/2024).
(8) Červinka, C. *FrAMonC github repository*. 2025. https://github.com/CervinkaGroup/FrAMonC (accessed.
(9) Gaus, M.; Cui, Q.; Elstner, M. DFTB3: Extension of the Self-Consistent-Charge Density-Functional Tight-Binding Method (SCC-DFTB). *Journal of Chemical Theory and Computation* **2011**, *7* (4), 931–948. DOI: 10.1021/ct100684s.
(10) Gaus, M.; Goez, A.; Elstner, M. Parametrization and Benchmark of DFTB3 for Organic Molecules. *Journal of Chemical Theory and Computation* **2013**, *9* (1), 338–354. DOI: 10.1021/ct300849w.
(11) Kubillus, M.; Kubař, T.; Gaus, M.; Řezáč, J.; Elstner, M. Parameterization of the DFTB3 Method for Br, Ca, Cl, F, I, K, and Na in Organic and Biological Systems. *Journal of Chemical Theory and Computation* **2015**, *11* (1), 332–342. DOI: 10.1021/ct5009137.
(12) Caldeweyher, E.; Ehlert, S.; Hansen, A.; Neugebauer, H.; Spicher, S.; Bannwarth, C.; Grimme, S. A generally applicable atomic-charge dependent London dispersion correction. *The Journal of Chemical Physics* **2019**, *150* (15). DOI: 10.1063/1.5090222 (acccessed 10/23/2024).
(13) Hourahine, B.; Aradi, B.; Blum, V.; Bonafé, F.; Buccheri, A.; Camacho, C.; Cevallos, C.; Deshaye, M. Y.; Dumitrică, T.; Dominguez, A.; et al. DFTB+, a software package for efficient approximate density functional theory based atomistic simulations. *The Journal of Chemical Physics* **2020**, *152* (12). DOI: 10.1063/1.5143190 (acccessed 11/24/2023).
(14) Ludík, J.; Kostková, V.; Kocian, Š.; Touš, P.; Štejfa, V.; Červinka, C. First-Principles Models of Polymorphism of Pharmaceuticals: Maximizing the Accuracy-to-Cost Ratio. *Journal of Chemical Theory and Computation* **2024**, *20* (7), 2858–2870. DOI: 10.1021/acs.jctc.4c00099.
(15) Perdew, J. P.; Burke, K.; Ernzerhof, M. Generalized Gradient Approximation Made Simple. *Physical Review Letters* **1996**, *77* (18), 3865–3868. DOI: 10.1103/PhysRevLett.77.3865.
(16) Grimme, S.; Antony, J.; Ehrlich, S.; Krieg, H. A consistent and accurate ab initio parametrization of density functional dispersion correction (DFT-D) for the 94 elements H-Pu. *The Journal of Chemical Physics* **2010**, *132* (15). DOI: 10.1063/1.3382344 (acccessed 10/23/2024). Grimme, S.; Ehrlich, S.; Goerigk, L. Effect of the damping function in dispersion corrected density functional theory. *Journal of Computational Chemistry* **2011**, *32* (7), 1456–1465. DOI: https://doi.org/10.1002/jcc.21759 (acccessed 2024/10/23).
(17) Kresse, G.; Furthmüller, J. Efficient iterative schemes for ab initio total-energy calculations using a plane-wave basis set. *Physical Review B* **1996**, *54* (16), 11169–11186. DOI: 10.1103/PhysRevB.54.11169. Kresse, G.; Furthmüller, J. Efficiency of ab-initio total energy

calculations for metals and semiconductors using a plane-wave basis set. *Computational Materials Science* **1996**, *6* (1), 15–50. DOI: https://doi.org/10.1016/0927-0256(96)00008-0.
(18) Blöchl, P. E. Projector augmented-wave method. *Physical Review B* **1994**, *50* (24), 17953–17979. DOI: 10.1103/PhysRevB.50.17953.
(19) Kresse, G.; Joubert, D. From ultrasoft pseudopotentials to the projector augmented-wave method. *Phys. Rev. B* **1999**, *59* (3), 1758–1775. DOI: 10.1103/PhysRevB.59.1758.
(20) Červinka, C.; Fulem, M.; Stoffel, R. P.; Dronskowski, R. Thermodynamic properties of molecular crystals calculated within the quasi-harmonic approximation. *J. Phys. Chem. A* **2016**, *120*, 2022–2034.
(21) Červinka, C.; Klajmon, M.; Štejfa, V. Cohesive Properties of Ionic Liquids Calculated from First Principles. *Journal of Chemical Theory and Computation* **2019**, *15* (10), 5563–5578. DOI: 10.1021/acs.jctc.9b00625. Červinka, C.; Štejfa, V. Sublimation Properties of alpha,omega-Diamines Revisited from First-Principles Calculations. *Chemphyschem* **2020**, *21* (11), 1184–1194. DOI: 10.1002/cphc.202000108.
(22) Touš, P.; Červinka, C. Dynamic Disorder, Strain, and Sublimation of Crystalline Caged Hydrocarbons from First Principles. *Crystal Growth & Design* **2023**, *23* (6), 4082–4097. DOI: 10.1021/acs.cgd.2c01496.
(23) Červinka, C.; Fulem, M. Cohesive properties of the crystalline phases of twenty proteinogenic α-aminoacids from first-principles calculations. *Physical Chemistry Chemical Physics* **2019**, *21* (34), 18501–18515, 10.1039/C9CP03102B. DOI: 10.1039/C9CP03102B.
(24) Weatherby, J. A.; Rumson, A. F.; Price, A. J. A.; de la Roza, A. O.; Johnson, E. R. A density-functional benchmark of vibrational free-energy corrections for molecular crystal polymorphism. *Journal of Chemical Physics* **2022**, *156* (11). DOI: 10.1063/5.0083082.
(25) Červinka, C. Tuning the quasi-harmonic treatment of crystalline ionic liquids within the density functional theory. *Journal of Computational Chemistry* **2022**, *43* (7), 448–456, https://doi.org/10.1002/jcc.26804. DOI: https://doi.org/10.1002/jcc.26804 (acccessed 2022/09/20). Dolgonos, G. A.; Hoja, J.; Boese, A. D. Revised values for the X23 benchmark set of molecular crystals. *Physical Chemistry Chemical Physics* **2019**, *21* (44), 24333–24344, 10.1039/C9CP04488D. DOI: 10.1039/C9CP04488D. Moellmann, J.; Grimme, S. DFT-D3 Study of Some Molecular Crystals. *J. Phys. Chem. C* **2014**, *118* (14), 7615–7621. DOI: 10.1021/jp501237c. Reilly, A. M.; Tkatchenko, A. Understanding the Role of Vibrations, Exact Exchange, and Many-body van der Waals Interactions in the Cohesive Properties of Molecular Crystals. *J. Chem. Phys.* **2013**, *139* (2), 024705. DOI: 10.1063/1.4812819.
(26) Herbert, J. M. Fantasy versus reality in fragment-based quantum chemistry. *The Journal of Chemical Physics* **2019**, *151* (17). DOI: 10.1063/1.5126216 (acccessed 3/18/2025).
(27) Beran, G. J. O. Modeling Polymorphic Molecular Crystals with Electronic Structure Theory. *Chemical Reviews* **2016**, *116* (9), 5567–5613. DOI: 10.1021/acs.chemrev.5b00648.
(28) Červinka, C.; Beran, G. J. O. Ab initio prediction of the polymorph phase diagram for crystalline methanol. *Chemical Science* **2018**, *9* (20), 4622–4629, 10.1039/C8SC01237G. DOI: 10.1039/C8SC01237G.
(29) Červinka, C.; Fulem, M.; Růžička, K. CCSD(T)/CBS fragment-based calculations of lattice energy of molecular crystals. *The Journal of Chemical Physics* **2016**, *144* (6). DOI: 10.1063/1.4941055 (acccessed 3/11/2025).
(30) Yang, J.; Hu, W.; Usvyat, D.; Matthews, D.; Schütz, M.; Chan, G. K.-L. Ab initio determination of the crystalline benzene lattice energy to sub-kilojoule/mole accuracy. *Science* **2014**, *345* (6197), 640–643. DOI: doi:10.1126/science.1254419.
(31) Hoja, J.; List, A.; Boese, A. D. Multimer Embedding Approach for Molecular Crystals up to Harmonic Vibrational Properties. *Journal of Chemical Theory and Computation* **2024**, *20* (1), 357–367. DOI: 10.1021/acs.jctc.3c01082.
(32) Heit, Y. N.; Nanda, K. D.; Beran, G. J. O. Predicting Finite-Temperature Properties of Crystalline Carbon Dioxide from First Principles with Quantitative Accuracy. *Chem. Sci.* **2016**, *7* (1), 246–255. DOI: 10.1039/c5sc03014e.
(33) Pokorný, V.; Touš, P.; Štejfa, V.; Růžička, K.; Rohlíček, J.; Czernek, J.; Brus, J.; Červinka, C. Anisotropy, segmental dynamics and polymorphism of crystalline biogenic carboxylic acids. *Physical Chemistry Chemical Physics* **2022**, *24* (42), 25904–25917, 10.1039/D2CP03698C. DOI: 10.1039/D2CP03698C.

(34) Maschio, L.; Usvyat, D.; Schütz, M.; Civalleri, B. Periodic local Møller–Plesset second order perturbation theory method applied to molecular crystals: Study of solid NH3 and CO2 using extended basis sets. *The Journal of Chemical Physics* **2010**, *132* (13). DOI: 10.1063/1.3372800 (acccessed 3/18/2025).
(35) McKinley, Jessica L.; Beran, G. J. O. Identifying pragmatic quasi-harmonic electronic structure approaches for modeling molecular crystal thermal expansion. *Faraday Discussions* **2018**, *211* (0), 181–207, 10.1039/C8FD00048D. DOI: 10.1039/C8FD00048D. Hirata, S.; Gilliard, K.; He, X.; Li, J.; Sode, O. Ab Initio Molecular Crystal Structures, Spectra, and Phase Diagrams. *Acc. Chem. Res.* **2014**, *47* (9), 2721–2730. DOI: 10.1021/ar500041m. Bygrave, P. J.; Allan, N. L.; Manby, F. R. The embedded many-body expansion for energetics of molecular crystals. *The Journal of Chemical Physics* **2012**, *137* (16). DOI: 10.1063/1.4759079 (acccessed 3/18/2025). Ringer, A. L.; Sherrill, C. D. First Principles Computation of Lattice Energies of Organic Solids: The Benzene Crystal. *Chemistry – A European Journal* **2008**, *14* (8), 2542–2547. DOI: https://doi.org/10.1002/chem.200701622. Schweizer, W. B.; Dunitz, J. D. Quantum Mechanical Calculations for Benzene Dimer Energies: Present Problems and Future Challenges. *Journal of Chemical Theory and Computation* **2006**, *2* (2), 288–291. DOI: 10.1021/ct0502357.
(36) Heit, Y.; Beran, G. J. O. Exploiting space-group symmetry in fragment-based molecular crystal calculations. *Journal of Computational Chemistry* **2014**, *35* (30), 2205–2214. DOI: https://doi.org/10.1002/jcc.23737.
(37) Bore, S. L.; Paesani, F. Realistic phase diagram of water from "first principles" data-driven quantum simulations. *Nature Communications* **2023**, *14* (1), 3349. DOI: 10.1038/s41467-023-38855-1. Gupta, S.; Bull-Vulpe, E. F.; Agnew, H.; Iyer, S.; Zhu, X.; Zhou, R.; Knight, C.; Paesani, F. MBX V1.2: Accelerating Data-Driven Many-Body Molecular Dynamics Simulations. *Journal of Chemical Theory and Computation* **2025**, *21* (4), 1838–1849. DOI: 10.1021/acs.jctc.4c01333. Muniz, M. C.; Gartner, T. E., III; Riera, M.; Knight, C.; Yue, S.; Paesani, F.; Panagiotopoulos, A. Z. Vapor–liquid equilibrium of water with the MB-pol many-body potential. *The Journal of Chemical Physics* **2021**, *154* (21). DOI: 10.1063/5.0050068 (acccessed 6/28/2025).
(38) Kříž, K.; Řezáč, J. Benchmarking of Semiempirical Quantum-Mechanical Methods on Systems Relevant to Computer-Aided Drug Design. *Journal of Chemical Information and Modeling* **2020**, *60* (3), 1453–1460. DOI: 10.1021/acs.jcim.9b01171. Řezáč, J. Non-Covalent Interactions Atlas Benchmark Data Sets: Hydrogen Bonding. *Journal of Chemical Theory and Computation* **2020**, *16* (4), 2355–2368. DOI: 10.1021/acs.jctc.9b01265.
(39) Vilela Oliveira, D.; Laun, J.; Peintinger, M. F.; Bredow, T. BSSE-correction scheme for consistent gaussian basis sets of double- and triple-zeta valence with polarization quality for solid-state calculations. *Journal of Computational Chemistry* **2019**, *40* (27), 2364–2376. DOI: https://doi.org/10.1002/jcc.26013.
(40) Werner, H.-J.; Adler, T. B.; Manby, F. R. General orbital invariant MP2-F12 theory. *The Journal of Chemical Physics* **2007**, *126* (16). DOI: 10.1063/1.2712434 (acccessed 3/18/2025).
(41) Liakos, D. G.; Róbert, I.; F., V. E.; and Neese, F. What is the most efficient way to reach the canonical MP2 basis set limit? *Molecular Physics* **2013**, *111* (16-17), 2653–2662. DOI: 10.1080/00268976.2013.824624.
(42) Kossmann, S.; Neese, F. Comparison of two efficient approximate Hartee–Fock approaches. *Chemical Physics Letters* **2009**, *481* (4), 240–243. DOI: https://doi.org/10.1016/j.cplett.2009.09.073.
(43) Weigend, F.; Köhn, A.; Hättig, C. Efficient use of the correlation consistent basis sets in resolution of the identity MP2 calculations. *The Journal of Chemical Physics* **2002**, *116* (8), 3175–3183. DOI: 10.1063/1.1445115 (acccessed 3/18/2025).
(44) Peterson, K. A.; Adler, T. B.; Werner, H.-J. Systematically convergent basis sets for explicitly correlated wavefunctions: The atoms H, He, B–Ne, and Al–Ar. *The Journal of Chemical Physics* **2008**, *128* (8). DOI: 10.1063/1.2831537 (acccessed 3/18/2025).
(45) Řezáč, J.; Hobza, P. Describing Noncovalent Interactions beyond the Common Approximations: How Accurate Is the "Gold Standard," CCSD(T) at the Complete Basis Set Limit? *Journal of Chemical Theory and Computation* **2013**, *9* (5), 2151–2155. DOI: 10.1021/ct400057w.
(46) Helgaker, T.; Klopper, W.; Koch, H.; Noga, J. Basis-set convergence of correlated calculations on water. *The Journal of Chemical Physics* **1997**, *106* (23), 9639–9646. DOI: 10.1063/1.473863 (acccessed 1/10/2024). Neese, F.; Valeev, E. F. Revisiting the Atomic Natural Orbital Approach for Basis Sets:

Robust Systematic Basis Sets for Explicitly Correlated and Conventional Correlated ab initio Methods? *Journal of Chemical Theory and Computation* **2011**, *7* (1), 33–43. DOI: 10.1021/ct100396y.
(47) Riplinger, C.; Neese, F. An efficient and near linear scaling pair natural orbital based local coupled cluster method. *The Journal of Chemical Physics* **2013**, *138* (3). DOI: 10.1063/1.4773581 (acccessed 1/10/2024).
(48) Dunning, T. H., Jr. Gaussian basis sets for use in correlated molecular calculations. I. The atoms boron through neon and hydrogen. *The Journal of Chemical Physics* **1989**, *90* (2), 1007–1023. DOI: 10.1063/1.456153 (acccessed 10/23/2024).
(49) Neese, F. Software update: The ORCA program system—Version 5.0. *WIREs Computational Molecular Science* **2022**, *12* (5), e1606. DOI: https://doi.org/10.1002/wcms.1606.
(50) Neese, F.; Wennmohs, F.; Becker, U.; Riplinger, C. The ORCA quantum chemistry program package. *The Journal of Chemical Physics* **2020**, *152* (22). DOI: 10.1063/5.0004608 (acccessed 10/23/2024).
(51) Neese, F. The SHARK integral generation and digestion system. *Journal of Computational Chemistry* **2023**, *44* (3), 381–396. DOI: https://doi.org/10.1002/jcc.26942.
(52) Červinka, C.; Pádua, A. A. H.; Fulem, M. Thermodynamic Properties of Selected Homologous Series of Ionic Liquids Calculated Using Molecular Dynamics. *The Journal of Physical Chemistry B* **2016**, *120* (9), 2362–2371. DOI: 10.1021/acs.jpcb.5b11070.
(53) Allen, M. P.; Tildesley, D. J. *Computer Simulation of Liquids*; Oxford University Press, 2017.
(54) Rukhin, A. L. Weighted means statistics in interlaboratory studies. *Metrologia* **2009**, *46* (3), 323. DOI: 10.1088/0026-1394/46/3/021.
(55) Frisch, M. J.; Trucks, G. W.; Schlegel, H. B.; Scuseria, G. E.; Robb, M. A.; Cheeseman, J. R.; Scalmani, G.; Barone, V.; Petersson, G. A.; Nakatsuji, H.; et al. *Gaussian 16 Revision B.01*; 2016.
(56) Hoy, A. R.; Bunker, P. R. A precise solution of the rotation bending Schrödinger equation for a triatomic molecule with application to the water molecule. *Journal of Molecular Spectroscopy* **1979**, *74* (1), 1–8. DOI: https://doi.org/10.1016/0022-2852(79)90019-5.
(57) Venkateswarlu, P.; Gordy, W. Methyl Alcohol. II. Molecular Structure. *The Journal of Chemical Physics* **1955**, *23* (7), 1200–1202. DOI: 10.1063/1.1742240 (acccessed 10/23/2024).
(58) Niide, Y.; Hayashi, M. Reinvestigation of microwave spectrum of dimethyl ether and rs structures of analogous molecules. *Journal of Molecular Spectroscopy* **2003**, *220* (1), 65–79. DOI: https://doi.org/10.1016/S0022-2852(03)00076-6.
(59) Červinka, C. Interplay of aprotic ionic liquids with hydrogen bonded networks in associating aliphatic alcohols. *Journal of Molecular Liquids* **2025**, *417*, 126600. DOI: https://doi.org/10.1016/j.molliq.2024.126600.
(60) Wagner, W.; Pruß, A. The IAPWS Formulation 1995 for the Thermodynamic Properties of Ordinary Water Substance for General and Scientific Use. *Journal of Physical and Chemical Reference Data* **2002**, *31* (2), 387–535. DOI: 10.1063/1.1461829 (acccessed 3/13/2025).
(61) Piazza, L.; Span, R. An equation of state for methanol including the association term of SAFT. *Fluid Phase Equilibria* **2013**, *349*, 12–24. DOI: https://doi.org/10.1016/j.fluid.2013.03.024.
(62) Craven, R. J. B.; de Reuck, K. M. Development of a vapour pressure equation for methanol. *Fluid Phase Equilibria* **1993**, *89* (1), 19–29. DOI: https://doi.org/10.1016/0378-3812(93)85043-L.
(63) Ihmels, E. C.; Lemmon, E. W. Experimental densities, vapor pressures, and critical point, and a fundamental equation of state for dimethyl ether. *Fluid Phase Equilibria* **2007**, *260* (1), 36–48. DOI: https://doi.org/10.1016/j.fluid.2006.09.016.
(64) Wu, J.; Magee, J. W. Heat Capacity of Saturated and Compressed Liquid Dimethyl Ether at Temperatures from (132 to 345) K and at Pressures to 35 MPa. *Journal of Chemical & Engineering Data* **2018**, *63* (5), 1713–1723. DOI: 10.1021/acs.jced.8b00037. Baidakov, V. G.; Grishina, K. A.; Kaverin, A. M. Capillary constant and surface tension of dimethyl ether and n-butane at temperatures from 214 K to those close to the critical point. *Fluid Phase Equilibria* **2016**, *414*, 55–59. DOI: https://doi.org/10.1016/j.fluid.2016.01.001.
(65) Peng, D.-Y.; Robinson, D. B. A New Two-Constant Equation of State. *Industrial & Engineering Chemistry Fundamentals* **1976**, *15* (1), 59–64. DOI: 10.1021/i160057a011.
(66) Dias, R. F.; da Costa, C. C.; Manhabosco, T. M.; de Oliveira, A. B.; Matos, M. J. S.; Soares, J. S.; Batista, R. J. C. Ab initio molecular dynamics simulation of methanol and acetonitrile: The effect of van

der Waals interactions. *Chemical Physics Letters* **2019**, *714*, 172–177. DOI: https://doi.org/10.1016/j.cplett.2018.10.085.
(67) Benmore, C.; C., G. L.; and Soignard, E. Intermediate range order in supercooled water. *Molecular Physics* **2019**, *117* (18), 2470–2476. DOI: 10.1080/00268976.2019.1567853.
(68) Skinner, L. B.; Benmore, C. J.; Neuefeind, J. C.; Parise, J. B. The structure of water around the compressibility minimum. *The Journal of Chemical Physics* **2014**, *141* (21). DOI: 10.1063/1.4902412 (acccessed 3/25/2025).
(69) Yamaguchi, T.; K., H.; and Soper, A. K. The structure of liquid methanol revisited: a neutron diffraction experiment at −80 °C and +25 °C. *Molecular Physics* **1999**, *96* (8), 1159–1168. DOI: 10.1080/00268979909483060.
(70) Yamaguchi, T.; K., H.; and Soper, A. K. The structure of liquid methanol revisited: a neutron diffraction experiment at -80 °C and +25 °C. *Molecular Physics* **1999**, *97* (4), 603–605. DOI: 10.1080/00268979909482859.
(71) Medders, G. R.; Babin, V.; Paesani, F. Development of a "First-Principles" Water Potential with Flexible Monomers. III. Liquid Phase Properties. *Journal of Chemical Theory and Computation* **2014**, *10* (8), 2906–2910. DOI: 10.1021/ct5004115.
(72) Del Ben, M.; Hutter, J.; VandeVondele, J. Probing the structural and dynamical properties of liquid water with models including non-local electron correlation. *The Journal of Chemical Physics* **2015**, *143* (5), 054506. DOI: 10.1063/1.4927325 (acccessed 7/4/2025).
(73) Gillan, M. J.; Alfè, D.; Michaelides, A. Perspective: How good is DFT for water? *The Journal of Chemical Physics* **2016**, *144* (13). DOI: 10.1063/1.4944633 (acccessed 6/28/2025).
(74) Del Ben, M.; Schütt, O.; Wentz, T.; Messmer, P.; Hutter, J.; VandeVondele, J. Enabling simulation at the fifth rung of DFT: Large scale RPA calculations with excellent time to solution. *Computer Physics Communications* **2015**, *187*, 120–129. DOI: https://doi.org/10.1016/j.cpc.2014.10.021.
(75) Morrone, J. A.; Tuckerman, M. E. Ab initio molecular dynamics study of proton mobility in liquid methanol. *The Journal of Chemical Physics* **2002**, *117* (9), 4403–4413. DOI: 10.1063/1.1496457 (acccessed 7/10/2025).
(76) Sieffert, N.; Bühl, M.; Gaigeot, M. P.; Morrison, C. A. Liquid Methanol from DFT and DFT/MM Molecular Dynamics Simulations. *Journal of Chemical Theory and Computation* **2013**, *9* (1), 106–118. DOI: 10.1021/ct300784x.
(77) Handgraaf, J. W.; Meijer, E. J.; Gaigeot, M. P. Density-functional theory-based molecular simulation study of liquid methanol. *Journal of Chemical Physics* **2004**, *121* (20), 10111–10119. DOI: 10.1063/1.1809595.
